# Determining the safety and effectiveness of Tai Chi: a critical overview of 210 systematic reviews of controlled clinical trials

**DOI:** 10.1186/s13643-022-02100-5

**Published:** 2022-12-03

**Authors:** Guo-Yan Yang, Jennifer Hunter, Fan-Long Bu, Wen-Li Hao, Han Zhang, Peter M. Wayne, Jian-Ping Liu

**Affiliations:** 1 NICM Health Research Institute, Western Sydney University, Locked Bag 1797, Penrith, NSW 2751 Australia; 2 Health Research Group, Sydney, NSW 2000 Australia; 3 National Center for Children’s Health, Beijing Children’s Hospital, Capital Medical University, Beijing, 100045 China; 4 Public Health School, Inner Mongolia Medical University, Hohht, 010000 Inner Mongolia China; 5 School of Acupuncture and Massage, Beijing University of Chinese Medicine, Beijing, 100029 China; 6 Osher Center for Integrative Medicine, Brigham and Women’s Hospital and Harvard Medical School, Boston, MA 02215 USA; 7 Center for Evidence-Based Chinese Medicine, Beijing University of Chinese Medicine, Beijing, 100029 China; 8 The Faculty of Health Science, Department of Community Medicine, UiT The Arctic University of Norway, The National Research Center in Complementary and Alternative Medicine – NAFKAM, Hansine Hansens veg 19, 9037 Tromsø, Norway

**Keywords:** Tai Chi, Overview, Systematic review, Treatment, Prevention, Rehabilitation

## Abstract

**Background:**

This overview summarizes the best available systematic review (SR) evidence on the health effects of Tai Chi.

**Methods:**

Nine databases (PubMed, Cochrane Library, EMBASE, Medline, Web of Science, China National Knowledge Infrastructure (CNKI), Chinese Scientific Journal Database (VIP), Sino-Med, and Wanfang Database) were searched for SRs of controlled clinical trials of Tai Chi interventions published between Jan 2010 and Dec 2020 in any language. Effect estimates were extracted from the most recent, comprehensive, highest-quality SR for each population, condition, and outcome. SR quality was appraised with AMSTAR 2 and overall certainty of effect estimates with the GRADE method.

**Results:**

Of the 210 included SRs, 193 only included randomized controlled trials, one only included non-randomized studies of interventions, and 16 included both. Common conditions were neurological (18.6%), falls/balance (14.7%), cardiovascular (14.7%), musculoskeletal (11.0%), cancer (7.1%), and diabetes mellitus (6.7%). Except for stroke, no evidence for disease prevention was found; however, multiple proxy-outcomes/risks factors were evaluated. One hundred and fourteen effect estimates were extracted from 37 SRs (2 high, 6 moderate, 18 low, and 11 critically low quality), representing 59,306 adults. Compared to active and/or inactive controls, 66 of the 114 effect estimates reported clinically important benefits from Tai Chi, 53 reported an equivalent or marginal benefit, and 6 an equivalent risk of adverse events. Eight of the 114 effect estimates (7.0%) were rated as high, 43 (37.7%) moderate, 36 (31.6%) low, and 27 (23.7%) very low certainty evidence due to concerns with risk of bias (92/114, 80.7%), imprecision (43/114, 37.7%), inconsistency (37/114, 32.5%), and publication bias (3/114, 2.6%). SR quality was often limited by the search strategies, language bias, inadequate consideration of clinical, methodological, and statistical heterogeneity, poor reporting standards, and/or no registered SR protocol.

**Conclusions:**

The findings suggest Tai Chi has multidimensional effects, including physical, psychological and quality of life benefits for a wide range of conditions, as well as multimorbidity. Clinically important benefits were most consistently reported for Parkinson’s disease, falls risk, knee osteoarthritis, low back pain, cerebrovascular, and cardiovascular diseases including hypertension. For most conditions, higher-quality SRs with rigorous primary studies are required.

**Systematic review registration:**

PROSPERO CRD42021225708.

**Supplementary Information:**

The online version contains supplementary material available at 10.1186/s13643-022-02100-5.

## Background

Tai Chi is a traditional exercise, martial art, and mind–body practice that is practiced by people of different ages and health statuses. Also known as Tai Chi Chuan/Quan or Taiji, Tai Chi originated in China in the seventeenth century A.D. [1]. The practice is low to moderate intensity with repetitive, flowing, meditative movements that aim to cultivate and maintain health and wellbeing [2]. There are five major traditional styles of Tai Chi, namely *Chen*,* Yang*,* Wu*,* Wu/Hao*, and *Sun* styles, along with numerous newer styles, hybrids, and extensions. Tai Chi integrates the essence of Chinese folk and military martial arts, with traditional Chinese medicine theories [3, 4]. The core components of Tai Chi are traditionally described as including sequenced movements, meditative and visualization techniques, and deep, abdominal breathing [3]. In China, Tai Chi is widely taught in high schools and higher education-related organizations [5].

Interest in evaluating the effects of Tai Chi in both healthy populations and people with a wide range of diseases, conditions, and symptoms has steadily increased globally [6, 7]. A bibliometric analysis of clinical studies of Tai Chi published between 1958 and 2013 identified 507 studies, of which 43 (8.3%) were systematic reviews (SRs) of randomized controlled trials (RCTs) and/or non-randomized studies of interventions (NRSIs) [6]. The 2010 to 2020 update identified 987 studies, of which 157 (15.9%) were SRs [7].

Given the large number of SRs of Tai Chi, SRs of SRs (henceforth referred to as overviews) are increasingly being conducted. Some have evaluated multiple interventions for a single condition [8–16], whilst others have focused only on Tai Chi interventions for either a single condition [17–22] or multiple conditions [23–27]. Limitations of the overviews evaluating only Tai Chi interventions [17–27] were the potential for language bias [17, 18, 22, 23, 25–27], reporting bias in which the most favourable results were emphasized [23, 27], and reporting multiple estimates of effects/results for the same or similar outcome and population, with limited or no discussion about conflicting results or overlapping of the primary studies [18–25, 27].

As such, this overview aims to systematically identify and appraise the best available SR evidence reported in the most recent, comprehensive, and/or highest-quality SRs, on the safety and effectiveness of Tai Chi for health promotion and managing disease.

## Methods

The methods were guided by the Cochrane Handbook for Systematic Reviews of Interventions [28], in particular Chapter V: Overview of Reviews [29], the Joanna Briggs Institute Manual for Evidence Synthesis: Chapter 10 Umbrella Review [30], the GRADE (Grading of Recommendations, Assessment, Development and Evaluations) Handbook [31], and the PRISMA (Preferred Reporting Items for Systematic Reviews and Meta-Analyses) 2020 statement [32]. The PRISMA 2020 checklist is presented in Additional file 1.

### Protocol and registration

A protocol was registered prior to data extraction at the International Prospective Register for Systematic Reviews (PROSPERO) (CRD42021225708). Deviations from the protocol prior to formal screening and data extraction were as follows: only partial blinding of the reviewers to the results when selecting SRs and outcomes, including important secondary outcomes of a SR, reporting more than three outcomes for some populations; and including SRs of NRSIs.

### Populations

All populations were included, regardless of health status, setting, location, and country.

### Interventions

All exercise programs described as Tai Chi were included. No limitations were applied to Tai Chi styles (such as *Chen*,* Yang*,* Wu*, *Wu/Hao*, and *Sun* style) or forms (such as 6-form, 24-form, 54-form, and 83-form Tai Chi). Exercise programs that combined Tai Chi with other interventions such as Qigong, meditation, or conventional exercise were only included if the reviewers clarified that Tai Chi was the core component. A SR that evaluated Tai Chi and other interventions (e.g. any form of exercise) was excluded if the effects of Tai Chi was not analysed in a separate analysis.

### Comparisons

Any type of control was included, for example, no intervention, waitlist control, usual care, and active control. When the data was available, the pooled effects according to control group categories were extracted to reduce clinical and methodological diversity. Comparisons also include a co-intervention if applied in all arms.

### Outcomes

Any outcome was eligible for inclusion. However, as much as possible, the number of outcomes extracted per population/comparison group was limited to three. These were selected to reflect the SR’s primary/main outcome(s), outcomes that align with the reasons why people use Tai Chi and what matters to them, the validity/reliability of the measurement tool, and directness of the outcome measure to health status (e.g. clinical outcomes in preference to risk factors). Core outcome sets and other resources such as those published on the Core Outcome Measures in Effectiveness Trials (COMET) Initiative database [33] were used to inform these decisions. Two senior reviewers (GYY, JH) jointly made these decisions. When estimates of effect were reported for multiple timepoints, the timepoints with the most RCTs was selected. Additional timepoints were only selected if the studies were not included in the first estimate.

### Study designs

All SRs of interventions, with or without a meta-analysis of RCTs, quasi-RCTs, and other NRSIs (e.g. cohort studies, case–control studies, controlled before-and-after studies, interrupted-time-series studies, case series and case reports), were included. Whilst SRs of RCTs were likely to provide the most reliable evidence for most estimates of effect, SRs of NRSIs were also included (post protocol, pre-data extraction) in the circumstance when this was the best available evidence.

### Literature search

The search strategy built upon a bibliometric analysis of Tai Chi intervention studies published between 1^st^ January 2010 and 31^st^ January 2020 [7]. The search was updated for the purpose of this overview (1^st^ January to 12^th^ December 2020) using the same search terms and databases—PubMed, Cochrane Library, EMBASE, Medline, Web of Science, China National Knowledge Infrastructure (CNKI), Chinese Scientific Journal Database (VIP), Sino-Med, and Wanfang Database (Additional file 2). The search strategies were developed and refined by the team of experts who conducted an earlier bibliometric analysis [6]. Tai Chi search terms include “Taiji”, “Tai Ji”, “Tai-ji”, “Tai Chi”, “Tai Chi Chuan”, “Tai Chi Quan”, or “Taijiquan”. Limitation to language and publication status was not applied. Grey literature was included. Database searches were augmented with bibliography searches of other recently published SRs of SRs [8–27].

### Study selection

The search results from English databases were exported into EndNote (version X9), and those from Chinese databases into NoteExpress (version 3.2). Duplicates were removed before study selection. Following calibration exercises, reviewers (GYY, JH, WLH, HZ) worked in pairs to independently screen the title/abstracts and full texts. Two reviewers (GYY, JH) rescreened the full texts of the 157 SRs (106 published in English, 41 published in Chinese) that were identified in the 2020 bibliometric analysis [7]. Final decisions were made by consensus and involved other reviewers when necessary.

To minimize overlap of primary studies, one SR for each population, condition, or outcome (PCO) was then selected for the final evidence synthesis. A staged approach was applied to selecting this subset of SRs with the aim of identifying the most recent, comprehensive, and highest-quality SR for each PCO. First, SRs with a meta-analysis of RCTs were grouped according to their PCO, from which the publication date and number of RCTs were compared. When multiple SRs were published within 4–5 years of each other and/or the number of RCTs were similar, a single reviewer (GYY, JH) extracted further data about the number of databases searched, any language restrictions, the primary/main outcomes, and the number of RCTs and overlapping RCTs per meta-analysis. An informal appraisal of the SR quality using AMSTAR 2 [34] was also done. Finally, SRs without a meta-analysis were then screened, and SRs that included a meta-analysis of NRSIs were rescreened to ensure there were no missing PCO.

### Data collection

A pre-defined data extraction form that was an extension of the bibliometric analysis extraction form was designed and piloted by two reviewers (GYY, JH). Data extraction was staged for pragmatic reasons and to partially blind the investigators when selecting the SRs and PCO. For all included SRs, information about the characteristics of the studies (i.e. citation details, authors, study design, number of RCTs and NRSIs, participants characteristics, and types of outcomes) were extracted. For the subset of SRs selected for the final evidence synthesis (and those when SR selection could not be made based on the preliminary data extraction), additional information about the search strategy, study characteristics of included studies, and the SR quality was also extracted. For each estimate of effect that was selected for the final evidence synthesis, additional information about the participants, settings, estimates of effect, statistical heterogeneity, subgroup and sensitivity analysis, and publication bias was then extracted. Estimates of effect were not extracted for the SRs with no meta-analysis as this would require extracting data from the original publications of the primary studies, nor for a meta-analysis that did not meet the criteria outlined in item 11 of AMSTAR 2. Following calibration exercises, five reviewers (GYY, WLH, FLB, HZ, JH) extracted data into Research Electronic Data Capture (REDCap) [35] that was verified by two senior reviewers (GYY, JH). Final decisions were made by consensus with the review team.

### Quality assessment

Only the subset of SRs included in the final evidence synthesis were formally assessed for quality using AMSTAR 2 (A MeaSurement Tool to Assess systematic Reviews, improved version) critical appraisal tool and rated as high, moderate, low, or critically low quality [34]. Items 2, 4, 9, 11, 13, and 15 were deemed critical. Item 7, which requires the list of the excluded articles with the rationale is reported, was introduced to AMSTAR 2 in late 2017. A similar reporting requirement was introduced to the revised PRISMA 2020 statement published in early 2021 [32]. Consequently, for the purpose of this review item 7 was deemed non-critical. Additionally, SRs published before 2019 were not downrated for item 7 if they met the accepted reporting standards for excluded articles as per PRISMA 2009 [36]. For all other items, the AMSTAR 2 guidance was followed. A sensitivity analysis was conducted to compare this modified AMSTAR 2 rating for item 7 with the original guidance.

GRADE guidelines [31] and GRADEpro GDT software [37] were used to rate the overall certainty (quality) of the evidence for the extracted effect estimates. Due to pragmatic constraints, assessments of the risk of bias of the primary studies, heterogeneity, and publication bias relied upon the assessments reported in the SR. However, the results of any sensitivity analyses were extracted and considered. Given the large number of SRs, evaluating a wide range of populations and outcomes, a pragmatic approach similar to that used by Pollock et al. [38] was applied where specific thresholds, ranges, and criteria were established and piloted to optimize consistency and transparency across all the ratings. The details of the rubric used to inform the GRADE assessments are reported in Supplementary File 6 and summarized below.

For the risk of bias (RoB) assessments, randomization/selection bias, assessor blinding, and missing data were deemed the most important categories. This decision reflected the need to select domains assessed by the RoB assessment tools used in the SRs and that it is not possible to blind Tai Chi study participants. For there to be no serious concerns with RoB, at least 75% of the included RCTs in the SR had a low RoB in each of these three categories.

Inconsistency was investigated when the *I*^*2*^ test for statistical heterogeneity was ≥ 75%. This involved inspecting the Forest plot for overlapping 95% confidence intervals (CI) and direction of effects, and the findings from any subgroup or sensitivity analysis reported in the SR. In a post hoc sensitivity analysis, inconsistency was investigated if the *I*^*2*^ test was ≥ 30% or τ^*2*^ test *p* ≥ 0.1.

Since all participants, interventions, and outcomes were directly relevant to the research question, all estimates of effect were automatically rated as having no serious concerns with indirectness.

Assessments of imprecision were according to whether the optimum information size was likely to be met, the width of the 95% CI, and whether important benefits and/or harms could be excluded. Due to pragmatic constraints, unless reported otherwise in the SR, thresholds were set for optimum information sizes [31, 38]. In a post hoc sensitivity analysis, the threshold for the optimum information size for continuous data was increased from 200 [38] to 400 [31]. For standardized mean differences (SMD), the minimal clinically important difference (MCID) for important benefit was set at 0.5 that is considered to be a moderate effect size, and a large effect size was set at 0.8 [39]. For mean differences (MD), the MCID for important benefit was based on studies involving similar populations [40–59]. For relative risks (RR), the cut-off for important benefits was set at < 0.75 or > 1.25. For risk differences (RD), the cut-off for important harm was set at ± 0.1 for non-serious AEs and ± 0.01 for serious AEs.

Publication bias was only considered when at least ten RCTs were in the meta-analysis. In instances when the SR did not report on the publication bias for an effect estimates yet assessed it for another, the findings from that assessment were applied. If there was no information, at least half of the studies had to have a sample size larger than 100 for there to be no serious concerns about publication bias.

Following calibration exercises, the AMSTAR 2 assessments were independently made by two reviewers in pairs (GYY, JH, FLB) and the GRADE certainty assessments were made by one of these reviewers and verified by a second reviewer. Final decisions were made by consensus with the team.

### Synthesis of results

The results are narrated and presented in tables, including a summary of findings table for all estimates of effect. Dichotomous data are presented as RR or RD and number needed to treat (NNT), with 95% confidence intervals (CIs). When available, rates are presented as the number of participants. Continuous data are presented as weighted MD or SMD, with 95% CIs. No further meta-analysis, network analysis, or re-analysis of the results was conducted.

## Results

### Search results

**Fig. 1:**
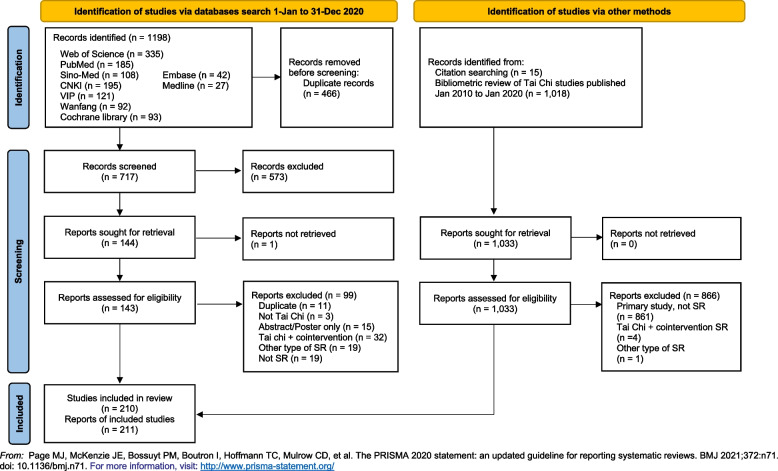
PRISMA flow diagram

The literature searches identified 210 eligible SRs (211 articles) of Tai Chi (Fig. 1). The citations with the reason for excluding 100 full-text articles are listed in Additional file 3.

### Study selection for evidence synthesis

From the 210 SRs of Tai Chi, 47 SRs [60–106] were selected for the final evidence synthesis and 114 estimates of effect, representing 59,306 adult participants in RCTs, were extracted from 37 SRs [61, 62, 64, 66–68, 70, 71, 73–75, 77, 79–88, 90–94, 96–98, 100–106]. Estimates of effects were not extracted, and the GRADE certainty of the evidence was not appraised for four SRs with unreliable meta-analyses [65, 76, 78, 99] and six SRs with no meta-analysis [60, 63, 69, 72, 89, 95]. No results were extracted from, nor was the AMSTAR-2 quality formally appraised or reported for 163 SRs (164 articles) because for 79 SRs, a far more recent SR, typically with more primary studies, was identified; for 46 SRs (47 articles) following further consideration, a SR of higher quality and/or with more primary studies in the meta-analysis for the PCO was selected; and for 38 of the SRs that did not conduct a meta-analysis, the PCO were reported by a SR with a meta-analysis. When the analysis of this overview has been finalised, we found an erratum for an included SR with a meta-analysis on fear of falling was published on 3^rd^ September 2022 [107], which corrects the error that led a misinterpretation of their methodology and findings because a meta-regression was performed with the SMD as the dependent variable. As a result, the comparison we included from this SR was Tai Chi with and without supervision by a Tai Chi instructor, which is not eligible for inclusion. The SR was still included as the corrections did not alter the overall assessment of the certainty of the evidence for that outcome. The citations and reasons for excluding the 163 potentially eligible SRs from the evidence synthesis are reported in Additional file 4.

### Characteristics of studies

Since 2010, the number of SRs published each year in English and Chinese databases rose exponentially (Table 1). Most were SRs with a meta-analysis of RCTs (78.6%, 165/210) and were published in English (73.8%, 155/210) or Chinese (25.7%, 54/210). The first author of 139 (66.2%) SRs was from a university/institution located in mainland China, Hong Kong, or Taiwan. The median number of participants per SR was 750, ranging from 42 to 9263. Only 18 (8.6%) SRs included studies in which at least some of the study participants were under 25 years of age. Multiple outcomes measuring the effects of Tai Chi in a wide range of populations were evaluated. The most common conditions and their associated risks factors were for cardio/cerebrovascular diseases and falls. One SR specifically evaluated the risk of adverse events.

**Table 1:** Characteristics of systematic reviews of Tai Chi interventions

**Number of systematic reviews (SRs)**	210
Meta-analysis	165
Narrative analysis only	45
**Studies included in SRs**	210
RCTs only	193
NRSIs only	1
Both RCTs and NRSIs	16
**RCTs per SR:** Median (range)	9 (1–77)
**NRSIs per SR:** Median (range)	4 (1–18)
**Participants per SR:** Median (range)	750 (42–9263)
**Publication year**	
2010–2012	20
2013–2014	33
2015–2016	37
2017–2018	54
2019–2020	66
**Publication language**	
English	155
Chinese	54
Korean	1
**Country / region of first author**	
China, Hong Kong, Taiwan	139
United States of America, Canada, Brazil	35
Western Europe	19
South Korea, Singapore, Thailand	10
Australia, New Zealand	6
**Funding**	
Government / national grants	84
University	15
Charity	3
No information	108
**Disease / condition**	
Healthy adolescents/ adults	8
Multiple chronic diseases	9
Falls, balance, other falls risk factors	32
Hypertension	15
Cancer (breast cancer *n* = 8)	15
Diabetes mellitus	14
Cognitive impairment	13
Parkinson’s disease	13
Chronic obstructive pulmonary disease	13
Cerebrovascular disease (stroke)	11
Mental health	11
Ischaemic heart disease	10
Osteoarthritis (knee *n* = 5)	10
Osteoporosis / osteopenia	8
Heart failure	6
Sleep disorders / quality	6
***3 SRs each for:*** Low back pain, chronic pain	
***2 SRs each for:*** Multiple sclerosis, rheumatoid arthritis	
***1 SR each for:*** Hyperlipidaemia, fatigue, fibromyalgia, frailty, immunity/HIV infection, adverse effects	

*RCT* randomized controlled trial, *NRSI* non-randomized studies of interventions, *QoL* quality of life

Table 2 summarizes the characteristics of the 47 SRs (41 SRs with meta-analysis and 6 SRs without meta-analysis) included in the final evidence synthesis. Of note, only two SRs included adolescents [95, 100] and 40 included older adults (≥ 60 years). Almost all study participants were living in independently in the community. Most SRs included participants from both Asian and non-Asian countries. Only two SRs were limited to Chinese participants only [103, 104].

**Table 2:** Characteristics of systematic reviews included in the evidence synthesis

Review ID	Populations (no.) Conditions	Main outcomes * (Other outcomes)	Tai Chi (TC) minutes/session, frequency/week, follow-up Comparisons	Search date No. databases No. studies (Languages)	Funding	SR analysis AMSTAR 2 quality rating
Buto 2020 [60]	Older adults (*n* = 2063; TC = 1728) Prefrail and frail	Functional capacity, QoL	20–60 min 1–5 times/week, 15–96 weeks TC vs Ucare, HEd, PT, Ex, other	Sep 2019 6 databases (Eng.) 15 RCTs (Tai Chi: 9 RCTs) (Eng. Chin.)	Uni	No meta-analysis Moderate (†*Low*)
Cheng 2019 [61]	Adults (*n* = 657) Fibromyalgia	Fibromyalgia impact (fatigue, pain, sleep quality, depression, QoL)	55–120 min 1–3 times/week, 10–24 weeks TC vs Ucare, Ex	May 2019 3 databases (Eng.) 6 RCTs (Eng.)	Uni	Meta-analysis Low (†*Critically low*)
Choo 2020 [62]	Older adults (*n* = 869) Chronic diseases	NI (QoL, mental health, physical function, AE)	40–90 min 1–4 times/week, 10–24 weeks TC vs noRx, Ucare, HEd, Pharm, Ex, other, waitlist	NI 7 databases (Eng. Chin.) 13 RCTs (Eng. Chin.)	NI	Meta-analysis Critically low
Cocchiara 2020 [63]	Adults (*n* = 467) Workplace wellness of healthcare workers	NI (work-related stress, physical and mental health, improvement in attention and/or productivity)	8–12 weeks NI	Mar, 2019 4 databases (Eng.) 1 SR, 3 RCTs, 1 NRSI, 1 case report (Eng.)	NI	No meta-analysis Critically low
Cui 2019 [64]	Adults, older adults (*n* = 1794) Healthy, chronic diseases	Serious and non-serious adverse events	30–60 min 1–5 times/week, 12–48 weeks TC vs active or inactive intervention	Feb 2016 3 databases (Eng.) 24 RCTs (Eng.)	Gov	Meta-analysis Critically low
Dong 2020 [65]	Adults, older adults (*n* = 1608) with or without hypertension	Systolic and diastolic BP	30–120 min 1–7 times/week, 8–24 weeks TC vs noRx, Ex	Jan 2019 6 databases (Eng. Chin.), 24 RCTs (Eng. Chin.)	Gov	Meta-analysis Critically low
Gu 2017 [66]	Adults, older adults (*n* = 918) Chronic heart failure	6MWD, QoL (B-type natriuretic peptide, LVEF, BP, VO_2_peak)	15–60 min 2–7 times/week, 4–24 weeks TC vs Ucare, Ex, Pharm	Jun 2016 6 databases (Eng. Chin.) 13 RCTs (Eng. Chin.)	Gov	Meta-analysis Low (†*Critically low*)
Guo 2020 [67]	Adults, older adults (*n* = 1508) COPD	Lung function tests (6MWD, QoL)	30–60 min 2–5 times/week, 8–48 weeks TC vs noRx, Ex	Aug 2018 9 databases (Eng. Chin.) 16 RCTs (Eng. Chin.)	Gov	Meta-analysis Low
Hall 2017 [68]	Adults, older adults (*n* = 1012) Musculoskeletal chronic pain	Pain (disability, stiffness, QoL)	40–60 min 2 times/week, 6–20 weeks TC vs Ucare, HEd, waitlist	Nov 2015 7 databases (Eng.) 15 RCTs (Eng.)	NI	Meta-analysis Low (†*Critically low)*
Ho 2013 [69]	Adolescents, adults, older adults (*n* = 939) Healthy, HIV	NI (any symptoms of infections, biomedical indicators of immunity)	30–60 min 1–5 times/week, 5–576 weeks TC vs Ucare, HEd, Ex, waitlist	Jan 2011 14 databases (Eng. Chin.) 7 RCTs, 9 NRSIs (Eng. Chin.)	NI	No meta-analysis Low (†*Critically low)*
Hu 2020 [70]	Older adults (*n* = 986) Knee osteoarthritis	Pain, stiffness, physical function, mental health, self-efficacy, AE	30–60 min 1–4 times/week, 5–52 weeks TC vs noRx, Ucare, HEd, PT, waitlist	Jun 2020 9 databases (Eng. Chin.) 16 RCTs (Eng. Chin.)	No	Meta-analysis Low (†*Critically low)*
Huang 2017 [73]	Older adults (*n* = 3824) Chronic diseases	No. of people who fell (falls incidence)	1–5 times/week, 16–96 weeks TC vs noRx, PT, Ex, waitlist	Feb 2016 3 databases (Eng.) 18 RCTs (Eng. Chin.)	Gov	Meta-analysis Low (†*Critically low*)
Huang 2019 [72]	Adults, older adults (*n* = 132) Vestibular balance disorders	Balance, gait, neuromuscular function, trunk stability (symptom severity/relief/impact, AE)	45–90 min 1–5 times/week, 3–18 weeks TC vs noRx, vestibular training/education, Ex; TC individualized vs TC standardized	Dec 2016 4 (English) 3 RCTs, I NRSI (English)	NI	No meta-analysis Low (†*Critically low*)
Huang 2020 [71]	Adults, older adults (*n* = 3842) Falls	Falls rate (Balance—single leg standing, Berg balance scale, time-up-and-go)	30–90 min 1–7 times/week, 8–48 weeks TC vs ADL, PT, HEd, Ex, other	Aug 2019 6 databases (Eng. Chin.) 20 RCTs (Eng. Chin.)	Gov	Meta-analysis Critically low
Jiang 2018 [74]	Older adults (*n* = 290) Stable angina	NI (VO_2_max, maximum heart rate)	12–48 weeks TC vs noRx, Ex	1999–2015 10 databases (Eng. Chin.) 5 RCTs (Eng. Chin.)	NI	Meta-analysis Low (†*Critically low*)
Kruisbrink 2020 [75]	Older adults (NI) Falls	Fear of falling	30–60 min 2–7 times/week, 8–24 weeks TC vs noRx, Ucare	Jul 2005–Jul 2019 5 databases (Eng.) 62 RCTs / 38 Tai Chi (Eng.)	Uni	Meta-analysis Low (†*Critically low*)
Liu F 2020 [76]	Adults (*n* = 772; TC = 179) Substance abuse disorders	Depression, anxiety (AE)	45–60 min 3–4 times/week, 45–60 weeks TC vs usual care	Jan 2019 (English Chinese) 1 RCT, 6 NRSIs (English, Chinese)	Uni	Meta-analysis Critically low
Liu LZ 2020 [77]	Adults, older adults (*n* = 1268) Breast cancer	Fatigue, sleep quality, QoL	30–120 min 2–3 times/week, 10–24 weeks TC vs Ucare, Psych, sham QiGongEx; TC + Rehab vs Dance + Rehab	Jun 2019 8 databases (Eng. Chin.) 16 RCTs (Eng. Chin.)	n.f.p	Meta-analysis Critically low
Liu WJ 2020 [78]	Older adults (*n* = 798) Osteoarthritis	NI (Pain, stiffness & function, time-up-and-go, 6MWD, other)	8–48 weeks TC vs Ucare, HEd, other	Jan 2019 6 databases (Eng. Chin.) 15 RCTs (Eng. Chin.)	NI	Meta-analysis Critically low
Luo 2020 [79]	Female adults (*n* = 885) Breast cancer	Quality of life (fatigue, BMI, laboratory blood tests)	20–120 min 3–7 times/week, 12–24 weeks TC vs Psych; TC + Ucare vs Ucare; TC + Rehab vs Rehab	Feb 2020 11 databases (Eng. Chin. Japanese Korean Tai) 15 RCTs (Eng. Chin.)	Gov	Meta-analysis Moderate (†*Low*)
Lyu 2018 [81]	Adults, older adults (*n* = 1293) Stroke survivors	NI (ADL, motor function, balance, mental health, AE)	20–48 weeks TC vs Rehab	Oct 2017 7 databases (Eng. Chin.) 21 RCTs (Eng. Chin.)	Gov	Meta-analysis Moderate (†*Low*)
Lyu 2020 [80]	Adults, older adults (*n* = 723) Stroke survivors	NI (mental health, sleep quality, cognitive impairment)	6–48 weeks TC vs Ucare, Rehab; TC + Rehab vs Rehab	Jul 2020 7 databases (Eng. Chin.) 11 RCTs (Eng. Chin.)	Gov	Meta-analysis Moderate (†*Low*)
Mudano 2019 [82]	Adults, older adults (*n* = 345) Rheumatoid arthritis	NI (pain, disease activity, physical function, AE)	7–60 min 1–2 times/week, 8–12 weeks TC vs Ucare, HEd, Ex; TC + HEd + other vs TCM; TC + HEd class vs HEd brochure	Sep 2018 5 databases (Eng.) 2 RCTs, 5 NRSIs (Eng. Chin.)	NI	Meta-analysis High
Ni 2019 [83]	Adults, older adults (*n* = 1410) Cancer	QoL (limb function, fatigue, sleep quality, laboratory blood tests, AE)	20–60 min 3–5 times/week, 12–24 weeks TC vs Ucare, Rehab, HEd, Psych, Ex, sham Qigong	Apr 2018 7 databases (Eng. Chin.) 22 RCTs (Eng. Chin.)	Gov	Meta-analysis Low (†*Critically low*)
Pan 2016 [84]	Adults, older adults (*n* = 445) Hyperlipidaemia	Lipid measures—cholesterol, triglycerides, LDL-C, HDL-C (AE)	40–60 min 3–7 times/week, 12–48 weeks TC vs Ucare, Ex, waitlist	Jun 2015 6 databases (Eng. Chin.) 6 RCTs (Eng.)	NI	Meta-analysis Low (†*Critically low*)
Qin 2019 [85]	Adults, older adults (*n* = 959) Back pain	Pain, disability (AE)	40–60 min 1–6 times/week, 2–28 weeks TC vs noRx; TC + HEd vs HEd; TC + massage vs massage; TC + PT vs PT	Mar 2019 6 databases (Eng. Chin.) 10 RCTs (Eng. Chin.)	NI	Meta-analysis Low (†*Critically low*)
Si 2020 [86]	Adults, older adults (*n* = 1858) Healthy, chronic diseases	Sleep quality assessed with Pittsburgh Sleep Quality Index (AE)	5–120 min 1–5 times/week, 8–36 weeks TC vs noEx, ADL, HEd, Psych, Ex, Rehab, other, waitlist, sham Qigong	Aug 2019 6 databases (Eng. Chin.) 25 RCTs (Eng. Chin.)	Gov	Meta-analysis Low (†*Critically low*)
Song 2018 [87]	Adults, older adults (*n* = 373) Cancer	Cancer related fatigue	30–60 min 2–7 times/week, 4–12 weeks TC vs Ucare, HEd, Psych, Ex, sham Qigong	Apr 2017 9 databases (Eng. Chin.) 6 RCTs (Eng. Chin.)	Gov	Meta-analysis Low (†*Critically low*)
Su 2020 [88]	Adults, older adults (*n* = 762) Healthy, osteoarthritis	Knee strength	2–7 times/week, 12–72 weeks TC vs noEx, ADL, HEd, Ex	NI 7 databases (Eng. Chin.) 12 RCTs (Eng. Chin.)	Gov	Meta-analysis Critically low
Taylor 2017 [89]	Adults (*n* = 193) Multiple sclerosis	Balance, gait, flexibility, strength, fatigue, QoL, symptoms, perception of disease, mood (AE)	40–90 min TC vs noRx, Ucare, TC + meditation	Aug 2016 12 databases (Eng.) 3 RCTs, 5 NRSIs (Eng.)	NI	No meta-analysis Low (†*Critically low*)
Taylor-Piliae 2020 [90]	Adults, older adults (*n* = 1853) Hypertension, chronic heart failure	Quality of life, mental health (AE)	30 min, 6–52 weeks TC vs noRx, Ucare, PT, Rehab, Ex TC + HEd vs noRx; TC + Rehab vs Rehab; TC + Ex vs Ex	Jul 2009–Jul 2019 10 databases (Eng.) 13 RCTs, 2 NRSIs (Eng.)	No	Meta-analysis Critically low
Wang 2010 [93]	Adults, older adults (*n* = 3817) Healthy, chronic diseases, depression, frail	NI (Stress, anxiety, depression, self-esteem, mood, AE)	30–120 min 1–4 times/week, 10–24 weeks TC vs ADL, HEd, Ex, Psych, waitlist	Mar 2009 11 databases (Eng. Chin.) 17 RCTs, 7 CCS, 16 CS (Eng.)	NI	Meta-analysis Critically low
Wang 2017 [92]	Adults, older adults (*n* = 344) Females, perimenopause	QoL, 8 domains assessed by SF-36 (BMD)	30–90 min 3–7 times/week, 20–36 weeks TC vs Ucare, placebo	Jan 2014 4 databases (Eng. Chin.) 5 RCTs (Eng. Chin.)	Gov	Meta-analysis Critically low
Wang 2020 [91]	Older adults (*n* = 1170) Healthy, chronic diseases	Quality of life	15–90 min 2–5 times/week, 4–24 weeks TC vs Ucare, ADL, Ex	Dec 2019 6 databases (Eng. Chin.) 10 RCTs (Eng. Chin.)	NI	Meta-analysis Critically low
Wayne 2014 [94]	Older adults (*n* = 2553) Cognitive impairment	Global cognitive function, executive function (other cognitive tests, AE)	20–60 min 1–4 times/week, 10–48 weeks TC vs noRx, HEd, Ex	Mar 2013 4 databases (Eng.) 11 RCTs, 5 NRSIs, 4 CS (Eng.)	NI	Meta-analysis Critically low
Webster 2016 [95]	Adolescents (*n* = 9263)	Physical flexibility, depression, anxiety, interpersonal (other physical/mental health)	NI	Feb 2013 11 (Eng. Chin.) 12RCTs, 18NRSIs, 19CS (Eng. Chin.)	NI	No meta-analysis Critically low
Wu 2020 [96]	Adults, older adults (*n* = 615) Myocardial infarction	6-min walk distance, left ventricular ejection fraction (AE)	5–60 min 3–5 times/week, 12–44 weeks TC + HEd + Pharm vs HEd + Pharm; TC + HEd + Ex vs HEd + ADL; TC + HEd + Ex vs HEd + Ex; TC + Ex + Rehab vs + Ex + Rehab	Jan 1976–May 2019 6 databases (Eng. Chin.) 7 RCTs (Chin.)	NI	Meta-analysis Low (†*Critically low*)
Xiang 2017 [97]	Adults, older adults (*n* = 689) Fatigue no serious ailment, chronic disease	Fatigue (vitality, depression, sleep, AE)	40–60 min 1–7 times/week, 12–24 weeks TC vs noRx, Ucare, HEd, Ex, other, sham Qigong	Apr 2016 7 databases (Eng. Chin.) 10 RCTs (Eng. Chin.)	Gov	Meta-analysis Moderate (†*Low*)
Yin 2014 [98]	Adults, older adults (*n* = 2765 TC = 1435) Anxiety, depression and/or chronic diseases	Depression, anxiety (AE)	30–120 min 1–5 times/week, 4–48 weeks TC vs noRx, Ex, sham/other	April 2013 (English) 25 RCTs (English)	NI	Meta-analysis Low
Yu 2019 [99]	University students (*n* = 325) Depression or symptoms	Depression	60 min 2–3 times/week, 7–18 weeks TC vs noRx, Ex	Oct 2016 8 databases (Eng. Chin.) 12 RCTs (Eng. Chin.)	Gov	Meta-analysis Critically low
Yu 2018 [100]	Older adults (*n* = 556) Parkinson’s disease	Motor manifestations, balance, walking ability, QoL	45–60 min 2–10 times/week, 4–24 weeks TC vs noRx, Ex; TC + Ucare vs Ucare; TC + Ucare vs Ex + Ucare	Dec 2018 6 (English Chinese) 7 RCTs (English, Chinese, Korean)	Gov	Meta-analysis Critically low
Zhang 2019 [101]	Adults, older adults (*n* = 857) Osteoporosis, or osteopenia	Osteoporosis-related fractures (BMD, pain, QoL, biochemical markers)	30–120 min 1–6 times/week, 16–48 weeks TC vs noRx, Ucare; TC + Ucare vs Ucare	Sep 2017 7 databases (Eng. Chin.) 15 RCTs (Eng. Chin.)	NI	Meta-analysis Moderate (†*Low*)
Zhang 2020 [102]	Older adults (*n* = 1068) Mild cognitive impairment	Global cognitive function assessed by MMSE (delayed recall test, digit span test)	5–50 min 3–4 times/week, 12–52 weeks TC vs Ucare, ADL, HEd, Psych, Ex	Feb 2020 9 databases (Eng. Chin.) 7 RCTs (Eng.)	NI	Meta-analysis Low (†*Critically low*)
Zheng 2015 [103]	Adults, older adults (*n* = 2393) Stroke (prevention)	Incidence of fatal & nonfatal stroke (stroke risk factors (body weight, BP, lipids, glucose), AE)	30–120 min 3–21 times/week, 4–96 weeks TC vs noRx; TC + Ucare vs Ucare	Oct 2013 6 databases (Eng. Chin.) 23 RCTs, 11 NRSIs, 2 CS (Chin.)	NI	Meta-analysis Low (†*Critically low*)
Zheng 2016 [104]	Adults, older adults (*n* = 483) Schizophrenia, in hospital or other residential care	Positive and negative symptoms assessed by PANSS (social, cognitive, behavioural, stress, discontinuation rate, AE)	45–60 min 2–7 times/week, 12–24 weeks TC + (Pharm + / − Ucare) vs Pharm + (noEx, Ucare, HEd, Ex, or waitlist)	Aug 2016 9 databases (Eng. Chin.) 6 RCTs (Eng. Chin.)	Uni	Meta-analysis Moderate (†*Low*)
Zhong 2020 [105]	Adults, older adults (*n* = 2937) Essential hypertension	Systolic and diastolic BP (AE)	30–120 min 3–14 times/week, 6–240 weeks TC vs noRx, HEd, Ex, other activities; TC + HEd vs HEd; TC + Pharm vs Pharm; TC + HEd vs Pharm + HEd; TC + HEd + Pharm vs Ex + HEd + Pharm; TC + HEd vs Qigong + HEd	Jan 2020 9 databases (Eng. Chin.) 28 RCTs (Eng. Chin.)	Gov	Meta-analysis High
Zhou 2019 [106]	Adults, older adults (*n* = 1235) Type 2 diabetes	Fasting glucose, glycosylated haemoglobin (HbA1c), fasting insulin, insulin resistance, BMI, BP	15–120 min 2–14 times/week, 4–24 weeks TC vs Ucare, Ucare + TCM, HEd, sham exercise	Mar 2018 7 databases (Eng. Chin.) 23 RCTs (Eng. Chin.)	NI	Meta-analysis Critically low

*NI* no information, *Age groups* adolescents 10–18 years, adults 25–59 years, older adults ≥ 60 years, *AE* adverse effects, *6MWD* 6-min walk distance/test, *BMD* bone mineral density, *BMI* body mass index, *BP* blood pressure, *COPD* chronic obstructive pulmonary disease, *HDL-C* high-density lipoprotein cholesterol, *LDL-C* low-density lipoprotein cholesterol, *LVEF* cardiac left ventricular ejection fraction, *MMSE* Mini-Mental State Examination, *PANSS* positive and negative syndrome scale, *QoL* quality of life, *ADL* routine activities of daily living/ routine lifestyle, *Ex* exercise (any type, including stretching), *HEd* health education or other educational interventions, *noRx* no treatment, control, *Pharm* pharmaceutical drugs / medication, *Psych* psychological interventions, counselling, support, *PT* physical therapy/physiotherapy, *Rehab* rehabilitation programs, *TC* Tai Chi intervention, *TCM* traditional Chinese herbal medicine, *Ucare* usual care, conventional treatment, standard medical care

^*^reported as NI if no clear statement about primary outcome(s) of interest

Sensitivity analysis shows different rating

For AMSTAR-2 quality rating refer to Additional File 5

### Quality of studies

According to AMSTAR 2 quality rating, two (4%) of the 47 SRs were rated as ‘High’ [82, 105], seven (15%) as ‘Moderate’ [60, 79–81, 97, 101, 104], 20 (43%) as ‘Low’ [61, 66–70, 72–75, 83–87, 89, 96, 98, 102, 103], and 18 (38%) as ‘Critically low’ [62–65, 71, 76–78, 88, 90–95, 99, 100, 106] (Table 2, Table 3, and Additional file 5). Notably, only four SRs (9%) clearly stated a rationale for the study design inclusion/exclusion criteria (item 3), five (11%) reported the funding details of the included studies (item 10); five (11%) listed the articles excluded at full-text screening (item 10); and 17 (40%) had registered or published a protocol (item 2). Other common deficiencies were not adequately considering and/or discussing how the risk of bias of individual studies might impact the results (items 12), and/or not adequately considering or examining statistical, methodological, or clinical heterogeneity (items 13). Six SRs used the PEDro Scale [107] and another six, Jadad, and whilst both are well regarded risk of bias assessment tools, they do not ask about selective reporting bias that is a requirement for full marks for item 9. However, even if full marks were awarded, a sensitivity analysis confirmed this would not have changed their overall ratings. In contrast, a sensitivity analysis found that if item 7 was added to the critical item list and no concessions for SRs published before 2019 was applied, then despite having met the 2009 PRISMA reporting standards for excluded articles [108], only five (11%) of the systematic reviews would have met the criteria. Consequently, an additional seven SRs would be downrated from moderate to low quality [60, 79–81, 97, 101, 104] and 18 from low to critically low quality [61, 66, 68–70, 72–75, 83–87, 89, 96, 102, 103] (Additional file 5).

**Table 3:** Summary of findings of the health effects of Tai Chi

Study ID	Populations; settings; countries			
***AMSTAR-2*** ***SR quality***	**Outcome, subgroup population** **(no. studies: no. overlapping studies)**	**Intervention vs Comparisons** **(no. participants)**	Estimate of effect* (95% CI) ***Effect size***	**GRADE certainty**
**Adverse events (AE)**				
	Adults, older adults; healthy, obesity, cancer, myocardial infarction, chronic heart failure, osteoarthritis, type 2 diabetes mellitus, chronic pulmonary disease; in community settings; China, South Korea, Australia, USA, Brazil, Israel, France, Italy, Turkey			
Cui 2019 [64] *Critically low*	**Serious AE** (15 RCTs)	TC (*n* = 476) vs physically active interventions (*n* = 489)	RD 0.0 (− 0.02 to 0.02) *Equivalent risk*	LOW *dd*
	**Non-serious AE** (15 RCTs)	TC (*n* = 476) vs physically active interventions (*n* = 489)	RD 0.01 (− 0.01 to 0.03) *Equivalent risk*	MODERATE *d*
	**TC related AE** (15 RCTs)	TC (*n* = 476) vs physically active interventions (*n* = 489)	RD 0.0 (− 0.01 to 0.02) *Equivalent risk*	MODERATE *d*
	**Serious AE** (9 RCTs)	TC (*n* = 421) vs physically inactive interventions (*n* = 408)	RD − 0.03 (− 0.06 to 0.00) *Equivalent risk*	MODERATE *d*
	**Non-serious AE** (9 RCTs)	TC (*n* = 421) vs physically inactive interventions (*n* = 408)	RD 0.03 (− 0.00 to 0.07) *Equivalent risk*	MODERATE *d*
	**TC related AE** (9 RCTs)	TC (*n* = 421) vs physically inactive interventions (*n* = 408)	RD 0.0 (− 0.01 to 0.02) *Equivalent risk*	MODERATE *d*
**General health and quality of Life**				
	**Older adults, with or without chronic diseases ***(also see cancer*,* cardiovascular diseases*,* diabetes*,* chronic obstructive pulmonary disease*,* Parkinson’s disease*,* perimenopause)*			
	Older adults; healthy, low bone mass, chronic obstructive pulmonary disease, chronic heart failure & depression, benign prostate hyperplasia, total knee arthroplasty, highly maladjusted institutionalized; in community settings, intermediate care rehabilitation unit, long-term care institution; China, Hong Kong, South Korea, USA, Spain, Germany, Iran			
Wang 2020 [91] *Critically low*	**QoL—overall** (6 RCTs)	TC (*n* = 277) vs Ucare, Ex (*n* = 275)	SMD 1.23 (0.56 to 1.89) *Large effect*	LOW *a*,* b*
	Older adults; with chronic disease—osteopenia, osteoporosis, osteoarthritis, stroke, hypertension, Parkinson’s disease, diabetes; in community settings; China, South Korea, USA, Australia, Turkey			
Choo 2020 [62] *Critically low*	**QoL—physical** (6 RCTs: 1 RCT Taylor-Piliae 2020 [90], 1 RCT Wang 2017 [92])	TC (*n* = 257) vs noRx, ADL, Ucare, HEd, attention control, waitlist (*n* = 238)	SMD 0.46 (0.13 to 0.80) *Small to moderate effect*	MODERATE *a*
	**QoL—mental health** (6 RCTs: 1 RCT Wang 2017 [92])	TC (*n* = 257) vs noRx, ADL, Ucare, HEd, attention control, waitlist (*n* = 238)	SMD 0.21 (0.03 to 0.39) *Small effect*	MODERATE *a*
	**Perimenopause**			
	Female adults and older adults; perimenopause, with or without low bone mineral density; in community settings; China, USA			
Wang 2017 [92] *Critically low*	**QoL—physical function** SF-36 (4 RCTs)	TC + / − placebo capsule (*n* = 154) vs Ucare, ADL, placebo capsule (*n* = 160)	MD − 1.8 points (− 5.2 to 1.6) *Equivalent effect*,* MCID* − *2 points* [50]	LOW *a*,* d*
	**QoL—bodily pain** SF-36 (3 RCTs)	TC + / − placebo capsule (*n* = 112) vs usual care, ADL, placebo capsule (*n* = 118)	MD − 3.6 points (− 6.6 to − 0.6) *Moderate effect*,* MCID* − *3 points *[50]	MODERATE *a* (†LOW)
	**QoL—general health** SF-36 (3 RCTs)	TC + / − placebo capsule (*n* = 112) vs ADL, placebo capsule (*n* = 118)	MD − 5.1 points (− 7.6 to − 2.6) *Large effect*,* MCID* − *2 points* [50]	MODERATE *a* (†LOW)
	**QoL—vitality** SF-36 (3 RCTs)	TC + / − placebo capsule (*n* = 112) vs ADL, placebo capsule (*n* = 118)	MD − 5.7 points (− 8.5 to − 2.8) *Large effect*,* MCID* − *2 points* [50]	MODERATE *a* (†LOW)
	**QoL—mental health** SF-36 (4 RCTs)	TC + / − placebo capsule (*n* = 154) vs Ucare, ADL, placebo capsule (*n* = 160)	MD − 2.5 (− 4.8 to − 0.2) *Small effect*,* MCID − 3 points* [50]	MODERATE *a* (†LOW)
	**QoL—social function** SF-36 (3 RCTs)	TC + /* − *placebo capsule (*n* = 112) vs ADL, placebo capsule (*n* = 118)	MD − 2.2 points (*− *5.0 to 0.6) *Equivalent effect*,* MCID − 3 points* [50]	LOW *a*,* d*
**Cancer**				
	Adults, older adults; cancer; in community settings; China, USA			
Ni 2019 [83] *Low* (†*Critically low*)	**QoL—physical**, breast cancer or female (9 RCTs)	TC (*n* = 331) vs Ucare, Rehab, HEd, Psych, Ex, sham Qigong (*n* = 348)	SMD 0.34 (0.09 to 0.59) *Small effect*	LOW *aa*
	**QoL—psychological**, breast cancer or female (9 RCTs)	TC (*n* = 333) vs Ucare, Rehab, HEd, Psych, Ex, sham Qigong (*n* = 348)	SMD 0.60 (0.12 to 1.08) *Moderate effect*	VERY LOW *aa*,* b*
	**QoL—social relationship**, breast cancer or female (8 RCTs)	TC (*n* = 292) vs Ucare, Rehab, HEd, Psych, Ex, sham Qigong (*n* = 303)	SMD 0.26 (0.25 to 0.77) *Small effect*	VERY LOW *aa*,* b*
	**Sleep quality**, breast or lung cancer (3 RCTs: 2 RCTs Si 2020 [86])	TC (*n* = 106) vs Ucare, Psych, sham Qigong (*n* = 112)	SMD 0.26 (*− *0.02 to 0.53) *Equivalent effect*	VERY LOW *aa*,* b*,* d*
	Adults, older adults; lung cancer, prostate cancer; in community settings; China			
Song 2018 [87] *Low* *(*†*Critically low)*	**Fatigue** < 8 weeks, lung cancer (2 RCTs)	TC (*n* = 77) vs Ucare, Ex (*n* = 74)	SMD − 0.5 (*− *0.83 to − 0.18) *Moderate effect*	VERY LOW *aa*,* d*
	**Fatigue** < 8 weeks, prostate cancer (1 RCT)	TC (*n* = 21) vs Ex (*n* = 45)	SMD 0.01 (*− *0.51 to 0.52) favours control *Equivalent effect*	VERY LOW *aa*,* dd*
	Adults, older adults; Breast cancer; in community settings; China, Thailand, USA			
Liu LZ 2020 [77] *Critically low*	**Fatigue** 3 months (2 RCTs)	TC + Ucare, Rehab (*n* = 60) vs Ucare, Rehab (*n* = 56)	SMD − 0.91 (*− *1.30 to − 0.53) *Large effect*	LOW *a*,* d*
	**Fatigue** 3 months (2 RCTs)	TC (*n* = 85) vs Psych, sham Qigong (*n* = 89)	MD − 0.46 points (*− *1.09 to 0.17) *Equivalent effect*,* MCID unknown*	LOW *a*,* d*
	**Fatigue** 6 months (2 RCTs)	TC (*n* = 80) vs Psych, sham Qigong (*n* = 83)	MD − 0.16 (*− *0.98 to 0.67) *Equivalent effect*,* MCID unknown*	LOW *a*,* d*
	Female adults; breast cancer; in community settings; China, Thailand, USA			
Luo 2020 [79] *Moderate(*†*Low)*	**Pain**, 3 weeks (2 RCTs)	TC (*n* = 110) vs Rehab (*n* = 109)	SMD 0.25 (*− *0.02 to 0.51) *Equivalent effect*	LOW *a*,* d*
	**Pain**, 3 months (4 RCTs)	TC (*n* = 169) vs Ucare, Rehab (*n* = 168)	SMD 0.30 (0.08 to 0.51) *Small effect*	MODERATE *a* (†LOW)
**Cardiovascular, diabetes, and risk factors**				
	**Chronic heart failure**			
	Adults, older adults; chronic heart failure, left ventricular ejection fraction (LVEF) ≤ 45%; in community settings; China, USA, UK, Italy			
Gu 2017 [66] *Low* *(*†*Critically low)*	**6-min walk test**—6-MWT (10 RCTs)	TC (*n* = 344) vs Ucare, HEd, Ex (*n* = 379)	MD 51 m (30.49 to 71.5) *Moderate effect*,* MCID 36 m *[57]	VERY LOW *aa*,* b*
	**Left ventricular ejection fraction**—LVEF (7 RCTs)	TC (*n* = 283) vs Ucare, HEd, Ex (*n* = 306)	MD 7.7% (3.6 to 11.9) *Moderate effect*,* MCID 3.2% *[53]	VERY LOW *aa*,* b*
	**QoL:** Minnesota Living with Heart Failure Questionnaire—MLHFQ (8 RCTs)	TC (*n* = 280) vs Ucare, HEd, Ex (*n* = 318)	MD − 10.4 points (*− *14.4 to − 6.3) *Moderate effect*,* MCID − 8 to − 19* [46]	VERY LOW *aa*,* b*
	Adults, older adults; chronic heart failure; in community settings; USA			
Taylor-Piliae 2020 [90] *Critically low*	**Psychological distress**, chronic heart failure (2 RCTs)	TC (*n* = 58) vs HEd, Ex (*n* = 58)	SMD − 0.58 (*− *0.95 to − 0.22) *Moderate effect*	MODERATE *d*
	**Ischaemic heart disease**			
	Older adults; stable angina; in community settings; China, Brazil			
Jiang 2018 [74] *Low(*†*Critically low)*	**VO**_**2**_**max** (4 RCTs)	TC (*n* = 148) vs noRx, Ex (*n* = 88)	SMD 2.2 (0.81 to 3.63) *Large effect*	VERY LOW *aa*,* b*
	Adults, older adults; myocardial infarction; in community settings; China			
Wu 2020 [96] *Low* *(*†*Critically low)*	**6-min walk time**—6MWT (5 RCTs)	TC (*n* = 234) vs Ucare, HEd, Ex (*n* = 231)	SMD 1.3 (0.50 to 2.11) *Large effect*	LOW *a*,* b*
	**Left ventricular ejection fraction**—LVEF (5 RCTs)	TC (*n* = 234) vs Ucare, HEd, Ex (*n* = 231)	SMD 1.0 (0.43 to 1.57) *Large effect*	LOW *a*,* b*
	**Hyperlipidaemia**			
	Adults, older adults; hyperlipidemia, type 2 diabetes mellitus, hypertension, obesity; in community settings; China, Hong Kong, Taiwan, Australia			
Pan 2016 [84] *Low* *(*†*Critically low)*	**Total cholesterol** (6 RCTs)	TC (*n* = 220) vs Ucare, Ex, waitlist (*n* = 225)	MD − 7.7 mg/dL (*− *17.3 to 1.4) *Equivalent effect*,* MCID 20 mg/dL* *(10% reduction from 200 mg/dL)*	VERY LOW *a*,* b*,* d*
	**Triglycerides** (6 RCTs)	TC (*n* = 220) vs Ucare, Ex, waitlist (*n* = 225)	MD − 16.8 mg/dL (*− *31.3 to − 2.4) *Moderate effect*,* MCID 15 mg/dL* *(10% reduction from 150 mg/dL)*	MODERATE *a*
	**High-density lipoprotein cholesterol**—HDL-C (5 RCTs)	TC (*n* = 192) vs Ucare, Ex (*n* = 200)	MD 0.46 mg/dL (*− *0.71 to 1.64) *Equivalenteffect*,* MCID 4 mg/dL* *(10% increase from 40 mg/dL)*	MODERATE *a* (†LOW)
	**Low-density lipoprotein cholesterol**—LDL-C (4 RCTs)	TC (*n* = 136) vs Ucare, Ex (*n* = 152)	MD − 1.61 mg/dL (*− *16.25 to 13.02) *Equivalent effect*,* MCIS − 10 mg/dL* *(10% reduction from 100 mg/dL)*	VERY LOW *a*,* bb*,* d*
	**Essential hypertension**			
	Adults, older adults; essential hypertension; in community settings; China, Taiwan			
Zhong 2020 [105] *High*	**Systolic blood pressure** (9 RCTs)	TC (*n* = 456) vs noRx, HEd (*n* = 458)	MD − 14.8 (*− *19.6 to − 10.0) *Large effect*,* MCID − 10 mmHg*	LOW *a*,* b*
	**Diastolic blood pressure** (9 RCTs)	TC (*n* = 456) vs noRx, HEd (*n* = 458)	MD − 7.0 (*− *9.1 to − 5.0) *Large effect*,* MCID − 5 mmHg*	MODERATE *a*
	**Systolic blood pressure** (15 RCTs)	TC (*n* = 406) vs Pharm (*n* = 348)	MD − 9.1 (*− *14.0 to − 4.1) *Moderate effect*,* MCID − 10 mmHg*	LOW *a*,* b*
	**Diastolic blood pressure** (15 RCTs)	TC (*n* = 406) vs Pharm (*n* = 348)	MD − 5.6 (*− *14.0 to − 4.1) *Moderate effect*,* MCID − 5 mmHg*	LOW *a*,* b*
	**Systolic blood pressure** (5 RCTs)	TC (*n* = 123) vs Ex (*n* = 123)	MD − 7.9 (*− *14.2 to − 1.7) *Small effect*,* MCID − 10 mmHg*	LOW *a*,* b* (†VERY LOW)
	**Diastolic blood pressure** (5 RCTs)	TC (*n* = 123) vs Ex (*n* = 123)	MD − 3.9 (*− *6.5 to − 1.2) *Small effect*,* MCID − 5 mmHg*	MODERATE *a* (†LOW)
	Adults, older adults; hypertension; in community settings; China, Hong Kong, USA			
Taylor-Piliae 2020 [90] *Critically low*	**QoL—mental health** (3 RCTs)	TC (*n* = 311) vs Ucare (*n* = 311)	SMD 0.13 (NI) *p* = 0.13 *Equivalent effect*	MODERATE *d*
	**QoL—physical** (3 RCTs)	TC (*n* = 311) vs Ucare (*n* = 311)	SMD 0.47 (NI) *p* < 0.001 *Small effect*	HIGH
	**Diabetes mellitus**			
	Adults, older adults; type 2 diabetes mellitus; in community settings; China, South Korea, Thailand, Australia			
Zhou 2019 [106] *Critically low*	**Glycosylated haemoglobin**—HbA1c % (14 RCTs)	TC (*n* = 466) vs Ucare, Ucare + TCM, HEd, sham exercise (*n* = 395)	MD − 0.88% (*− *1.45 to − 0.31) *Small effect*,* MCID 1%* [56]	LOW *a*,* b*
	**Systolic blood pressure**—SBP (5 RCTs)	TC (*n* = 151) vs Ucare, ADL, noEx (*n* = 139)	MD − 10.0 mmHg (*− *15.8 to − 4.3) *Moderate effect*,* MCID 10 mmHg*	MODERATE *a* (†LOW)
	**Diastolic blood pressure**—DBP (5 RCTs)	TC (*n* = 151) vs Ucare, ADL, noEx (*n* = 139)	MD − 4.9 mmHg (*− *8.2 to − 1.5) *Moderate effect*,* MCID 5 mmHg*	MODERATE *a* (†LOW)
	**QoL physical function** – SF36 (5 RCTs)	TC (*n* = 151) vs Ucare, ADL, noEx (*n* = 139)	MD 7.1 (0.79 to 13.4) *Large effect*,* MCID 3 points* [50]	LOW *a*,* b* (†VERY LOW)
	**QoL bodily pain** – SF36 (5 RCTs)	TC (*n* = 151) vs Ucare, ADL, noEx (*n* = 139)	MD 4.3 (0.8 to 7.8) *Moderate effect*,* MCID 3 points* [50]	MODERATE *a* (†LOW)
**Chronic obstructive pulmonary disease**				
	Adults, older adults; chronic obstructive pulmonary disease; in community settings; China, Hong Kong, USA			
Guo 2020 [67] *Low*	**Forced expiratory volume in 1 s**—FEV_1_, ≤ 3 months (3 RCTs)	TC (*n* = 111) vs noEx (*n* = 108)	MD 0.13L (0.06 to 0.20) *Moderate effect*,* MCID 0.1L* [43]	MODERATE *a* (†LOW)
	FEV_1_, ≤ 3 months (5 RCTs)	TC (*n* = 272) vs Ex + /or breathing Ex (*n* = 275)	MD 0.06L (*− *0.01 to 0.14) *Equivalent effect*,* MCID 0.1L* [43]	LOW *a*,* d*
	**6-min walk time**—6MWT, ≤ 3 months (6 RCTs)	TC (*n* = 182) vs noEx (*n* = 181)	MD 24.3 m (6.3 to 42.3) *Small effect*,* MCID 30–80 m* [55, 59]	LOW *a*,* b* (†VERY LOW)
	6MWT, ≤ 3 months (6 RCTs)	TC (*n* = 308) vs Ex + /or breathing Ex (*n* = 313)	MD 7.5 m (2.1 to 12.3) *Very small effect*,* MCID 30–80 m* [55, 59]	MODERATE *a*
	**QoL—St George Respiratory Questionnaire**—SGRQ, ≤ 3 months (3 RCTs)	TC (*n* = 129) vs noEx (*n* = 128)	MD − 8.7 points (*− *14.6 to − 2.7) *Large effect*,* MCID − 2.8 to − 7.6 points* [40, 49]	MODERATE *a* (†LOW)
	**QoL—**SGRQ, ≤ 3 months (4 RCTs)	TC (*n* = 260) v Ex + /or breathing Ex (*n* = 265)	MD − 1.9 points (*− *4.6 to 0.7) *Equivalent effect*,* MCID − 2.8 to − 7.6 points* [40, 49]	MODERATE *a*
**Cognitive function and impairment**				
	Older adults; no cognitive impairment; in in community settings; China, Hong Kong, Japan, France			
Wayne 2014 [94] *Critically low*	**Executive function** (4 RCTs)	TC (*n* = 151) vs noEx (*n* = 270)	SMD 0.90 (0.03 to 1.78) *Large effect*	MODERATE *b*
	**Executive function** (2 RCTs)	TC (*n* = 67) vs Ex (*n* = 69)	SMD 0.51 (0.17 to 0.85) *Moderate effect*	MODERATE *d*
	Older adults; Mild cognitive impairment; in community settings; China, Thailand, USA, France			
Zhang 2020 [102] *Low* *(*†*Critically low)*	**Global cognitive function**—Mini-Mental State Examination—MMSE (5 RCTs)	TC (*n* = 325) vs Cognition-action, Ucare, HEd, Ex, other activities (*n* = 460)	MD 0.29 points (*− *0.61 to 0.74) *Equivalent effect*,* MCID 1 point* [41]	HIGH
	**Memory**—Delayed Recall Test (4 RCTs)	TC (*n* = 297) vs ADL, HEd, Ex (*n* = 429)	MD 0.37 points (0.13 to 0.61) *A positive effect*,* MCID unknown*	HIGH
	**Performance**—Digit Span Test (4 RCTs)	TC (*n* = 297) vs ADL, HEd, Ex (*n* = 429)	MD 0.03 point (*− *0.16 to 0.22) *Equivalent effect*,* MCID unknown*	HIGH
**Fatigue, fibromyalgia, and sleep quality**				
	**Fatigue, any cause**			
	Adults, older adults; fatigue without serious ailments, cancer, multiple sclerosis, chronic obstructive pulmonary disease, insomnia, rheumatoid arthritis; in community settings; China, Hong Kong, USA, Spain, Germany			
Xiang 2017 [97] *Moderate* *(*†*Low)*	**Fatigue** (10 RCTs)	TC (*n* = 356) vs noRx, Ucare, HEd, Ex, sham Qigong (*n* = 333)	SMD − 0.45 (*− *0.70 to − 0.20) *Small effect*	MODERATE *a*
	**Vitality** (4 RCTs)	TC (*n* = 115) vs noRx, HEd, Ex (*n* = 333)	SMD 0.63 (0.20 to 1.07) *Moderate effect*	LOW *aa*
	**Depression** (7 RCTs)	TC (*n* = 216) vs noRx, Ucare, HEd, Ex, other control (*n* = 199)	SMD − 0.58 (*− *1.04 to − 0.11) *Moderate effect*	VERY LOW *aa*,* b*
	**Fibromyalgia**			
	Adults; fibromyalgia; in community settings; USA, South Korea, UK, Italy			
Cheng 2019 [61] *Low* *(*†*Critically low)*	**QoL—Fibromyalgia impact questionnaire**—FIQ 12–16 weeks (4 RCTs)	TC (*n* = 158) vs Ucare (*n* = 149)	SMD − 0.61 (*− *0.90 to − 0.31) *Moderate effect*	MODERATE *a* (†LOW)
	**QoL—**FIQ 24–32 weeks (2 RCTs)	TC (*n* = 82) vs Ucare (*n* = 78)	SMD − 0.49 (*− *1.56 to 0.58) *Equivalent effect*	VERY LOW *a*,* b*,* dd*
	**Pain** (3 RCTs)	TC (*n* = 100) vs noRx, Ucare, HEd, Ex (*n* = 90)	SMD − 0.88 (*− *1.58 to − 0.18) *Large effect*	VERY LOW *a*,* b*,* d*
	**Sleep quality**			
	Adults, older adults; healthy, stroke, fibromyalgia, cancer, arthritis, depression, chronic kidney disease, heart disease; in community settings; China, Japan, Vietnam, USA, Italy, Iran			
Si 2020 [86] *Low* *(*†*Critically low)*	**Pittsburgh Sleep Quality Index**, healthy (10 RCTs)	TC (*n* = 426) vs noRx, Ex, HEd (*n* = 401)	SMD − 0.68 (*− *1.06 to − 0.31) *Moderate effect*	LOW *a*,* b*
	**Pittsburgh Sleep Quality Index**, chronic disease (15 RCTs)	TC (*n* = 543) vs Ucare, Hed, Psych, Rehab, sham Qigong, acupuncture, waitlist (*n* = 564)	SMD − 0.39 (*− *0.74 to − 0.05) *Small effect*	LOW *a*,* b*
**Mental health**				
	**Depression, anxiety, stress, mood for general populations ***(also see chronic heart failure*,* stroke*,* knee osteoarthritis*,* fatigue)*			
	Adults, older adults; depression and/or chronic diseases; Asian, North American and European countries			
Yin 2014 [98] *Low*	**Depression** (25 RCTs: 1RCT Lyu 2020 [80], 1 RCT Hu 2020 [70])	TC vs noRx, Ex, sham/other (total sample < 1435)	SMD 0.36 (0.19 to 0.53) *Small effect*	HIGH
	**Anxiety** (11 RCTs)	TC vs noRx (total sample < 1435)	SMD 0.34 (0.02 to 0.66) *Small effect*	MODERATE *b*
	Adults, older adults; healthy, osteoarthritis, rheumatoid arthritis, fibromyalgia, HIV infection, depression, frail; in community settings; China, USA, Australia, UK, Germany, France			
Wang 2010 [93] *Critically low*	**Stress** (4 RCTs)	TC vs ADL, Psych, waitlist (total sample *n* = 308)	SMD 0.97 (0.06 to 1.87) *Large effect*	VERY LOW *aa*,* bb*
	**Mood / affect** (2 RCTs)	TC vs ADL, Psych, waitlist (total sample *n* = 191)	SMD 0.25 (*− *0.04 to 0.53) *Equivalent effect*	VERY LOW *aa*,* d*
	**Schizophrenia**			
	Adults, older adults; schizophrenia; in hospital, long-stay care, halfway house service; China			
Zheng 2016 [104] *Moderate* (†*Low*)	**Negative symptoms**—Positive and Negative Syndrome Scale—PANSS (6 RCTs)	TC + Ucare (*n* = 200) vs Ucare + /* − *Pharm, HEd, Ex, noEx, waitlist (*n* = 251)	SMD − 0.87 (*− *1.51 to − 0.24) *Large effect*	LOW *a*,* b*
	**Positive symptoms**—PANSS (5 RCTs)	TC + Ucare (*n* = 170) vs Ucare + /* − *Pharm, HEd, Ex, noEx, waitlist (*n* = 221)	SMD − 0.09 (*− *0.44 to 0.26) *Equivalent effect*	MODERATE *a* (†LOW)
	**Discontinuation** rate (4 RCTs)	TC + Ucare (*n* = 170) vs Ucare + /* − *Pharm, HEd, Ex, noEx, waitlist (*n* = 221)	RR 0.06 (0.23 to 1.40) 3 fewer per 100 adults	VERY LOW *a*,* dd*
**Musculoskeletal conditions and pain**				
	**Osteoarthritis**			
	Older adults; knee osteoarthritis; in community settings; China, South Korea, USA			
Hu 2020 [70] *Low* *(*†*Critically low)*	**WOMAC pain** (14 RCTs)	TC (*n* = 455) vs Ucare, noEx, HEd, PT (*n* = 422)	SMD − 0.69 (*− *0.95 to − 0.44) *Moderate effect*	MODERATE *a*
	**WOMAC stiffness** (12 RCTs)	TC (*n* = 396) vs Ucare, noEx, HEd, PT (*n* = 373)	SMD − 0.65 (*− *0.98 to − 0.33) *Moderate effect*	LOW *a*,* b*
	**WOMAC physical function** (13 RCTs)	TC (*n* = 437) vs Ucare, noEx, HEd, PT (*n* = 407)	SMD − 0.92 (*− *1.16 to − 0.69) *Large effect*	MODERATE *a*
	**Depression** (3 RCTs: 1 RCT in Yin 2014 [98])	TC (*n* = 167) vs Ucare, noEx, HEd, PT (*n* = 152)	SMD − 0.46 (*− *0.68, − 0.24) *Small effect*	MODERATE *a*
	**Arthritis self-efficacy** scale (4 RCTs)	TC (*n* = 185) vs Ucare, noEx, HEd, PT (*n* = 167)	SMD 0.27 (0.06 to 0.48) *Small effect*	MODERATE *a* (†LOW)
	Adults, older adults; healthy, osteoarthritis; in in community settings			
Su 2020 [88] *Critically low*	**Knee extensor muscle strength**, females (60°/s) (2 RCTs)	TC (*n* = 40) vs noRx, Ex, Pharm, HEd (*n* = 45)	MD 17.5 (*− *12.0 to 47.0) *Equivalent effect*,* MCID unknown*	VERY LOW *a*,* b*,* dd*
	**Knee flexor muscle strength**, females (60°/s) (2 RCTs)	TC (*n* = 40) vs noRx, Ex, Pharm, HEd (*n* = 45)	MD 22.1 (1.1 to 43.2) *Positive effect*,* MCID unknown*	VERY LOW *a*,* dd* (†LOW)
	**Knee flexor muscle strength** one maximum strength—1-RM (2 RCTs)	TC (*n* = 57) vs noRx, HEd (*n* = 57)	MD 3.3 (2.1 to 4.4) *Positive effect*,* MCID unknown*	LOW *a*,* d*
	**Knee extensor muscle strength** 1-RM (4 RCTs)	TC (*n* = 114) vs noRx, HEd, Ex (*n* = 112)	SMD 0.90 (0.34 to 1.45) *Large effect*	MODERATE *a* (†LOW)
	**Rheumatoid arthritis**			
	Adults, older adults; rheumatoid arthritis; in community settings; China, South Korea, USA			
Mudano 2019 [82] *High*	**Pain**, visual analogue scale, 12 weeks (2 RCTs)	TC (*n* = 42) vs noEx, Ex (*n* = 39)	SMD − 0.95 (*− *1.41 to − 0.49) *Large effect*	VERY LOW *aa*,* dd*
	**Disease activity**, DAS-28-ESR, 12 weeks (1 RCT)	TC (*n* = 29) vs HEd (*n* = 14)	MD − 0.40 points (*− *1.10 to 0.30) *Equivalent effect*,* MCID − *1.17 *points* [58]	VERY LOW *aa*,* dd*
	**Function**, Health Assessment Questionnaire – HAQ, 12 weeks (2 RCTs)	TC (*n* = 39) vs Hed, Ex (*n* = 24)	MD − 0.33 points (*− *0.79 to 0.12) *Equivalent effect*,* MCID − 0.38 points* [58]	VERY LOW *aa*,* b*,* dd*
	**Low back pain**			
	Adults, older adults; Low back pain; in community settings; China, Australia			
Qin 2019 [85] *Low* (†*Critically low*)	**Pain** VAS 1–10 scale (3 RCTs)	TC (*n* = 123) vs ADL, waitlist (*n* = 120)	MD − 1.2 points (*− *2.3 to − 1.1) *Moderate effect*,* MCID − 1.2*	LOW *a*,* b* (†VERY LOW)
	**Pain** VAS 1–10 scale (5 RCTs)	TC + Ucare (*n* = 363) vs Ucare (*n* = 268)	MD − 1.1 (*− *1.3 to − 0.9) *Moderate effect*,* MCID − 1.2*	MODERATE *a*
	**Headache**			
	Adults, older adults; chronic pain from tension headaches; in community settings; USA			
Hall 2017 [68] *Low* *(*†*Critically low)*	**Pain** SF-36 15 weeks (1 RCT)	TC (*n* = 13) vs waitlist (*n* = 17)	SMD − 1.85 (*− *2.73 to − 0.97) *Large effect*	VERY LOW *aa*,* dd*
	**Osteoporosis, osteopenia**			
	Adults, older adults; osteoporosis, osteopenia; in community settings; NI countries			
Zhang 2019 [101] *Moderate* (†*Low*)	**Spine** **Bone mineral density**—BMD (6 RCTs)	TC (*n* = 128) vs noRx (*n* = 119)	MD 0.04 g/cm^2^ (0.02 to 0.06) *Small effect*,* MCID* ~ *0.05 g/cm*^*2*^ [54]	MODERATE *a* (†LOW)
	**Femur BMD** (3 RCTs)	TC (*n* = 85) vs noRx (*n* = 83)	MD 0.04 g/cm^2^ (0.01 to 0.06) *Small effect*,* MCID* ~ *0.05 g/cm*^*2*^ [54]	LOW *a*,* d*
	**Spine BMD** (2 RCTs)	TC (*n* = 52) vs Ucare (*n* = 55)	MD 0.16 g/cm^2^ (0.09 to 0.23) *Large effect*,* MCID* ~ *0.05 g/cm*^*2*^ [54]	LOW *a*,* d*
	**Femur BMD** (2 RCTs)	TC (*n* = 52) vs Ucare (*n* = 55)	MD 0.16 g/cm^2^ (0.04 to 0.29) *Large effect*,* MCID* ~ *0.05 g/cm*^*2*^ [54]	VERY LOW *a*,* b*,* d*
**Stroke, Parkinson’s disease, and falls**				
	**Stroke**			
	Adults, older adults; healthy, type 2 diabetes mellitus, hyperlipidaemia; in community settings; China			
Zheng 2015 [103] *Low* (†*Critically low*)	**Incidence of nonfatal stroke** over 1–2 years (2 RCTs)	TC + Ucare (*n* = 62) vs Ucare (*n* = 58)	RR 0.11 (0.01 to 0.85) *89% reduced risk*	LOW *a*,* d*
	**Incidence of fatal stroke** over 1–2 years (2 RCTs)	TC + Ucare (*n* = 62) vs Ucare (*n* = 58)	RR 0.33 (0.05 to 2.05) *77% reduced risk*	LOW *a*,* d* (†VERY LOW)
	Adults, older adults; stroke survivors; in community settings; NI countries			
Lyu 2018[81] *Moderate* *(*†*Low)*	**Berg Balance Scale**—BBS (2 RCTs)	TC (*n* = 75) vs Rehab (*n* = 75)	MD 5.2 points (3.4 to 7.1) *Moderate effect*,* MCID 4.3 to 7.3 points* [47]	LOW *a*,* d*
	**Fugl-Meyer Assessment FMA—all four limbs** (2 RCTs)	TC + Rehab (*n* = 51) vs Rehab (*n* = 49)	MD 4.5 points (1.9 to 7.1) *A positive effect*,* MCID unknown*	LOW *a*,* d*
	**FMA—upper extremity** (2 RCTs)	TC + Rehab (*n* = 56) vs Rehab (*n* = 51)	MD 8.3 points (4.7 to 11.8) *Large effect*,* MCID 5.3* points [51]	LOW *a*,* d*
	**FMA—lower extremity** (3 RCTs)	TC + Rehab (*n* = 85) vs Rehab (*n* = 81)	MD 2.8 points (0.95 to 4.56) *Small effect*,* MCID 6* points [52]	VERY LOW *a*,* b*,* d*
	**Timed up and go**—TUG (4 RCTs)	TC + Rehab (*n* = 100) vs Rehab (*n* = 96)	MD 2.6 s (1.8 to 3.4) *Small effect*,* MCID 8 s* [47]	LOW *a*,* d*
	**Activities of daily living**—Barthel Index (2 RCTs)	TC (*n* = 81) vs Rehab (*n* = 85)	MD 9.9 points (6.8 to 13.0) *Large effect*,* MCID 6.8 points* [44]	LOW *a*,* d*
	Adults, older adults; stroke survivors; in community settings; China, South Korea, Japan, USA, Israel			
Lyu 2020 [80] *Moderate* (†*Low)*	**Depression** (6 RCTs)	TC (*n* = 278) vs Rehab (*n* = 280)	SMD 0.36 (0.10 to 0.61) *Small effect*	LOW *aa*
	**Parkinson’s disease**			
	Older adults; Parkinson’s disease; in community settings; NI countries			
Yu 2018 [100] *Critically low*	**Unified Parkinson’s Disease Rating III: Motor** (8 RCTs)	TC (*n* = 204) vs noRx, Ucare, Pharm, Ex (*n* = 262)	MD − 3.7 points (*− *5.7 to − 1.7) *Moderate effect*,* MCID − 3.3 points* [48]	MODERATE *b*
	**Timed up and go**—TUG (7 RCTs)	TC (*n* = 188) vs noRx, Ucare, Pharm, Ex (*n* = 251)	SMD − 0.50 (*− *0.88 to − 0.11) *Moderate effect*	HIGH
	**Berg balance scale**—BBS (6 RCTs)	TC (*n* = 144) vs noRx, Ucare, Pharm, Ex (*n* = 145)	SMD 0.85 (0.44 to 1.27) *Large effect*	HIGH (†MODERATE)
	**QoL – Parkinson’s Disease Questionnaire**—PDQ-39, PDQ-8 (3 RCTs)	TC (*n* = 104) vs noRx, Ucare, Pharm, Ex (*n* = 159)	SMD − 0.75 (*− *1.45 to − 0.04) *Moderate effect*	HIGH (†MODERATE)
	**Falls and risk factors**			
	Older adults; with or without a history of falling, stroke, Parkinson’s disease, females with osteopenia; in hospital, in community settings; China, Taiwan, USA, Canada, Australia, New Zealand, Netherlands			
Huang 2017 [73] *Low* *(*†*Critically low)*	**Rate of people who fell** (no. of fallers) (16 RCTs)	TC (*n* = 1889) vs ADL, noRx, PT, Ex (*n* = 1650)	RR 0.80 (0.72 to 0.88) *20% reduced risk*,* 9 fewer per 100*	MODERATE *e*
	**Incidence of falls** (no. falls) (15 RCTs)	TC (*n* = 1512) vs ADL, noRx, PT, Ex (*n* = 1542)	RR 0.69 (0.60 to 0.80) *31% reduced risk*	MODERATE *e*
	Older adults; in hospital, nursing home, in community settings; China, USA, Canada, Australia, New Zealand, UK, Netherlands			
Huang 2020 [71] *Critically low*	**Balance** – Single Leg Stance (SLS) (8 RCTs)	TC (*n* = 417) vs ADL, Ex, other activities (*n* = 419)	MD 5.8 s (0.62 to 10.90) *Small effect*,* MCID 41 s *[45]	VERY LOW *a*,* bb*
	**Berg balance scale**—BBS (4 RCTs)	TC (*n* = 412) vs ADL, Ex (*n* = 400)	MD 1.0 points (0.2 to 1.9) *Small effect*,* MCID 4 points *[42]	MODERATE *a*
	**Timed up and go**—TUG (6 RCTs)	TC (*n* = 190) vs ADL, Ex (*n* = 178)	MD − 0.71 s (*− *0.88 to − 0.54) *Probably small effect*,* MCID unknown*	MODERATE *a* (†LOW)
	Older adults; with or without a history of falling; in community settings; USA, Canada, China, Vietnam, Iran			
Kruisbrink 2020 [75] *Low* *(*†*Critically low)*	**Fear of falling** (6 RCTs)	^§^TC with an instructor vs TC with no information about instructor (NI sample size)	SMD.B − 1.05 (*− *1.60 to − 0.50) *Large effect*	VERY LOW *aa*,* b*,* e*

^*§*^*Erratum published 3 Sept. 2022 confirming control group was also TC. CI* confidence interval, *RD* risk difference, *MD* mean difference, *MID* minimally important difference, *SMD* standardized mean difference, *SMD.B: regression co-efficient for standardised mean difference, RR* relative risk, *RCT* randomized controlled trial, *QoL* quality of life, *ADL* routine activities of daily living/ routine lifestyle, *Ex* exercise (any type, including stretching), *HEd* health/lifestyle/other education, *noRx* no treatment, control, *Pharm* pharmaceutical drugs / medication, *Psych* psychological interventions, counselling, support, *PT* physical therapy/physiotherapy, *Rehab* rehabilitation programs, *TC* Tai Chi intervention, *TCM* traditional Chinese herbal medicine, *Ucare* usual care, conventional treatment, standard medical care, *MCID* minimal clinically important difference, for SMD ≥ 0.50 is a moderate effect and SMD ≥ 0.80 large effect, *a* serious risk of bias, *aa* very serious risk of bias, *b* serious inconsistency between studies, *bb* very serious inconsistency between studies, *c* serious indirectness of evidence, *cc* very serious indirectness of evidence, *d* serious imprecision of effect, *dd* very serious imprecision of effect, *e* serious publication bias, *ee* very serious publication bias

^*^Estimate of effect favours Tai Chi unless stated otherwise

†Sensitivity analysis suggests a different rating.

For AMSTAR-2 refer to Additional File 5. For GRADE certainty refer to Additional File 6

### GRADE evidence certainty

Of the 114 estimates of effect that were extracted, only eight (7.0%) were graded as high certainty evidence; 43 (37.7%) moderate, 36 (31.6%) low, and 27 (23.7%) very low. Serious or very serious concerns with the risk of bias of the individual RCTs was the predominant issue that negatively impacted 92 (80.7%) of the extracted effect estimates. Imprecision in effect estimates was the next most common issue (43 effect estimates, 37.7%) that was a function of the small number of studies in the meta-analysis and/or their small sample sizes. Thirty-seven (32.5%) effect estimates were graded down for inconsistency. Whilst all the meta-analyses had at least one RCT with a small sample size, only three instances of publication bias were identified. However, if the thresholds and criteria from the post hoc sensitivity analyses were applied, then 31 (25.8%) estimates would be further downrated due to serious or very serious concerns with imprecision, and 6 (5.0%) estimates would be rated up from very serious to serious concerns. In this instance, only 6 (5.0%) would be graded as high certainty evidence; 28 (23.3%) moderate, 53 (44.2%) low, and 33 (27.5%) very low. Details of the GRADE certainty assessments can be found in Additional file 6.

### Summary of the effects of Tai Chi

Table 3 presents the Summary of Findings of 114 estimates of effect and the GRADE certainty of the evidence of Tai Chi SRs according to population, outcome, and comparison that were extracted from 37 SRs with a meta-analysis. Of the 108 estimates of effect reported for Tai Chi treatment outcomes, 107 favoured Tai Chi. However, 21 estimates were not significant and are interpreted as equivalent to the comparison groups. This included the one estimate that favoured the comparison groups.

#### Adverse events

Cui et al. [64] evaluated the overall safety of Tai Chi. No significant differences were found in the risk of serious, non-serious, or intervention-related adverse events (AEs) from Tai Chi compared to both physically active and inactive interventions in healthy adults and people with chronic diseases (low to moderate certainty). The most common AEs were non-serious AEs, such as musculoskeletal aches and pains. Serious AEs were found in studies involving patients with heart failure, including death, hospitalized, and worsening heart failure or its co-morbidities. The reviewers reported that no serious AEs were determined to be attributable to Tai Chi or control conditions. The reviewers noted that an important limitation of the evidence was ongoing underreporting of AEs in many RCTs and only a few used an AE monitoring protocol.

Twenty of the other SRs included in the evidence synthesis also reported AEs (Table 2). Of which, 18 reported no AEs [62, 70, 72, 76, 81, 83, 84, 86, 89, 90, 93, 94, 96–98, 103, 104] and two reported mild, transient musculoskeletal AEs [82, 85].

#### General health, quality of life, and wellbeing

Whilst most SRs were for adults and older adults with chronic diseases, a SR with no meta-analysis reported various physical and psychological benefits of Tai Chi for students in higher education [95]. Another SR with no meta-analysis reported improved workplace productivity/motivation and work-related stress for healthcare workers [63].

Health-related quality of life (QoL) outcomes were frequently evaluated for adults and older adults, most of whom had one or more chronic diseases. The results from the meta-analyses of QoL outcomes for single conditions are presented in their respective sections below. Disease-specific QoL outcomes are reported for chronic heart failure [66], chronic obstructive pulmonary disease [67], fibromyalgia [61], and Parkinson’s disease [100], and generic QoL outcomes for cancer [83], hypertension [90], and type 2 diabetes mellitus [106]. Other related outcomes are reported for stroke (activities of daily living) [81], rheumatoid arthritis (functional status), and knee osteoarthritis (self-efficacy) [70].

Three additional SRs representing QoL outcomes for other populations were also selected. For women in the perimenopausal life stage, there was moderate certainty evidence of a clinically important effect for some of the Short Form Health Survey 36-item (SF-36) QoL domains (general health, vitality, bodily pain, and mental health) and low certainty evidence of equivalence to other control groups for the physical and social function QoL domains [92]. For older adults with or without chronic diseases, there were clinically important improvements in overall QoL that was measured using various generic and disease-specific QoL tools (low certainty) [91]. For those with chronic diseases, there were small improvements in both the physical and mental health SF-36/SF-12 QoL domains (moderate certainty) [62]. For the physical QoL domain, two RCTs overlapped with other reported effect estimates, one for hypertension (high certainty, small effect) [90] and one for perimenopause (low certainty, equivalent effect) [92], and for the mental health domain, one RCT overlapped with the perimenopause effect estimate (moderate certainty, small effect) [92].

#### Cancer

The effects of Tai Chi on QoL, pain, fatigue, and sleep were commonly appraised, particularly for breast cancer survivors. Four SRs were selected [77, 79, 83, 87]; however, none of the SRs were comprehensive and all of them had missed numerous eligible RCTs. Most of the effects from Tai Chi were either small or equivalent to the comparison groups, or there was very low certainty evidence.

For female cancer survivors, the evidence was more mixed. There was low certainty evidence of small improvements in the QoL physical domain [83]. However, the effects of Tai Chi were unclear for both psychological and social QoL domains due to very low certainty evidence [83]. For breast cancer survivors only, there were clinically important improvement in fatigue at 3 months when Tai Chi was added to usual care or rehabilitation (low certainty) [77], yet no difference at 3 or 6 months compared to psychological interventions or sham Qigong (low certainty evidence) [77]. Similarly, compared to usual care or rehabilitation, there were small improvements in pain at 3 months (moderate certainty), yet no difference at 3 weeks compared to rehabilitation only (low certainty) [79].

#### Cardiovascular diseases, diabetes, and risk factors

For adults and older adults post myocardial infarction, clinically important improvements in VO_2_-max were found (low certainty) [96], but the effects were unclear for older adults with stable angina due to very low certainty evidence [74]. For those with chronic heart failure, the effects of Tai Chi on left ventricular ejection fraction (LVEF), distance they could walk in 6 min, and disease-specific QoL were also unclear due to very low certainty evidence [66]. However, there was moderate certainty of clinically important improvements in psychological distress for people with chronic heart failure [90].

Clinically important reductions in both systolic and diastolic blood pressure were found for people with essential hypertension (moderate to low certainty) [102] and diabetes mellitus (moderate certainty) [106]. There was probably no effect for normotensive adults; however, the estimates are not reported because some RCTs were excluded from the final the meta-analyses and no sensitivity analysis was reported [65]. The antihypertensive effects for people with essential hypertension were greatest when Tai Chi was compared to no intervention or health education (moderate to low certainty, large effect), followed by anti-hypertensive medication (low certainty, moderate effect), and then other exercise interventions (moderate to low certainty, small effect) [102]. Compared to usual care, the effects of Tai Chi on psychological QoL were equivalent (moderate certainty) and there were small improvements in physical QoL (high certainty) [90].

The effects of Tai Chi were mixed for people with hyperlipidemia. Only moderate reductions in triglyceride levels were found (moderate certainty), and there was probably no difference between Tai Chi and usual care or other types of exercise on total cholesterol, high-density lipoprotein cholesterol, or low-density lipoprotein cholesterol (low to very low certainty) [84].

For people with type 2 diabetes mellitus, improvements in glycemic control were small and unlikely to be clinically important (moderate certainty) [106]. However, there were clinically important improvements in the QoL domains of pain and physical function (moderate certainty) [106].

#### Chronic obstructive pulmonary disease

When Tai Chi was compared to no exercise controls for people with chronic obstructive pulmonary disease, there were clinically important improvements in both lung function and disease-specific QoL (moderate certainty); however, the improvement in the distance walked in 6 min was unlikely to be clinically important (low certainty) [67]. Tai Chi was unlikely to be any more effective than other types of exercise (moderate to low certainty) [67].

#### Cognitive function and impairment

Clinically important effects on the executive function of people with no cognitive impairment were found when Tai Chi was compared to no exercise and exercise (moderate certainty) [94]. For people with mild cognitive impairment, only the delayed recall test improved (high certainty) [102]. There were no differences between the Tai Chi and control groups’ mini-mental state examination (MMSE) (high certainty) and digit span tests (moderate certainty) [102].

#### Fatigue, sleep quality, and fibromyalgia

For adults suffering from fatigue, with or without any serious ailments or chronic diseases, there were clinically important improvements in vitality (low certainty) and small improvements in fatigue (moderate certainty) [97].

For healthy adults, there were moderate improvements in sleep quality (low certainty) and small improvements for adults with chronic diseases (low certainty) [86]. Two of the three RCTs in the meta-analysis of sleep quality for cancer survivors (very low certainty, equivalent effect) [83] overlapped with the this larger meta-analysis of 15 RCTs for adults with chronic diseases [86].

For adults with fibromyalgia, there were clinically important improvements in activities of daily living after 12 to 16 weeks when Tai Chi was compared to usual care (moderate certainty); however, at 24 to 32 weeks, the effects were unclear due to very low certainty evidence [61]. Whether Tai Chi reduced the pain from fibromyalgia was also unclear due to very low certainty evidence [61].

#### Immunity

One SR with no meta-analysis reported improvements in cell-mediated immunity (including in people with HIV infections) and antibody levels (including in older adults) [69]. However, none of the studies included in the SR evaluated whether these improvements translated into direct clinical outcomes such as preventing or recovering from infections.

#### Mental health

Except for schizophrenia and university students with symptoms of depression, the SRs pooled the results of studies of participants who had mental health problems such as depression with studies of participants who had other health conditions in which mental health problems are a common comorbidity.

For adults and older adults with chronic diseases, including those suffering from depression, a 2014 SR reported small improvements in depression outcomes (high quality) and anxiety outcomes (moderate quality) and both estimates of effect were stable after adjusting for participants’ severity of baseline symptoms, health status, age, and ethnicity, and whether depression or anxiety was the primary outcome of the RCT [98]. The findings were congruent with more recent SRs that reported depression outcomes for stroke survivors (low certainty, small effect, one overlapping RCT) [80], fatigue from any cause (very low certainty, moderate effect, no overlapping RCTs) [97], knee osteoarthritis (moderate certainty, small effect, one overlapping RCT) [40] and older adults (moderate certainty, small effect, three overlapping RCTs) [62], and also psychological distress associated with chronic heart failure (moderate certainty, moderate effect, no overlapping RCTs) [90]. However, in another SR with no overlapping RCTs, due to very low certainty evidence, it was unclear if stress or mood outcomes improved in those with chronic diseases [93].

Improvements in depression outcomes were found when university students with depression or depressive symptoms used Tai Chi compared to no intervention or other exercise; however, the effect estimate was not extracted due to a probable data transformation error [99].

Clinically important improvements in negative symptoms (low certainty), but not positive symptoms (moderate certainty) of schizophrenia, were found when Tai Chi was added to usual care; however, it was unclear if discontinuation rates were lower (very low certainty) [104].

#### Multiple sclerosis

A SR with no meta-analysis reported positive improvements in fatigue, as well as balance, gait, flexibility, depression, and quality of life in adults with multiple sclerosis [89]. However, despite this positive trend, in a subgroup analysis of fatigue for any condition, the findings from two RCTs (one overlapping) were not significant (SMD − 0.77, 95% CI − 1.76 to 0.22) [97].

#### Musculoskeletal conditions and pain

Most of the SRs and their included primary studies were for older adults with knee osteoarthritis. There were clinically important improvements in pain (moderate certainty), stiffness (low certainty), physical function (moderate certainty), and depression outcomes (moderate certainty), as well as small improvements in self-efficacy (moderate certainty) [70]. Similar findings were also reported in the most recent SR for any type of osteoarthritis [78]. However, the effect estimates were not extracted due probable data transformation errors and/or extensive overlap with the meta-analyses reported for knee osteoarthritis.

The effects of Tai Chi on knee flexor and extensor muscle strength were also evaluated in adults with or without osteoarthritis. The effects favoured Tai Chi, especially when Tai Chi was only compared to non-exercise controls (low or moderate certainty) [88].

For people with rheumatoid arthritis, whilst the results were promising, there was only very low certainty evidence about the effects of Tai Chi on pain, disease activity, and function [82].

The findings were mixed for people with osteoporosis or osteopenia. Compared to usual care, there were clinically important improvements in spine bone mineral density (BMD) (low certainty) and possibly femur BMD (very low certainty) [101]. Compared to no-treatment controls, the improvements in spine BMD (moderate certainty) and femur BMD (low certainty) were small and probably clinically unimportant [101].

Regarding pain outcomes, there were clinically important improvements in bodily pain for perimenopausal females with or without osteopenia/osteoporosis (moderate certainty) [92] and low back pain when compared to usual care (moderate certainty) or inactive controls (low certainty) [85]. However, due to very low certainty evidence, it was unclear if Tai Chi reduced pain caused by tension headaches [68]. No SRs were identified that synthesized results for neck or shoulder pain.

#### Stroke, Parkinson’s disease, and falls

There was low certainty evidence of a 77% reduced risk of fatal stroke and an 89% reduction in the risk of nonfatal stroke over 1 to 2 years, in healthy older adults and people with diabetes and/or hyperlipidemia [103]. For stroke survivors, the addition of Tai Chi to their rehabilitation program resulted in clinically important improvements in upper limb function (low certainty) and balance (low certainty). The effects on lower limb function were unclear due to very low certainty evidence and there were only small improvements in timed up-and-go tests (low certainty) [81]. Compared to rehabilitation, there was low certainty evidence of improvements in disease-specific activities of daily living [81] and depression outcomes [80]. However, the improvements in depression were small and unlikely to be clinically important.

Clinically important improvements in the overall motor function of people with Parkinson’s disease (moderate certainty), balance (high certainty), and timed up-and-go tests (high certainty), as well as their disease-specific QoL (high certainty), were found [100].

Falls prevention and associated risk factors such as balance, mobility, and fear of falling were commonly reviewed. Tai Chi was found to reduce the risk of falling by at least 20% (NNT: 11) for older adults with or without a history of falling, including adults with Parkinson’s disease and stroke survivors (moderate certainty) [73]. Subgroup analysis suggested there might be a dose-relationship between the number of times Tai Chi was practiced per week and falls risk, but the findings were not statistically significant [73]. Falls risk factors also improved for older adults; however, the effects were unlikely to be clinically important (moderate or very low certainty) [71]. Mixed findings for falls risk factors in prefrail and frail older adults were also reported in a SR with no meta-analysis [60]. It was unclear if Tai Chi reduced the fear of falling due to very low certainty evidence [75].

#### Vestibular disorders

A SR with no meta-analysis of Tai Chi for vestibular rehabilitation reported improvements in dynamic balance, gait, and postural performance [72].

## Discussion

This critical overview comprehensively identified SRs of Tai Chi published in English, Chinese, and Korean languages that evaluated the effectiveness and safety of Tai Chi for health promotion, and disease prevention and management. Tai Chi was found to be generally safe, even for frail older adults; however, mild, transient discomfort during the first few weeks was reported by some participants. Clinically important benefits were most consistently reported for Parkinson’s disease, falls risk, knee osteoarthritis, low back pain, cardiovascular diseases including hypertension, and stroke.

Despite the large number of SRs, there were gaps in the available SR evidence. For the most part, the conditions most commonly evaluated by SRs generally matched those most commonly evaluated by primary studies. However, based on the bibliometric analyses of studies evaluating Tai Chi interventions [6, 7], the following had sufficient RCTs and were yet to be systematically reviewed. These were for people with depression, anxiety, drug dependency, musculoskeletal conditions of the hip, neck or shoulder, sarcopenia/frailty, diabetic neuropathy, or dysmenorrhea. Other evidence gaps included a paucity of SRs examining effects of Tai Chi for disease prevention. Except for stroke prevention, only indirect disease prevention outcomes (i.e. risk factors) such as hypertension, hyperlipidemia, HbA1c, falls prevention, balance, mobility, bone mineral density, and executive cognitive function were identified. Finally, whilst some SRs included healthy participants, with the exception of executive cognitive function [94], only a few evaluated the effects of Tai Chi for health promotion, quality of life, and wellbeing in healthy participants [63, 95]. This is despite an astounding number of RCTs, well over 100 [6, 7], evaluating these outcomes in healthy population groups.

It is noteworthy that a rapid search of PubMed, Embase, Cochrane Library, and CNKI databases for SRs published between 1 January 2021 and 5 June 2022 identified 38 potentially eligible SRs. Therefore, some of the identified gaps in the evidence may have been addressed and there may be higher quality, more comprehensive SRs than those included in this synthesis. Given this rapidly growing evidence base, an update of this overview is warranted.

### Limitations of the evidence

Rather than relying on the conclusions in the SRs, we appraised the evidence for the included estimates of effects. Notably, the GRADE certainty of the evidence for just over half of the estimates of effect was rated as low or very low. This was despite making a number concessions according to a pragmatic algorithm developed by Pollock et al. [38] when grading over 100 estimates for a Cochrane overview. Like Pollock et al. [38], the risk of bias for blinding focused on the study investigators rather than participants; the cut-off for the optimum information size for continuous outcomes was set at ≥ 200 participants, rather than the 400 tentatively recommended by GRADE [31]; and the cut-off for the *I*^2^ statistic when rating statistical heterogeneity was set at ≤ 75%. Additionally, although only a few instances of publication bias were identified, small sample sizes in many studies often reduced the imprecision of the estimates. Larger, higher-quality studies are therefore required to confirm many of the findings reported in this overview.

Limitations with the overall quality of the available SRs were another major concern. The majority of SRs were rated as low or critically low quality according to AMSTAR 2. Some of this reflected avoidable deficiencies in reporting. However, there were also numerous methodological deficiencies. Notably, many results were potentially conflated by pooling Tai Chi interventions of different intensity, frequency, and duration; comparison groups, regardless of whether they were likely to be an active or inactive control; and populations who may vary in their baseline severity, risk, prognosis, or clinical responsiveness. The impact of these decisions was often not appropriately investigated with subgroup or sensitivity analyses, or meta-regression. This may have exacerbated statistical heterogeneity and/or led to an over or underestimation of the effect sizes of Tai Chi. It also limited the ability to assess dose effects and determine how often and for how long Tai Chi needs to be practiced.

Issues with comprehensiveness and missing RCTs were another concern. Notably, during the final selection process, it became apparent that meeting the requirements for a comprehensive literature search strategy (item 4) and the overall AMSTAR 2 rating was no guarantee that all eligible primary studies were identified. For example, neither the high-quality Cochrane review of exercise interventions for falls [109, 110] nor its moderate quality 2020 update [111] was selected as they missed more of the eligible Tai Chi studies, partly due to not searching Chinese language databases. However, even when both English and Chinese language databases were searched, issues with missed studies were also identified in SRs for cancer, Parkinson’s disease, cardio/cerebrovascular diseases, and diabetes. It is highly recommended that reviewers pay greater attention to searching the reference lists not only of the included studies but also published SRs, consulting content experts in the field, and including experienced research librarians if possible to help optimize search strategies [112]. Further, considering Tai Chi originated in China and over half of the primary clinical studies have been published in Chinese [6, 7], it is difficult to justify not searching the major Chinese databases [112].

### Strengths and limitations of this overview

Strengths of this overview include the comprehensive literature search, transparent study selection, prioritizing the outcomes, low overlap of primary studies, and independent rating of the GRADE certainty of the evidence for each estimate of effect. In addition, we developed a pragmatic GRADE certainty rubric to facilitate a transparent and consistent rating process. However, by not evaluating the primary studies, variations among the interventions, and setting thresholds for some decisions, important nuances may have been overlooked that could have justified upgrading or downgrading the evidence [113]. For instance, the post hoc sensitivity analyses applied more rigorous criteria that led to the evidence certainty for 31 of the 120 estimates being downgraded one level. Yet this approach was still blunt, as it did not allow for instances when there are borderline concerns across a few domains that when combined may justify rating down one level rather than two. Indeed, there were numerous instances when the same evidence was given a different GRADE certainty rating by other reviewers [13, 19, 23, 27]. Therefore, whilst the findings provide a general overview of Tai Chi effectiveness and the evidence gaps, an appraisal of the primary studies, involvement of stakeholders, and consideration of context and expert consensus may still be required before making any critical decisions for Tai Chi clinical guidelines or policies [113].

Substantially more SRs were identified than equivalent reviews [23, 26, 27]. This was despite restricting our search to publications from 2010. There were no language limitations, and the major English and Chinese databases were searched. Nevertheless, some SRs are likely to have been missed, including SRs only indexed in databases of another language such as Korean, Japanese, or Thai.

Due to the large number of SRs, most of which were screened using a partially blinded process to help reduce the risk of selective reporting bias, it is possible that some populations and outcomes were also missed. However, we are confident that we have reported the important outcomes also highlighted in other SRs of SRs [8–27].

Efforts were made to minimize overlapping among the selected SRs, yet there were still a few instances of overlap (e.g. quality of life, mobility, mental health, and sleep) in which one or two RCTs were included in more than one of the reported estimates of effect. This may have biased results for the same outcome, either positively or negatively. However, unlike similar overviews of Tai Chi [23, 27], these limitations were offset in this overview by not reporting every estimate of effect for every SR and reporting the certainty of the evidence in the main summary of findings table irrespective of the effect size or statistical significance.

Finally, there was the potential for bias to be introduced during the selection and assessment processes, as three of the reviewers (GYY, JL, and PMW) were Tai Chi investigators (see “Competing interests” section). However, only GYY was directly involved in the screening, selection, and appraisal processes and was yet to publish a SR before the completion of this overview. Of the 210 included SRs, four were authored by reviewers of this overview [94, 114–116] and only one was selected in the final synthesis [94]. The SR was included despite being published in 2014 and rated as critically low quality because it was the only SR to meta-analyses cognitive performance outcomes for the healthy older adult population group.

## Conclusions

This overview comprehensively identified and critically appraised the most recent, best available SR evidence. Tai Chi was found to be generally safe and can be practiced at various levels of intensity by healthy adults, frail older adults, and people with chronic diseases. There was some evidence of beneficial physical, psychological, and quality of life outcomes from Tai Chi for a wide range of conditions. Given its multisystem effects, Tai Chi might be a suitable choice for those seeking a single intervention to help with numerous problems and symptoms.

However, the certainty in the evidence was often limited by the quality of the primary studies and their systematic reviews, clinical, methodological and statistical heterogeneity, and small sample sizes. Further research, including implementation and cost-effective research is warranted to support patient decisions, clinical practice, and policies.

## Supplementary Information

**Additional file 1.** PRISMA 2020 checklist.

**Additional file 2.** Database search results.

**Additional file 3.** Full-text articles excluded from updated 2020 search with reason for exclusion.

**Additional file 4.** Systematic review (SRs) with a meta-analysis of Tai Chi randomized controlled trials not selected for the evidence synthesis.

**Additional file 5.** AMSTAR 2 quality rating of systematic reviews.

**Additional file 6.** GRADE appraisal of the certainty of the evidence.

## Data Availability

The datasets supporting the conclusions of this article are included within the article and its additional files.

## Abbreviations

RCTs: Randomized controlled trial
NRSIs: Non-randomized studies of interventions
SRs: Systematic reviews
PROSPERO: International Prospective Register for Systematic Reviews
COMET: Core Outcome Measures in Effectiveness Trials
CNKI: China National Knowledge Infrastructure
VIP: Chinese Scientific Journal Database
REDCap: Research Electronic Data Capture
AMSTAR: A MeaSurement Tool to Assess systematic Reviews
PRISMA: Preferred Reporting Items for Systematic Reviews and Meta-analyses
GRADE: Grading of Recommendations, Assessment, Development and Evaluation
PCO: Population, condition and outcome
RR: Risk ratio
RD: Risk difference
NNT: Number needed to treat
CI: Confidence interval
MD: Mean difference
SMD: Standardized mean difference
MCID: Minimal clinically important difference
NI: No information
AE: Adverse effects
6MWD: 6-Min walk distance/test
BMD: Bone mineral density
BMI: Body mass index
BP: Blood pressure
MS: Multiple sclerosis
COPD: Chronic obstructive pulmonary disease
HDL-C: High-density lipoprotein cholesterol
LDL-C: Low-density lipoprotein cholesterol
LVEF: Cardiac left ventricular ejection fraction
MMSE: Mini-mental state examination
PANSS: Positive and negative syndrome scale
QoL: Quality of life
ADL: Routine activities of daily living/ routine lifestyle
Ex: Exercise (any type, including stretching)
HEd: Health education or other educational interventions
noRx: No treatment, control
Pharm: Pharmaceutical drugs / medication
Psych: Psychological interventions, counselling, support
PT: Physical therapy/physiotherapy
Rehab: Rehabilitation programs
TC: Tai Chi intervention
TCM: Traditional Chinese herbal medicine
Ucare: Usual care, conventional treatment, standard medical care
TC: Tai Chi
PROMs: Patient-reported outcome measures
TUG: Timed Up-and-Go
MMSE: Mini-mental state examination
