# Supplementary material for: Determining the safety and effectiveness of Tai Chi: a critical overview of 210 systematic reviews of controlled clinical trials
Source: Syst Rev. 2022 Dec 3;11:260. doi: 10.1186/s13643-022-02100-5 (PMC9719113; doi:10.1186/s13643-022-02100-5)
Supplement: Supplementary file 2 — Additional file 2. Database search results. [file 13643_2022_2100_MOESM2_ESM.pdf]

Additional file 2: Database search terms and results

1 Jan 2020 – 9 Dec 2020

Database	Search strategy syntax	Hits
PubMed	(((Taiji[Title/Abstract] OR Tai Ji[Title/Abstract] OR Tai-ji[Title/Abstract] OR Tai Chi[Title/Abstract] OR Tai Chi Chuan[Title/Abstract] OR Tai Chi Quan[Title/Abstract] OR Taijiquan[Title/Abstract]))) AND (("2020/01/31"[Date - Publication] : "2020/12/9"[Date - Publication]))	185
Cochrane Library	(((Taiji[Title/Abstract] OR Tai Ji[Title/Abstract] OR Tai-ji[Title/Abstract] OR Tai Chi[Title/Abstract] OR Tai Chi Chuan[Title/Abstract] OR Tai Chi Quan[Title/Abstract] OR Taijiquan[Title/Abstract]))) AND (("2020" [Date - Publication]))	93
EMBASE	(Taiji or Tai Ji or Tai-ji or Tai Chi or Tai Chi Chuan or Tai Chi Quan or Taijiquan).ab. and "2020".dp.	42
Medline	(Taiji or Tai Ji or Tai-ji or Tai Chi or Tai Chi Chuan or Tai Chi Quan or Taijiquan).ab. and "2020".dp.	27
Web of Science	TOPIC: (Taiji or Tai Ji or Tai-ji or Tai Chi or Tai Chi Chuan or Tai Chi Quan or Taijiquan)<br>Indexes=SCI-EXPANDED, SSCI, A&HCI, CPCI-S, CPCI-SSH, BKCI-S, BKCI-SSH, ESCI, CCR-EXPANDED, IC Timespan=2020	335
CNKI	(((Taiji (太极, Tai Chi) [Abstract]) OR Taiji Chuan (太极拳, Tai Chi) [Title/Abstract)])) AND (("2020/01/31"[Date - Publication] : "2020/12/9"[Date - Publication])))<br>(篇关摘=太极拳) OR (篇关摘=太极) 资源范围:总库；中英文扩展；时间范围：发表时间:2020-01-31 到 2020-12-10；更新时间:不限。文献分类:医药卫生科技	195
VIP	(((Taiji (太极, Tai Chi) [Abstract]) OR Taiji Chuan (太极拳, Tai Chi) [Title/Abstract)])) AND (("2020/01/31"[Date - Publication] : "2020/12/9"[Date - Publication])))<br>(文摘=太极拳) OR (文摘=太极) 资源范围:总库；中英文扩展；时间范围：发表时间:2020-01-31 到 2020-12-10；更新时间:不限。文献分类:医药卫生	121
Wanfang	(((Taiji (太极, Tai Chi) [Abstract]) OR Taiji Chuan (太极拳, Tai Chi) [Title/Abstract)])) AND (("2020/01/31"[Date - Publication]: "2020/12/9"[Date - Publication])))<br>(摘要:(太极拳) or 摘要:(太极)) and Date:2020-*	92
Sino-Med	(((Taiji (太极, Tai Chi) [Abstract]) OR Taiji Chuan (太极拳, Tai Chi) [Title/Abstract)])) AND (("2020/01/31"[Date - Publication]: "2020/12/9"[Date - Publication])))<br>(("太极拳"[摘要:智能] OR "太极"[摘要:智能]) AND Date:2020-*)	108
