# Supplementary material for: Determining the safety and effectiveness of Tai Chi: a critical overview of 210 systematic reviews of controlled clinical trials
Source: Syst Rev. 2022 Dec 3;11:260. doi: 10.1186/s13643-022-02100-5 (PMC9719113; doi:10.1186/s13643-022-02100-5)
Supplement: Supplementary file 3 — Additional file 3. Full-text articles excluded from updated 2020 search with reason for exclusion. [file 13643_2022_2100_MOESM3_ESM.pdf]

Additional file 3: Full-text articles excluded with reason for exclusion

Reason: Full text not available (English language databases)

1. Ren, Y., H. Xue, and A. Li, *Effect of microscope intervention on taijiquan on clinical rehabilitation of elderly patients with knee arthritis.* Investigacion clinica (Venezuela), 2020. **61**(1): p. 312-320.

Reason: Full text not available (Chinese language databases)

None

Reason: Duplicates (English language databases)

1. Liu, L., et al., *The effectiveness of tai chi in breast cancer patients: A systematic review and meta-analysis.* Complement Ther Clin Pract., 2020. **38:101078.**(doi): p. 10.1016/j.ctcp.2019.101078. Epub 2019 Dec 13.
2. Hoke, M., et al., *Balance Impairment and Effectiveness of Exercise Intervention in Chronic Obstructive Pulmonary Disease-A Systematic Review.* J Clin Neurosci., 2020. **76:261-263.**(doi): p. 10.1016/j.jocn.2020.04.040. Epub 2020 Apr 15.
3. Weber, M., et al., *Physical therapy interventions for older people with vertigo, dizziness and balance disorders addressing mobility and participation: a systematic review.* Int J Environ Res Public Health., 2020. **17**(18): p. 6556. doi: 10.3390/ijerph17186556.
4. Liu, X., et al., *The Effect of Tai Chi on Quality of Life in Centrally Obese Adults with Depression.* J Altern Complement Med, 2019. **25**(10): p. 1005-1008.
5. Cheng, D., et al., *The Effects of Tai Chi and Qigong on Immune Responses: A Systematic Review and Meta-Analysis.* Chin J Integr Med., 2020. **26**(5): p. 393-400. doi: 10.1007/s11655-020-3262-9. Epub 2020 Apr 29.
6. Guan, Y., et al., *Yoga and Tai Chi: a mind-body approach in managing respiratory symptoms in obstructive lung diseases.* J Rehabil Med., 2020. **52**(5): p. jrm00057. doi: 10.2340/16501977-2683.
7. Hu, L., et al., *Effects of Tai Chi exercise on cardiovascular disease risk factors and quality of life in adults with essential hypertension: A meta-analysis.* Clin Rehabil, 2020. **21**(269215520954343): p. 0269215520954343.
8. Liang, H., et al., *Meta-Analysis of Randomized Controlled Trials of the Effects of Tai Chi on Blood Pressure.* Heart Lung., 2020. **49**(4): p. 353-363. doi: 10.1016/j.hrtlng.2020.02.041. Epub 2020 Mar 12.
9. Zhao, J., *Effects of Tai Chi Chuan on the changes of bone mineral density of perimenopausal women. [Chinese].* Chinese Journal of Tissue Engineering Research, 2020. **24**(2): p. 176-180.
10. Xiao, Q., et al., *Tai Chi for preventing falls in the elderly: An overviews of systematic reviews. [Chinese].* Chinese Journal of Evidence-Based Medicine, 2020. **20**(2): p. 191-198.
11. Guo, C., et al., *Effects of Tai Chi training on the physical and mental health status in patients with chronic obstructive pulmonary disease: a systematic review and meta-analysis.* J Thorac Dis., 2020. **12**(3): p. 504-521. doi: 10.21037/jtd.2020.01.03.

Reason: Duplicates (Chinese language databases)

None

Reason: Not Tai Chi intervention (English language databases)

1. Toneti, B.F., et al., *Benefits of Qigong as an integrative and complementary practice for health: a systematic review.* Revista Latino-Americana De Enfermagem, 2020. **28**.

2. Urits, I., et al., *Acupuncture and Its Role in the Treatment of Migraine Headaches.* Neurol Ther, 2020. **9**(2): p. 375-394.
3. Yuen, C.S., et al., *The Effect of Conduction Exercise and Self-Acupressure in Treatment of Parkinson's Disease: A Pilot Study.* Evidence-Based Complementary and Alternative Medicine, 2020. doi:10.1155/2020/7950131

Reason: Not Tai Chi intervention (Chinese language databases)

None

Reason: Abstract, poster (English language databases)

1. *Study on the intervention of mindfulness cognitive training on anxiety of college students of traditional Chinese medicine.* Basic & clinical pharmacology & toxicology, 2020. **126**: p. 90-91.
2. Konuk Sener, D. and A. Karaca, *[Effects of long-term Tai Chi exercise on knee joints skin microvascular reactivity and transcutaneous oxygen partial pressure in middle and old aged people].* Nurs Health Sci., 2020. **22**(2): p. 328-338. doi: 10.1111/nhs.12733.
3. James, D., et al., *Effects of Tai Chi Easy on Body Fat Percentage, Sleep Quality, and Emotional Eating in Midlife and Older Women.* Annals of Behavioral Medicine, 2020. **54**: p. S613-S613.
4. Larkey, L., et al., *Preliminary Findings on Hrv and Stress Related to Body Fat % in a Tai Chi Easy/Qigong Intervention for Breast Cancer Survivors.* Annals of Behavioral Medicine, 2020. **54**: p. S537-S537.
5. Li, L., X. Yu, and H. Li, *The influence of Tai Chi on the physiological and biochemical indexes of female college students.* Basic & Clinical Pharmacology & Toxicology, 2020. **126**: p. 162-162.
6. Li, X., et al., *Influence of Taiji exercise on blood pressure and mood state of people with borderline hypertension.* Basic & clinical pharmacology & toxicology, 2020. **126**: p. 81-.
7. Li, X.Q., et al., *Analysis on the fusion of Tai Chi and the health preserving of traditional Chinese medicine based on big data.* Basic & Clinical Pharmacology & Toxicology, 2020. **126**: p. 125-125.
8. Manlapaz, D.G., et al., *Effectiveness of exergaming, Tai Chi, and physical therapy in improving dynamic balance and enjoyment of older adults with knee osteoarthritis.* Osteoarthritis and cartilage, 2020. **28**: p. S166-S167.
9. Song, Q.H., Y.H. Guo, and F. Wang, *Observation on the Effect of Long-term Taijiquan Exercise on Hand-eye Coordination and Limb Flexibility of the Elderly.* Basic & Clinical Pharmacology & Toxicology, 2020. **126**: p. 96-96.
10. Xie, M.N., J.Q. Zhang, and G.L. Yuan, *Effect of Taijiquan on Sub-healthy People under Brinell Microscopy.* Acta Microscopica, 2020. **29**(2): p. 1044-1052.
11. Kalebota, N., et al., *Effects of Tai Chi Exercise on Pain, Functional Status and Quality of Life in People with Osteoarthritis and Inflammatory Arthritis.* Annals of the Rheumatic Diseases, 2020. **79**: p. 1918-1919.
12. Tate, L.M., et al., *A Pilot Clinical Tai Chi Program: Effects on Veterans' Functional Outcomes.* Journal of the American Geriatrics Society, 2020. **68**: p. S205-S205.
13. Hai-Jun, Z., L. Yuan-Le, and G. Xiao-Tao, *Improving memory and executive function of the elderly with mild congnitive impairment-a smart health care approach.* Journal of medical imaging and health informatics, 2020. **10**(1): p. 44-48.
14. Koren, Y., S. Leveille, and T.J. You, *Tai Chi Interventions Promoting Social Interaction and Participation Among Older Adults: A Systematic Review.* Nursing Research, 2020. **69**(3): p. E145-E146.
15. Liu, T., S.Y. Chair, and A.W. Chan, *Group- Plus Home-based Tai Chi Program Improves Physical Function and Psychosocial Well-being Among Patients With Coronary Heart Disease.* Circulation, 2020. **141**.

Reason: Abstract, poster, no full-text available (Chinese language databases)

None

Reason: Tai chi with co-interventions (English language databases)

1. Anupama, D.S., et al., *Effect of exercise on bone mineral density and quality of life among postmenopausal women with osteoporosis without fracture: A systematic review.* Int J Orthop Trauma Nurs, 2020. **39**: p. 100796.
2. Behzadmehr, R., et al., *Effect of complementary and alternative medicine interventions on cancer related pain among breast cancer patients: A systematic review.* Complementary Therapies in Medicine, 2020. **49**.
3. Biazus-Sehn, L.F., et al., *Effects of physical exercise on cognitive function of older adults with mild cognitive impairment: A systematic review and meta -analysis.* Archives of Gerontology and Geriatrics, 2020. **89**.
4. Chen, F.T., et al., *Effects of Exercise Training Interventions on Executive Function in Older Adults: A Systematic Review and Meta-Analysis.* Sports Medicine, 2020. **50**(8): p. 1451-1467.
5. Chen, K., et al., *Effect of Exercise on Quality of Life in Parkinson's Disease: A Systematic Review and Meta-Analysis.* Parkinsons Disease, 2020. doi:10.1155/2020/3257623
6. de Souza, F., et al., *Effectiveness of martial arts exercise on anthropometric and body composition parameters of overweight and obese subjects: a systematic review and meta-analysis.* BMC Public Health, 2020. **20**(1).
7. de Zoete, R.M., et al., *Comparative effectiveness of physical exercise interventions for chronic non-specific neck pain: a systematic review with network meta-analysis of 40 randomised controlled trials.* Br J Sports Med, 2020.
8. de Zoete, R.M.J., et al., *The effectiveness of general physical exercise for individuals with chronic neck pain: a systematic review of randomised controlled trials.* European Journal of Physiotherapy, 2020. **22**(3): p. 141-147.
9. Dixit, S., K. Gular, and F. Asiri, *Effect of diverse physical rehabilitative interventions on static postural control in diabetic peripheral neuropathy: a systematic review.* Physiother Theory Pract, 2020. **36**(6): p. 679-690.
10. Ford, C.G., et al., *Mindfulness and Meditative Movement Interventions for Men Living With Cancer: A Meta-analysis.* Annals of Behavioral Medicine, 2020. **54**(5): p. 360-373.
11. Gill, B.K., et al., *Non-pharmacological depression therapies for older Chinese adults: A systematic review & meta-analysis.* Archives of Gerontology and Geriatrics, 2020. **88**.
12. Jin, X.H., et al., *The Impact of Mind-Body Exercises on Motor Function, Depressive Symptoms, and Quality of Life in Parkinson's Disease: A Systematic Review and Meta-Analysis.* International Journal of Environmental Research and Public Health, 2020. **17**(1).
13. Li, R.J., et al., *Effectiveness of Traditional Chinese Exercise for Symptoms of Knee Osteoarthritis: A Systematic Review and Meta-Analysis of Randomized Controlled Trials.* International Journal of Environmental Research and Public Health, 2020. **17**(21).
14. Li, Z.M., et al., *Mind-Body Exercise for Anxiety and Depression in COPD Patients: A Systematic Review and Meta-Analysis.* International Journal of Environmental Research and Public Health, 2020. **17**(1).
15. Liu, F., et al., *The effect of tai chi and Qigong exercise on depression and anxiety of individuals with substance use disorders: a systematic review and meta-analysis.* Bmc Complementary Medicine and Therapies, 2020. **20**(1).
16. Miller, K.J., et al., *Comparative effectiveness of three exercise types to treat clinical depression in older adults: A systematic review and network meta-analysis of randomised controlled trials.* Ageing Research Reviews, 2020. **58**.
17. Nayeri, N.D., et al., *The Effect of Complementary and Alternative Medicines on Quality of Life in Patients with Breast Cancer: A Systematic Review.* Indian Journal of Palliative Care, 2020. **26**(1): p. 95-104.

18. Oberoi, D., et al., *Factors related to dropout in integrative oncology clinical trials: interim analysis of an ongoing comparative effectiveness trial of mindfulness-based cancer recovery and Tai chi/Qigong for cancer health (The MATCH study).* BMC research notes, 2020. **13**(1): p. 342-.
19. Oh, B., et al., *The Effects of Tai Chi and Qigong on Immune Responses: A Systematic Review and Meta-Analysis.* Medicines (Basel), 2020. **7**(7).
20. Siddarth, P., et al., *Predictors of Cognitive Improvement Following Treatment for Late-Life Depression.* Journal of Geriatric Psychiatry and Neurology, 2020.
21. Skelly, A.C., et al., *Noninvasive Nonpharmacological Treatment for Chronic Pain: A Systematic Review Update* 2020.
22. Sweeting, J., et al., *Physical activity interventions for adults who are visually impaired: a systematic review and meta-analysis.* Bmj Open, 2020. **10**(2).
23. Takemura, N., et al., *Effectiveness of aerobic exercise and mind-body exercise in cancer patients with poor sleep quality: A systematic review and meta-analysis of randomized controlled trials.* Sleep Medicine Reviews, 2020. **53**.
24. Thukral, N., J. Kaur, and M. Malik, *A systematic review and meta-analysis on efficacy of exercise on posture and balance in patients suffering from Diabetic Neuropathy.* Curr Diabetes Rev, 2020.
25. Tran, B.X., et al., *Global mapping of interventions to improve quality of life using mind-body therapies during 1990-2018.* Complementary Therapies in Medicine, 2020. **49**.
26. Vanderlinden, J., F. Boen, and J.G.Z. van Uffelen, *Effects of physical activity programs on sleep outcomes in older adults: a systematic review.* International Journal of Behavioral Nutrition and Physical Activity, 2020. **17**(1).
27. Weber, M., et al., *Effects of Mind-Body Interventions Involving Meditative Movements on Quality of Life, Depressive Symptoms, Fear of Falling and Sleep Quality in Older Adults: A Systematic Review with Meta-Analysis.* International Journal of Environmental Research and Public Health, 2020. **17**(18).
28. Wong, R.M.Y., et al., *The effectiveness of exercises on fall and fracture prevention amongst community elderlies: A systematic review and meta-analysis.* Journal of Orthopaedic Translation, 2020. **24**: p. 58-65.
29. Xia, T.W., et al., *Meditative Movements for Patients with Type 2 Diabetes: A Systematic Review and Meta-Analysis.* Evidence-Based Complementary and Alternative Medicine, 2020. doi:10.1155/2020/5745013
30. Yao, L.Q., et al., *Traditional Chinese exercise for cancer-related sleep disturbance: A systematic review and descriptive analysis of randomized controlled trials.* Complementary Therapies in Clinical Practice, 2020. **40**.
31. Ye, M.Z., et al., *The effect of mind-body exercise on memory in older adults: a systematic review and meta-analysis.* Aging Clinical and Experimental Research.
32. Zampogna, B., et al., *The Role of Physical Activity as Conservative Treatment for Hip and Knee Osteoarthritis in Older People: A Systematic Review and Meta-Analysis.* Journal of Clinical Medicine, 2020. **9**(4).

Reason: Tai chi with co-interventions (Chinese language databases)

None

Reason: Other type of tai chi review article (English language databases)

1. Deuel, L.M. and L.C. Seeberger, *Complementary Therapies in Parkinson Disease: a Review of Acupuncture, Tai Chi, Qi Gong, Yoga, and Cannabis.* Neurotherapeutics, 2020.
2. Easwaran, K., et al., *Effectiveness of Tai Chi for health promotion for adults with health conditions: a scoping review of Meta-analyses.* Disability and Rehabilitation, 2020.

3. McLaughlin, E.C., et al., *Balance and functional training and health in adults: an overview of systematic reviews.* Applied Physiology Nutrition and Metabolism, 2020. **45**(10): p. S180-S196.
4. Phuphanich, M.E., et al., *Movement-Based Therapies in Rehabilitation.* Physical medicine and rehabilitation clinics of North America, 2020. **31**(4): p. 577-591.
5. Pretty, J. and J. Barton, *Nature-Based Interventions and Mind-Body Interventions: Saving Public Health Costs Whilst Increasing Life Satisfaction and Happiness.* International Journal of Environmental Research and Public Health, 2020. **17**(21).
6. Ratarasarn, K. and A. Kundu, *Yoga and Tai Chi: a mind-body approach in managing respiratory symptoms in obstructive lung diseases.* Curr Opin Pulm Med, 2020. **26**(2): p. 186-192.
7. Tarsha, M.S., S. Park, and S. Tortora, *Body-Centered Interventions for Psychopathological Conditions: A Review.* Frontiers in Psychology, 2020. **10**.
8. Wu, S.Y.F., T. Brown, and M.L. Yu, *Older Adults' Psychosocial Responses to a Fear of Falling: A Scoping Review to Inform Occupational Therapy Practice.* Occupational Therapy in Mental Health, 2020. **36**(3): p. 207-243.
9. Zhong, D., et al., *Tai Chi for improving balance and reducing falls: An overview of 14 systematic reviews.* Ann Phys Rehabil Med., 2020. **63**(6): p. 505-517. doi: 10.1016/j.rehab.2019.12.008. Epub 2020 Jan 22.
10. Bayrakdaroglu, S. and A. Tekin, *The Effect of Defense Sports on the Treatment of Attention-Deficit/Hyperactivity Disorder in Children.* International Journal of Applied Exercise Physiology, 2020. **9**(8): p. 47-54.
11. Qin, Y., et al., *The Beneficial Effect of Traditional Chinese Exercises on the Management of Obesity.* Evid Based Complement Alternat Med, 2020. **2020**: p. 2321679.
12. Guo, S., et al., *Comparative efficacy of seven exercise interventions for symptoms of depression in college students: A network of meta-analysis.* Medicine (Baltimore), 2020. **99**(47): p. e23058.
13. Cheng, D., et al., *Research on Function and Mechanism of Tai Chi on Cardiac Rehabilitation.* Chin J Integr Med., 2020. **26**(5): p. 393-400. doi: 10.1007/s11655-020-3262-9. Epub 2020 Apr 29.
14. Zou, L., et al., *Tai Chi for Chronic Illness Management: Synthesizing Current Evidence from Meta-Analyses of Randomized Controlled Trials.* Am J Med, 2020.
15. Nyman, S.R., *Tai Chi for the Prevention of Falls Among Older Adults: A Critical Analysis of the Evidence.* J Aging Phys Act, 2020: p. 1-10.
16. Huang, J., et al., *The Effectiveness of Tai Chi in Patients With Breast Cancer: An Overview of Systematic Reviews and Meta-Analyses.* Journal of Pain and Symptom Management., 2020.

Reason: Other type of tai chi review article (Chinese language databases)

1. 顾迎春等, 太极拳运动对心血管疾病影响研究进展. 中国循证心血管医学杂志, 2020. 12(9): 第 1147-1149 页.
2. 梁伟等, 太极拳改善帕金森病运动症状和非运动症状疗效研究进展. 环球中医药, 2020
3. 肖奇蔚等, 太极预防老年跌倒的系统评价再评价. 中国循证医学杂志, 2020. 20(02): 第 191-198 页.

Reason: Other tai chi article or study (English language databases)

1. Bouri, M., A. Khalfallah, and M.S. Bouhlel, *Tai Chi Care: An Exergaming Software using Microsoft Kinect V2 for Blind or Low Vision Person during Confinement.* International Journal of Advanced Computer Science and Applications, 2020. **11**(7): p. 147-153.

2. Cetin, S.Y., B.B. Calik, and A. Ayan, *Investigation of the effectiveness of Tai Chi exercise program in patients with scleroderma: A randomized controlled study.* Complement Ther Clin Pract, 2020. **40**: p. 101181.
3. Hu, X., Z. Lai, and L. Wang, *Effects of Taichi exercise on knee and ankle proprioception among individuals with knee osteoarthritis.* Research in sports medicine (Print), 2020. **28**(2): p. 268-278.
4. Li, J., et al., *Tai Chi plus standard care versus other exercise plus standard care for people with schizophrenia or related disorders.* Cochrane Database of Systematic Reviews, 2020(2).
5. Luberto, C.M., et al., *Exploring correlates of improved depression symptoms and quality of life following tai chi exercise for patients with heart failure.* ESC heart failure, 2020.
6. Ma, C., et al., *Clinical effect of Tai Chi spinal exercise on spinal motor function in patients with axial spondyloarthritis.* International journal of clinical and experimental medicine, 2020. **13**(2): p. 673-681.
7. Ma, G., *High Boron Silicon Nanotubes Combined with Tai Chi Exercise Rehabilitation Therapy in the Treatment of Knee Arthritis Patients.* Journal of Chemistry, 2020. **2020**.
8. Montayre, J., et al., *What makes community-based physical activity programs for culturally and linguistically diverse older adults effective? A systematic review.* Australasian Journal on Ageing.
9. Sherman, K.J., et al., *T'ai Chi for Chronic Low Back Pain in Older Adults: a Feasibility Trial.* Journal of alternative and complementary medicine (New York, N.Y.), 2020. **26**(3): p. 176-189.
10. Chen, C.Y. and N.G. Muggleton, *Electrophysiological investigation of the effects of Tai Chi on inhibitory control in older individuals.* Progress in Brain Research, 2020. **253**: p. 229-242.
11. Mori, K., et al., *Impact of Tai Chi Yuttari-exercise on arteriosclerosis and physical function in older people.* Arch Gerontol Geriatr, 2020. **87**: p. 104011.
12. Liu, J., et al., *The 24-Form Tai Chi Improves Anxiety and Depression and Upregulates miR-17-92 in Coronary Heart Disease Patients After Percutaneous Coronary Intervention.* Front Physiol, 2020. **11**: p. 149.
13. While, A.E., *Falls and older people: preventative interventions.* Br J Community Nurs, 2020. **25**(6): p. 288-292.
14. Gerritsen, R.J.S., et al., *No panacea? Tai Chi enhances motoric but not executive functioning in a normal aging population.* Neuropsychol Dev Cogn B Aging Neuropsychol Cogn, 2020: p. 1-24.
15. Barrado-Martin, Y., et al., *People living with dementia and their family carers' adherence to home-based Tai Chi practice.* Dementia (London), 2020: p. 1471301220957758.
16. Zhao, J., et al., *The effects of sitting Tai Chi on physical and psychosocial health outcomes among individuals with impaired physical mobility.* Medicine (Baltimore), 2020. **99**(34): p. e21805.
17. Sam-Kit Tin, T., et al., *Effects of A Short-term Cardio Tai Chi Program on Cardiorespiratory Fitness and Hemodynamic Parameters in Sedentary Adults: A Pilot Study.* J Acupunct Meridian Stud, 2020. **13**(1): p. 12-18.
18. Qi, M., et al., *Feasibility of a Tai Chi with Thera-Band Training Program: A Pilot Study.* Int J Environ Res Public Health., 2020. **17**(22): p. 8462. doi: 10.3390/ijerph17228462.
19. Fiorini, A.C., et al., *Tai chi and Parkinson's disease: a bevy of benefits.* Disabil Rehabil, 2020: p. 1-2.

Reason: Other tai chi article or study (Chinese language databases)

None
