# Supplementary material for: Determining the safety and effectiveness of Tai Chi: a critical overview of 210 systematic reviews of controlled clinical trials
Source: Syst Rev. 2022 Dec 3;11:260. doi: 10.1186/s13643-022-02100-5 (PMC9719113; doi:10.1186/s13643-022-02100-5)
Supplement: Supplementary file 4 — Additional file 4. Systematic review (SRs) with a meta-analysis of Tai Chi randomized controlled trials not selected for the evidence synthesis. [file 13643_2022_2100_MOESM4_ESM.pdf]

Additional file 4: Systematic reviews (SRs) not selected for the evidence synthesis

STAGE 1 SELECTION PROCESS

The following SRs with a meta-analysis of RCTs were not selected for the evidence synthesis as other SRs with a meta-analysis were more recent and/or comprehensive.

Published in Chinese

Reason: other SRs with a meta-analysis of RCTs were more recent and/or comprehensive

2011

1. 李红果与徐志文, 太极拳治疗原发性高血压的系统评价. 文体用品与科技, 2011(07): 第 35-37 页.

2013 -2014

2. 晏利姣, 曹卉娟与郝玉芳, 太极拳改善乳腺癌患者生存质量随机对照试验的系统评价. 中国康复理论与实践, 2013(06): 第 592-597 页.
3. 赵媛等, 太极拳运动对老年人平衡功能和跌倒预防效果的 Meta 分析. 中国循证医学杂志, 2013. 13(3).
4. 拜争刚等, 太极拳预防老年抑郁症有效性的系统评价. 中国老年学杂志, 2014(10): 第 2716-2719 页.

2015 - 2016

5. 张明军与叶学球, 太极拳练习对中老年人血压影响的 Meta 分析. 福建体育科技, 2015(03): 第 46-49 页.
6. 王继红等, 太极拳对老年慢性阻塞性肺疾病患者肺功能和运动耐力影响的 Meta 分析. 中国组织工程研究, 2015(05): 第 815-820 页.
7. 田凌云等, 太极拳对稳定期慢性阻塞性肺疾病患者康复影响的 Meta 分析. 中国老年学杂志, 2015(05): 第 1286-1289 页.
8. 黄毛毛, 穆卫强与郑国华, 太极拳锻炼对老年人睡眠质量影响的系统评价. 康复学报, 2015(04): 第 56-61 页.
9. 李晓军等, 太极拳对老年人单足站立时间影响差异性的 Meta 分析. 康复学报, 2016. 26(5).
10. 潘元青等, 太极拳辅助康复治疗乳腺癌术后不良反应的系统评价. 兰州大学学报(医学版), 2016(03): 第 64-72 页.
11. 瞿超艺等, 不同运动方式对帕金森病相关指标的作用——系统综述. 中国运动医学杂志, 2016(09): 第 855-868+894 页.
12. 蔡璐与李晓, 太极拳治疗原发性高血压疗效 Meta 分析. 中医药临床杂志, 2016(10): 第 1425-1428 页.
13. 谢辉等, 太极拳治疗骨关节炎的系统评价和 Meta 分析. 中华中医药杂志, 2016(05): 第 1863-1867 页.

14. 邵威等, 太极拳运动对稳定期慢性阻塞性肺疾病患者肺功能康复效果的 meta 分析. 中国康复医学杂志, 2016. 31(5).
15. 张业廷等. 太极拳运动对Ⅱ型糖尿病患者的影响--Meta 分析. in 2015 全国体育保健康复学术会议. 2016. 苏州.

2017 - 2018

16. 刘永进等, 太极拳锻炼对 2 型糖尿病患者糖脂代谢和生活质量影响的系统评价. 康复学报, 2017(04): 第 55-59+64 页.
17. 刘米娜与李学斌, 太极延缓社区中老年人认知功能衰退有效性的系统评价. 社会建设, 2017(04): 第 42-56 页.
18. 唐青等, 太极拳在 2 型糖尿病患者中应用效果的 Meta 分析. 现代预防医学, 2017(14): 第 2516-2521 页.
19. 张业廷与李慧军, 太极拳运动对中老年原发性高血压患者的影响 ——Meta 分析. 运动精品, 2017. 36(6).
20. 李翔等, 太极拳治疗下腰痛临床疗效的 Meta 分析. 康复学报, 2017(04): 第 60-64 页.
21. 王春艳, 传统体育运动对糖尿病康复作用的系统评价及 Meta 分析, 2017, 山东师范大学.
22. 王齐, 太极拳治疗 2 型糖尿病随机对照实验的系统评价与 meta 分析, 2017, 北京体育大学.
23. 王晨等, 短期的太极拳结合常规康复训练治疗脑卒中偏瘫患者平衡能力、运动功能的 meta 分析. 中国康复医学杂志, 2018(11): 第 1322-1328 页.
24. 曾令烽等, 传统运动疗法干预对膝骨关节炎患者疼痛改善及关节功能影响的系统评价. 中华中医药杂志, 2018(05): 第 2132-2139 页.
25. 邱亚娟等, 太极拳对老年慢性阻塞性肺病患者肺功能和体力状况的影响. 中国老年学杂志, 2018(01): 第 151-153 页.

2019 - 2020

26. 张永鹏等, 太极拳对原发性高血压患者降压效果的系统评价与 Meta 分析. 体育科研, 2019(01): 第 96-104 页.
27. 王晨与周立峰, 太极拳对慢性阻塞性肺疾病患者肺功能、运动能力和生活质量影响的 meta 分析. 现代医药卫生, 2020. 36(22): 第 3651-3656 页.
28. 陈慧娟, 太极拳对腰椎间盘突出影响的 Meta 分析. 龙岩学院学报, 2020. 38(02): 第 112-117 页.
29. 宋咪等, 太极对老年人跌倒预防效果的 Meta 分析. 海南医学院学报, 2020. 26(07): 第 545-551 页.

Published in English
Reason: other SRs with a meta-analysis of RCTs were more recent and/or comprehensive
2010 - 2012

1. Lee, M.S., T.Y. Choi, and E. Ernst, *Tai chi for breast cancer patients: a systematic review.* Breast Cancer Res Treat, 2010. **120**(2): p. 309-16.
2. Lee, M.S., et al., *Tai chi for lowering resting blood pressure in the elderly: a systematic review.* J Eval Clin Pract, 2010. **16**(4): p. 818-24.
3. Logghe, I.H.J., et al., *The effects of Tai Chi on fall prevention, fear of falling and balance in older people: a meta-analysis.* Preventive medicine, 2010. **51**(3-4): p. 222-227.
4. Escalante, Y., A. Garcia-Hermoso, and J.M. Saavedra, *Effects of exercise on functional aerobic capacity in lower limb osteoarthritis: a systematic review.* J Sci Med Sport, 2011. **14**(3): p. 190-8.
5. Kang, J.W., et al., *T'ai chi for the treatment of osteoarthritis: a systematic review and meta-analysis.* BMJ Open, 2011. **1**(1): p. e000035.
6. Lee, M.S., et al., *Tai chi for management of type 2 diabetes mellitus: a systematic review.* Chin J Integr Med, 2011. **17**(10): p. 789-93.
7. Leung, D.P.K., et al., *Tai Chi as an Intervention to Improve Balance and Reduce Falls in Older Adults: A Systematic and Meta-analytical Review.* Alternative Therapies in Health and Medicine, 2011. **17**(1): p. 40-48.
8. Liu, B., et al., *Effects of tai chi on lower-limb myodynamia in the elderly people: a meta-analysis.* J Tradit Chin Med, 2011. **31**(2): p. 141-6.
9. Gillespie, L.D., et al., *Interventions for preventing falls in older people living in the community.* Cochrane Database of Systematic Reviews, 2012(9).

2013 - 2014

10. Chi, I., et al., *Tai chi and reduction of depressive symptoms for older adults: A meta-analysis of randomized trials.* Geriatrics & Gerontology International, 2013. **13**(1): p. 3-12.
11. Lauche, R., et al., *A systematic review and meta-analysis of Tai Chi for osteoarthritis of the knee.* Complement Ther Med, 2013. **21**(4): p. 396-406.
12. Pan, L., Y. Guo, and J. Yan, *Effects of Tai Chi training on exercise capacity and quality of life in patients with chronic heart failure: A meta-analysis.* European Journal of Heart Failure, 2013. **15**(3): p. 316-323.
13. Raman, G., et al., *Tai Chi Improves Sleep Quality in Healthy Adults and Patients with Chronic Conditions: A Systematic Review and Meta-analysis.* J Sleep Disord Ther, 2013. **2**(6).
14. Wang, J., et al., *Tai Chi for Essential Hypertension.* Evidence-Based Complementary and Alternative Medicine, 2013.
15. Wu, Y., et al., *The effects of Tai Chi exercise on cognitive function in older adults: A meta-analysis.* Journal of Sport and Health Science, 2013. **2**(4): p. 193-203.
16. Yan, J.H., et al., *Efficacy of Tai Chi on pain, stiffness and function in patients with osteoarthritis: a meta-analysis.* PLoS One, 2013. **8**(4): p. e61672.
17. Yan, J.H., et al., *Effects of Tai Chi in patients with chronic obstructive pulmonary disease: preliminary evidence.* PLoS One, 2013. **8**(4): p. e61806.
18. Wu, W.B., et al., *Effects of Tai Chi on exercise capacity and health-related quality of life in patients with chronic obstructive pulmonary disease: a systematic review and meta-analysis.* International Journal of Chronic Obstructive Pulmonary Disease, 2014. **9**: p. 1253-1263.
19. Yan, J.H., et al., *Lack of efficacy of Tai Chi in improving quality of life in breast cancer survivors: a systematic review and meta-analysis.* Asian Pac J Cancer Prev, 2014. **15**(8): p. 3715-20.
20. Yang, Y., et al., *Tai Chi for improvement of motor function, balance and gait in Parkinson's disease: a systematic review and meta-analysis.* PLoS One, 2014. **9**(7): p. e102942.
21. Zeng, Y., et al., *Health benefits of qigong or tai chi for cancer patients: A systematic review and meta-analyses.* Complementary Therapies in Medicine, 2014. **22**(1): p. 173-186.

2015 - 2016

23. Du, S.Z., et al., *Taichi exercise for self-rated sleep quality in older people: A systematic review and meta-analysis.* International Journal of Nursing Studies, 2015. **52**(1): p. 368-379.
24. Huang, Y.J. and X.M. Liu, *Improvement of balance control ability and flexibility in the elderly Tai Chi Chuan (TCC) practitioners: A systematic review and meta-analysis.* Archives of Gerontology and Geriatrics, 2015. **60**(2): p. 233-238.
25. Lee, M.S., et al., *A systematic review and meta-analysis of tai chi for treating type 2 diabetes.* Maturitas, 2015. **80**(1): p. 14-23.
26. Liu, X., et al., *A systematic review and meta-analysis of the effects of Qigong and Tai Chi for depressive symptoms.* Complement Ther Med, 2015. **23**(4): p. 516-34.
27. Pan, Y.Q., et al., *Tai Chi Chuan Exercise for Patients with Breast Cancer: A Systematic Review and Meta-Analysis.* Evidence-Based Complementary and Alternative Medicine, 2015.
28. Zheng, G.H., et al., *Tai Chi and the Protection of Cognitive Ability A Systematic Review of Prospective Studies in Healthy Adults.* American Journal of Preventive Medicine, 2015. **49**(1): p. 89-97.
29. Zhou, J., et al., *A Meta-Analysis on the Efficacy of Tai Chi in Patients with Parkinson's Disease between 2008 and 2014.* Evid Based Complement Alternat Med, 2015. **2015**: p. 593263.
30. Chang, W.D., et al., *The Effects of Tai Chi Chuan on Improving Mind-Body Health for Knee Osteoarthritis Patients: A Systematic Review and Meta-Analysis.* Evidence-Based Complementary and Alternative Medicine, 2016.
31. Hu, Y.N., et al., *Effect of Tai Chi Exercise on Fall Prevention in Older Adults: Systematic Review and Meta-analysis of Randomized Controlled Trials.* International Journal of Gerontology, 2016. **10**(3): p. 131-136.
32. Ngai, S.P.C., A.Y.M. Jones, and W.W.S. Tam, *Tai Chi for chronic obstructive pulmonary disease (COPD).* Cochrane Database of Systematic Reviews, 2016(6).
33. Sun, Z., et al., *Effects of tai chi exercise on bone health in perimenopausal and postmenopausal women: a systematic review and meta-analysis.* Osteoporos Int, 2016. **27**(10): p. 2901-11.
34. Zhao, Y. and Y. Wang, *Tai Chi as an intervention to reduce falls and improve balance function in the elderly: a meta-analysis of randomized controlled trials.* Chin Nurs Res, 2016(3): p. 28-33.

2017 - 2018

35. Li, Y.F., et al., *The effect of Tai Chi exercise on motor function and sleep quality in patients with stroke: A meta-analysis.* International Journal of Nursing Sciences, 2017. **4**(3): p. 314-321.
36. Liu, F.H. and S. Wang, *Effect of Tai Chi on bone mineral density in postmenopausal women: A systematic review and meta-analysis of randomized control trials.* Journal of the Chinese Medical Association, 2017. **80**(12): p. 790-795.
37. Lomas-Vega, R., et al., *Tai Chi for Risk of Falls. A Meta-analysis.* J Am Geriatr Soc, 2017. **65**(9): p. 2037-2043.
38. Mazzarin, C.M., et al., *Effects of dance and of Tai Chi on functional mobility, balance, and agility in Parkinson Disease.* Top Geriatr Rehabil, 2017. **33**(4): p. 262-272.
39. Zhang, J.M., *Effects of Tai Chi on the static balance ability and the proprioception of lower limbs in middle-aged and elderly people: a meta-analysis.* Journal of Chaohu College, 2017. **19**(6): p. 66-71.
40. Zhang, Y.T., et al., *Effect of Tai Chi exercise for hypertension: a meta-analysis of randomized controlled trials*, in *2016 International Conference on Medicine Sciences and Bioengineering*, J.Q. Cheng, et al., Editors. 2017.

41. Gendron, L.M., et al., *Active mind-body movement therapies as an adjunct to or in comparison with pulmonary rehabilitation for people with chronic obstructive pulmonary disease.* Cochrane Database of Systematic Reviews, 2018(10).
42. Li, G.Y., et al., *Effects of Tai Chi on balance and gait in stroke survivors: A systematic meta-analysis of randomized controlled trials.* J Rehabil Med, 2018. **50**(7): p. 582-588.
43. Liu, T., et al., *Effects of Tai Chi-based cardiac rehabilitation on aerobic endurance, psychosocial well-being, and cardiovascular risk reduction among patients with coronary heart disease: A systematic review and meta-analysis.* European Journal of Cardiovascular Nursing, 2018. **17**(4): p. 368-383.
44. Wayne, P.M., et al., *Tai Chi and Qigong for cancer-related symptoms and quality of life: a systematic review and meta-analysis.* J Cancer Surviv, 2018. **12**(2): p. 256-267.
45. Wu, S.Z., et al., *Effect of Tai Chi Exercise on Balance Function of Stroke Patients: A Meta-Analysis.* Medical Science Monitor Basic Research, 2018. **24**: p. 210-215.
46. Yang, Y.L., et al., *The effect of Tai Chi on cardiorespiratory fitness for coronary disease rehabilitation: A systematic review and meta-analysis.* Frontiers in Physiology, 2018. **8** (1091).

2019 - 2020

47. Liu, H.H., et al., *Effects of Tai Chi Exercise on Reducing Falls and Improving Balance Performance in Parkinson's Disease: A Meta-Analysis.* Parkinsons Dis, 2019. **2019**: p. 9626934.
48. Zou, L.Y., et al., *Effects of Tai Chi on Lower Limb Proprioception in Adults Aged Over 55: A Systematic Review and Meta-Analysis.* Archives of Physical Medicine and Rehabilitation, 2019. **100**(6): p. 1102-1113.
49. Liu, D.C., et al., *The Efficacy of Tai Chi and Qigong Exercises on Blood Pressure and Blood Levels of Nitric Oxide and Endothelin-1 in Patients with Essential Hypertension: A Systematic Review and Meta-Analysis of Randomized Controlled Trials.* Evidence-Based Complementary and Alternative Medicine, 2020. **2020**.
50. Nduwimana, I., et al., *Effectiveness of walking versus mind-body therapies in chronic low back pain A systematic review and meta-analysis of recent randomized controlled trials.* Medicine, 2020. **99**(35).

STAGE 2 SELECTION PROCESS

The following SRs with overlapping populations and/or outcomes were not selected for the evidence synthesis as another SR was more comprehensive and/or of higher quality.

Published in Chinese

Reason: the SR with a meta-analysis selected for evidence synthesis was more comprehensive and/or higher quality

2016

1. 李淑珍等, 太极拳运动对脑卒中患者平衡功能影响的系统评价. 康复学报, 2016(02): 第 57-62 页.
2. 苗雨, 太极拳对脑卒中后患者平衡功能效果的 Meta 分析. 天津护理, 2016(06): 第 501-504 页.

2017 - 2018

3. 张嘉祺等, 太极拳训练对精神分裂症患者阴性症状和活动参与的 Meta 分析. 中国循证医学杂志, 2017(02): 第 206-212 页.
4. 张建国, 唐纯志与孔令朔, 太极拳运动对老年人认知功能影响的系统评价与 Meta 分析. 中医杂志, 2017(17): 第 1473-1477 页.
5. 魏洪悦等, 太极拳对心力衰竭患者作用效果的系统评价. 中国循证医学杂志, 2017(06): 第 677-684 页.
6. 金成吉, 张自云与解超, 太极拳对中老年原发性高血压患者血压水平影响的 Meta 分析. 现代预防医学, 2018(18): 第 3446-3451 页.
7. 孙凯丽, 社区轻度认知损害老人中医护理干预研究, 2018, 湖北中医药大学.
8. 李建超等, 太极拳对心力衰竭患者心功能及生活质量影响的 meta 分析. 慢性病学杂志, 2018(04): 第 369-374 页.
9. 陈梓, 喻惠丹与张凝凝, 太极拳运动对冠心病患者生理指标及生活质量影响的系统评价. 解放军护理杂志, 2018(01): 第 8-14 页.

2019 - 2020

10. 梁龙等, 太极拳锻炼防治骨质疏松症效果的 Meta 分析. 中国骨质疏松杂志, 2019(09): 第 1280-1289+1322 页.
11. 曾令烽等, 传统太极功法干预对改善骨密度流失疗效及安全性的系统评价. 中国组织工程研究, 2019(27): 第 4420-4428 页.
12. 何明星等, 太极拳训练对帕金森患者平衡功能影响的 Meta 分析. 世界最新医学信息文摘, 2019(28): 第 6-10 页.
13. 荀长月, 太极拳对糖尿病防治效果的 Meta 分析, 2019, 长江大学.
14. 苏中军与洪平, 太极拳运动对血糖异常患者糖代谢效果的 Meta 分析. 陕西师范大学学报(自然科学版), 2019(03): 第 38-47+125 页.
15. 葛瑶等, 太极拳对 T2DM 患者血糖及脂蛋白代谢水平影响的 Meta 分析. 泰山医学院学报, 2020. 41(2): 第 103-105 页.

Published in English
Reason: the SR with a meta-analysis selected for evidence synthesis was more comprehensive and/or higher quality

2013 - 2014

1. Park, M. and R. Song, *[Effects of Tai Chi on fall risk factors: a meta-analysis].* J Korean Acad Nurs, 2013. **43**(3): p. 341-51.
2. Ni, X., et al., *Efficacy and safety of Tai Chi for Parkinson's disease: a systematic review and meta-analysis of randomized controlled trials.* PLoS One, 2014. **9**(6): p. e99377.
3. Wang, F., et al., *The Effects of Tai Chi on Depression, Anxiety, and Psychological Well-Being: A Systematic Review and Meta-Analysis.* International Journal of Behavioral Medicine, 2014. **21**(4): p. 605-617.

2015 - 2016

4. Song, R., et al., *Effects of t'ai chi on balance: a population-based meta-analysis.* J Altern Complement Med, 2015. **21**(3): p. 141-51.
5. Yang, Y., et al., *The efficacy of traditional Chinese Medical Exercise for Parkinson's disease: a systematic review and meta-analysis.* PLoS One, 2015. **10**(4): p. e0122469.
6. Zheng, G.H., et al., *The Effect of Tai Chi Training on Cardiorespiratory Fitness in Healthy Adults: A Systematic Review and Meta-Analysis.* Plos One, 2015. **10**(2).
7. Chen, Y.W., et al., *The effect of Tai Chi on four chronic conditions-cancer, osteoarthritis, heart failure and chronic obstructive pulmonary disease: a systematic review and meta-analyses.* British Journal of Sports Medicine, 2016. **50**(7): p. 397-U2.
8. Del-Pino-Casado, R., E. Obrero-Gaitan, and R. Lomas-Vega, *The Effect of Tai Chi on Reducing the Risk of Falling: A Systematic Review and Meta-Analysis.* Am J Chin Med, 2016. **44**(5): p. 895-906.
9. Guo, J.B., et al., *Tai Chi for improving cardiopulmonary function and quality of life in patients with chronic obstructive pulmonary disease: a systematic review and meta-analysis.* Clin Rehabil, 2016. **30**(8): p. 750-64.
10. Kong, L.J., et al., *Tai Chi for Chronic Pain Conditions: A Systematic Review and Meta-analysis of Randomized Controlled Trials.* Sci Rep, 2016. **6**: p. 25325.
11. Tao, W.W., et al., *Effects of Acupuncture, Tuina, Tai Chi, Qigong, and Traditional Chinese Medicine Five-Element Music Therapy on Symptom Management and Quality of Life for Cancer Patients: A Meta-Analysis.* J Pain Symptom Manage, 2016. **51**(4): p. 728-747.

2017 - 2018

12. Lian, Z.Y., et al., *Effects of Tai chi on adults with essential hypertension in China: A systematic review and meta-analysis.* European Journal of Integrative Medicine, 2017. **12**: p. 153-162.
13. Ren, X.M., et al., *The Effects of Tai Chi Training in Patients with Heart Failure: A Systematic Review and Meta-Analysis.* Frontiers in Physiology, 2017. **8**.
14. Zou, L.Y., et al., *The Effect of Taichi Practice on Attenuating Bone Mineral Density Loss: A Systematic Review and Meta-Analysis of Randomized Controlled Trials.* International Journal of Environmental Research and Public Health, 2017. **14**(9).
15. Chao, M.Y., et al., *The Effects of Tai Chi on Type 2 Diabetes Mellitus: A Meta-Analysis.* Journal of Diabetes Research, 2018.
16. Hill, K.D., et al., *What works in falls prevention in Asia: a systematic review and meta-analysis of randomized controlled trials.* BMC Geriatr, 2018. **18**(1): p. 3.
17. Winser, S.J., et al., *Does Tai Chi improve balance and reduce falls incidence in neurological disorders? A systematic review and meta-analysis.* Clinical Rehabilitation, 2018. **32**(9): p. 1157-1168.

2019 - 2020

18. Sherrington, C., et al., *Exercise for preventing falls in older people living in the community.* Cochrane Database of Systematic Reviews, 2019(1).
19. Wu, C., et al., *Effects of Mind-Body Exercises on Cognitive Function in Older Adults: A Meta-Analysis.* J Am Geriatr Soc, 2019. **67**(4): p. 749-758.
20. Xia, T.W., et al., *Different training durations and styles of tai chi for glucose control in patients with type 2 diabetes: a systematic review and meta-analysis of controlled trials.* BMC Complement Altern Med, 2019. **19**(1): p. 63.
21. Zhang, S., et al., *The Effect of Tai Chi Chuan on Negative Emotions in Non-Clinical Populations: A Meta-Analysis and Systematic Review.* International Journal of Environmental Research and Public Health, 2019. **16**(17).
22. Duan, L.N., Y.F. Xu, and M. Li, *Effects of Mind-Body Exercise in Cancer Survivors: A Systematic Review and Meta-Analysis.* Evidence-Based Complementary and Alternative Medicine, 2020. **2020**.

23. Guan, Y.Y., et al., *Effects of Tai Chi on Essential Hypertension and Related Risk Factors: A Meta-Analysis of Randomized Controlled Trials.* Journal of Rehabilitation Medicine, 2020. **52**(5).
24. Li, H.J., et al., *The Effect of Tai Chi for Improving Sleep Quality: A Systematic Review and Meta-analysis.* Journal of Affective Disorders, 2020. **274**: p. 1102-1112.
25. Liang, H., et al., *Effects of Tai Chi exercise on cardiovascular disease risk factors and quality of life in adults with essential hypertension: A meta-analysis.* Heart & Lung, 2020. **49**(4): p. 353-363.
26. Palermi, S., et al., *Effectiveness of Tai Chi on Balance Improvement in Type 2 Diabetes Patients: A Systematic Review and Meta-Analysis.* J Aging Phys Act, 2020. **28**(5): p. 787-797.
27. Sherrington, C., et al., *Evidence on physical activity and falls prevention for people aged 65+ years: systematic review to inform the WHO guidelines on physical activity and sedentary behaviour.* Int J Behav Nutr Phys Act, 2020. **17**(1): p. 144.
28. Sherrington, C., et al., *Exercise for preventing falls in older people living in the community: an abridged Cochrane systematic review.* British Journal of Sports Medicine, 2020. **54**(15): p. 885-+.
29. Taylor-Piliae, R. and B.A. Finley, *Benefits of Tai Chi Exercise Among Adults With Chronic Heart Failure A Systematic Review and Meta-Analysis.* Journal of Cardiovascular Nursing, 2020. **35**(5): p. 423-434.
30. Wei, L., et al., *The impact of Tai Chi on cognitive rehabilitation of elder adults with mild cognitive impairment: a systematic review and meta-analysis.* Disabil Rehabil, 2020: p. 1-10.
31. Wu, Y., et al., *Tai Ji Quan as antihypertensive lifestyle therapy: A systematic review and meta-analysis.* J Sport Health Sci, 2020. **1**(20): p. 30038-7.
32. Yang, J.J., et al., *Tai Chi is Effective in Delaying Cognitive Decline in Older Adults with Mild Cognitive Impairment: Evidence from a Systematic Review and Meta-Analysis.* Evidence-Based Complementary and Alternative Medicine, 2020. **2020**.

STAGE 3 SELECTION PROCESS

The following narrative SRs with no meta-analysis were not selected as the populations and outcomes were reported in a SR with a meta-analysis

Reason: SR with no meta-analysis and PICO covered by SR with a meta-analysis (Chinese language databases)

[1] 张志雷与朱东, 太极拳干预心肺慢性病康复的锻炼方案. 中国老年学杂志, 2019(07): 第 1617-1620 页.

[2] 尤思淼等, 太极治疗类风湿性关节炎疗效的系统评价. 吉林医药学院学报, 2020. 41(05): 第 335-337 页.

Reason: SR with no meta-analysis and PICO covered by SR with a meta-analysis (English language databases)

2010 - 2012

1. Jahnke, R., et al., *A comprehensive review of health benefits of qigong and tai chi.* Am J Health Promot, 2010. **24**(6): p. e1-e25.

2. Liu, H. and A. Frank, *Tai chi as a balance improvement exercise for older adults: a systematic review.* J Geriatr Phys Ther, 2010. **33**(3): p. 103-9.

3. Maclaszek, J. and W. Osiski, *The effects of Tai Chi on body balance in elderly people - A review of studies from the early 21st century.* American Journal of Chinese Medicine, 2010. **38**(2): p. 219-229.
4. Wooton, A.C., *An integrative review of Tai Chi research: an alternative form of physical activity to improve balance and prevent falls in older adults.* Orthop Nurs, 2010. **29**(2): p. 108-16; quiz 117-8.
5. Ding, M., *Tai Chi for stroke rehabilitation: a focused review.* Am J Phys Med Rehabil, 2012. **91**(12): p. 1091-6.
6. Ng, S.M., et al., *Tai chi exercise for patients with heart disease: a systematic review of controlled clinical trials.* Alternative therapies in health and medicine, 2012. **18**(3): p. 16-22.
7. Peng, P.W., *Tai chi and chronic pain.* Reg Anesth Pain Med, 2012. **37**(4): p. 372-82.
8. Schleicher, M.M., L. Wedam, and G. Wu, *Review of Tai Chi as an effective exercise on falls prevention in elderly.* Res Sports Med, 2012. **20**(1): p. 37-58.

2013 - 2014

9. Jimenez-Martin, P.J., et al., *A review of Tai Chi Chuan and parameters related to balance.* European Journal of Integrative Medicine, 2013. **5**(6): p. 469-475.
10. Sharma, M. and T. Haider, *Tai Chi as an Alternative or Complementary Therapy for Patients With Depression: A Systematic Review.* Journal of Evidence-Based Integrative Medicine, 2013. **18**(1): p. 43-49.
11. Toh, S.F.M., *A systematic review on the effectiveness of Tai Chi exercise in individuals with Parkinson's disease from 2003 to 2013.* Hong Kong Journal of Occupational Therapy, 2013. **23**(2): p. 69-81.
12. Hartley, L., et al., *Tai chi for primary prevention of cardiovascular disease.* Cochrane Database Syst Rev, 2014(4): p. Cd010366.
13. Li, G.C., H. Yuan, and W. Zhang, *Effects of Tai Chi on health related quality of life in patients with chronic conditions: A systematic review of randomized controlled trials.* Complementary Therapies in Medicine, 2014. **22**(4): p. 743-755.
14. Miller, S.M. and R.E. Taylor-Piliae, *Effects of Tai Chi on cognitive function in community-dwelling older adults: a review.* Geriatr Nurs, 2014. **35**(1): p. 9-19.
15. Nery, R.M., et al., *Tai Chi Chuan for Cardiac Rehabilitation in Patients with Coronary Arterial Disease.* Arquivos Brasileiros De Cardiologia, 2014. **102**(6): p. 588-592.
16. Taylor-Piliae, R.E., *Tai Ji Quan as an exercise modality to prevent and manage cardiovascular disease: A review.* Journal of Sport and Health Science, 2014. **3**(1): p. 43-51.
17. Wayne, P.M., et al., *What do we really know about the safety of tai chi?: A systematic review of adverse event reports in randomized trials.* Arch Phys Med Rehabil, 2014. **95**(12): p. 2470-83.
18. Wayne, P.M., et al., *Can Tai Chi improve vestibulopathic postural control?* Arch Phys Med Rehabil, 2014. **85**(1): p. 142-52.
19. Ye, J.J., et al., *Effects of Tai Chi for Patients with Knee Osteoarthritis: A Systematic Review.* Journal of Physical Therapy Science, 2014. **26**(7): p. 1133-1137.

2015 - 2016

none

2017 - 2018

20. Cwiekala-Lewis, K.J., M. Gallek, and R.E. Taylor-Piliae, *The effects of Tai Chi on physical function and well-being among persons with Parkinson's Disease: A systematic review.* J Bodyw Mov Ther, 2017. **21**(2): p. 414-421.

21. Plummer, M. and C. Bradley, *Tai Chi as a falls prevention strategy in older adults compared to conventional physiotherapy exercise: A review.* International Journal of Therapy and Rehabilitation, 2017. **24**(6): p. 239-247.
22. Zheng, W., et al., *Tai chi for mild cognitive impairment: a systematic review.* Psychogeriatrics, 2017. **17**(6): p. 514-516.
23. Zou, L., et al., *Tai chi for health benefits in patients with multiple sclerosis: A systematic review.* PLoS One, 2017. **12**(2): p. e0170212.
24. Chow, T.H., et al., *The effect of Chinese martial arts Tai Chi Chuan on prevention of osteoporosis: A systematic review.* J Orthop Translat, 2018. **12**: p. 74-84.
25. Pan, Z.J., et al., *The Effects of Tai Chi Intervention on Healthy Elderly by Means of Neuroimaging and EEG: A Systematic Review.* Frontiers in Aging Neuroscience, 2018. **10**.
26. Tong, Y.G., et al., *Effects of Tai Chi on Self-Efficacy: A Systematic Review.* Evidence-Based Complementary and Alternative Medicine, 2018.

2019 - 2020

27. Lim, K.H.L., et al., *The effectiveness of Tai Chi for short-term cognitive function improvement in the early stages of dementia in the elderly: a systematic literature review.* Clinical interventions in aging, 2019. **14**: p. 827-839.
28. Reychler, G., et al., *Efficacy of yoga, tai chi and qi gong on the main symptoms of chronic obstructive pulmonary disease: A systematic review.* Respir Med Res, 2019. **75**: p. 13-25.
29. Chuatrakoon, B., et al., *Balance Impairment and Effectiveness of Exercise Intervention in Chronic Obstructive Pulmonary DiseasedA Systematic Review.* Archives of Physical Medicine and Rehabilitation, 2020. **101**(9): p. 1590-1602.
30. Ford, M.E., et al., *Non-pharmacological treatment for insomnia following acquired brain injury: A systematic review.* Sleep Medicine Reviews, 2020. **50**.
31. Kamieniarz, A., et al., *Tai Chi and Qi Gong therapies as a complementary treatment in Parkinson's disease - a systematic review.* Complement Ther Med, 2020. **56**: p. 102589.
32. Mehdizadeh, S., et al., *A systematic review of center of pressure measures to quantify gait changes in older adults.* Exp Gerontol, 2020. **143**: p. 111170.
33. Park, J., C.A. Krause-Parello, and C.M. Barnes, *A Narrative Review of Movement-Based Mind-Body Interventions Effects of Yoga, Tai Chi, and Qigong for Back Pain Patients.* Holistic Nursing Practice, 2020. **34**(1): p. 3-23.
34. Regauer, V., et al., *Physical therapy interventions for older people with vertigo, dizziness and balance disorders addressing mobility and participation: a systematic review.* BMC Geriatr, 2020. **20**(1): p. 494.
35. Song, Y., et al., *Current Evidence on Traditional Chinese Exercise for Cancers: A Systematic Review of Randomized Controlled Trials.* International Journal of Environmental Research and Public Health, 2020. **17**(14).
36. Walter, A.A., et al., *Complementary and integrative health interventions in post-stroke rehabilitation: a systematic PRISMA review.* Disabil Rehabil, 2020: p. 1-10.
