# Supplementary material for: Determining the safety and effectiveness of Tai Chi: a critical overview of 210 systematic reviews of controlled clinical trials
Source: Syst Rev. 2022 Dec 3;11:260. doi: 10.1186/s13643-022-02100-5 (PMC9719113; doi:10.1186/s13643-022-02100-5)
Supplement: Supplementary file 5 — Additional file 5. AMSTAR 2 quality rating of systematic reviews. [file 13643_2022_2100_MOESM5_ESM.pdf]

Additional file 5: AMSTAR 2 quality rating of systematic reviews

Review ID	1 PICO research question	2 Protocol	3 Study design inclusion rationale	4 Comprehensive literature search	5 Duplicate study selection	6 Duplicate data extraction	7 List excluded articles + rationale	8 Adequate study characteristics	9 Satisfactory RoB assessment	10 Funding source of studies	11 Appropriate meta-analysis	12 Study RoB impact on meta-analysis	13 Study RoB impact on conclusions	14 Heterogeneity explained, discussed	15 Publication bias assessed, discussed	16 Conflict of interest, funding declared	Modified AMSTAR 2 Quality Rating * (*Sensitivity analysis* **)
Buto 2020 [60]	Y	PY	PY	PY	Y	Y	N	Y	PY	N	n/a	n/a	Y	n/a	Y	Y	Moderate (*Low*)
Cheng 2019 [61]	Y	PY	PY	PY	N	Y	N	Y	Y	N	Y	N	Y	Y	N	Y	Low (*Critically low*)
Choo 2020 [62]	Y	N	PY	Y	Y	Y	Y	Y	Y	Y	Y	Y	N	Y	Y	Y	Critically low
Cocchiara 2020 [63]	Y	N	N	PY	Y	Y	N	Y	PY	N	n/a	n/a	N	n/a	n/a	Y	Critically low
Cui 2019 [64]	Y	N	PY	PY	Y	Y	N	Y	Y	N	Y	Y	Y	Y	N	Y	Critically low
Dong 2020 [65]	Y	PY	PY	PY	Y	Y	N	PY	Y	N	N [a]	N	N	Y	Y	Y	Critically low
Gu 2017 [66]	Y	N	PY	PY	N	Y	PY	Y	Y	N	Y	Y	Y	Y	Y	Y	Low (*Critically low*)
Guo 2020 [67]	Y	N	PY	PY	Y	Y	Y	Y	Y	N	Y	Y	Y	Y	Y	Y	Low
Hall 2017 [68]	Y	N	PY	PY	N	Y	PY	Y	Y	Y	Y	Y	Y	Y	Y	N	Low (*Critically low*)
Ho 2013 [69]	Y	N	Y	PY	N	Y	PY	Y	PY	N	n/a	n/a	Y	n/a	n/a	Y	Low (*Critically low*)
Hu 2020 [70]	Y	N	PY	PY	Y	Y	N	Y	Y	N	Y	Y	Y	Y	Y	Y	Low (*Critically low*)
Huang 2017 [73]	Y	N	PY	Y	Y	Y	PY	Y	Y	N	Y	Y	Y	Y	Y	Y	Low (*Critically low*)
Huang 2019 [72]	Y	N	-N	PY	Y	Y	N	Y	PY	N	n/a	n/a	Y	n/a	n/a	Y	Low (*Critically low*)
Huang 2020 [71]	Y	Y	PY	Y	Y	Y	N	Y	Y	Y	N [b]	N	N	Y	Y	Y	Critically low

Yang GY, Hunter J, Bu FL, Hao WL, Zhang H, Wayne PM, Liu JP. Determining the safety and effectiveness of Tai Chi: a critical overview of 210 systematic reviews of controlled clinical trials. *Systematic Reviews* 2022.

Jiang 2018 [74]	Y	N	PY	PY	Y	Y	PY	PY	Y	N	Y	N	Y	N	Y	N	Low (*Critically low*)
Kruisbrink 2020 [75]	Y	Y	PY	PY	Y	Y	N	PY	Y	N	Y	N	N	Y	Y	Y	Low (*Critically low*)
Liu F 2020 [76]	Y	N	N	PY	Y	Y	N	Y	PY	N	N [c]	N	Y	Y	N	Y	Critically low
Liu LZ 2020 [77]	Y	N	PY	PY	Y	Y	N	Y	PY	N	Y	N	N	Y	N	Y	Critically low
Liu WJ 2020 [78]	Y	N	PY	PY	N	N	N	PY	Y	N	N [d]	N	N	Y	Y	N	Critically low
Luo 2020 [79]	Y	Y	PY	PY	N	Y	N	Y	Y	N	Y	Y	Y	Y	Y	Y	Moderate (*Low*)
Lyu 2018 [81]	Y	Y	PY	PY	Y	N	PY	Y	Y	N	Y	Y	Y	Y	Y	Y	Moderate (*Low*)
Lyu 2020 [80]	Y	Y	PY	Y	Y	Y	N	Y	Y	Y	Y	Y	Y	Y	Y	Y	Moderate (*Low*)
Mudano 2019 [82]	Y	Y	N	Y	Y	Y	Y	Y	Y	Y	Y	Y	Y	Y	Y	Y	High
Ni 2019 [83]	Y	N	PY	PY	Y	Y	N	Y	Y	N	Y	Y	Y	Y	Y	Y	Low (*Critically low*)
Pan 2016 [84]	N	N	PY	PY	Y	N	PY	Y	PY	N	Y	N	Y	Y	Y	Y	Low (*Critically low*)
Qin 2019 [85]	Y	Y	PY	PY	Y	Y	N	Y	PY	N	Y	N	N	Y	Y	Y	Low (*Critically low*)
Si 2020 [86]	Y	Y	PY	PY	Y	Y	N	Y	PY	N	Y	Y	N	Y	Y	Y	Low (*Critically low*)
Song 2018 [87]	Y	Y	PY	PY	Y	Y	PY	Y	Y	N	Y	N	N	Y	Y	Y	Low (*Critically low*)
Su 2020 [88]	N	N	PY	N	N	N	N	Y	Y	N	Y	N	N	Y	N	Y	Critically low
Taylor 2017 [89]	N	N	Y	PY	N	N	PY	Y	PY	N	n/a	n/a	Y	n/a	n/a	Y	Low (*Critically low*)
Taylor-Piliae 2020 [90]	Y	N	PY	PY	Y	Y	N	Y	PY	N	Y	N	N	N	N	Y	Critically low
Wang 2010 [93]	Y	N	PY	PY	N	Y	PY	PY	PY	N	Y	Y	N	Y	N	Y	Critically low
Wang 2017 [92]	Y	N	PY	PY	N	Y	PY	Y	Y	N	Y	N	N	Y	Y	Y	Critically low
Wang 2020 [91]	Y	N	PY	PY	Y	Y	N	Y	Y	N	Y	N	N	Y	N	N	Critically low
Wayne 2014 [94]	Y	N	Y	PY	Y	Y	PY	Y	Y	N	Y	Y	Y	Y	N	Y	Critically low
Webster 2016 [95]	Y	N	Y	PY	N	N	PY	PY	N	N	n/a	n/a	Y	n/a	n/a	Y	Critically low
Wu 2020 [96]	Y	N	PY	PY	Y	Y	N	Y	Y	N	Y	Y	Y	Y	Y	N	Low (*Critically low*)
Xiang 2017 [97]	Y	Y	PY	Y	Y	N	PY	Y	Y	N	Y	Y	Y	Y	Y	Y	Moderate (*Low*)
Yin 2014 [98]	Y	N	PY	PY	N	Y	PY	PY	PY	N	Y	Y	Y	Y	Y	N	Low
Yu 2018 [100]	Y	N	PY	PY	N	Y	PY	Y	PY	N	Y	N	N	Y	N	Y	Critically low
Yu 2019 [99]	Y	N	PY	PY	Y	Y	N	PY	Y	N	N [d]	N	Y	Y	Y	Y	Critically low
Zhang 2019 [101]	Y	Y	PY	PY	Y	Y	N	Y	Y	N	Y	Y	Y	Y	Y	Y	Moderate (*Low*)

Yang GY, Hunter J, Bu FL, Hao WL, Zhang H, Wayne PM, Liu JP. Determining the safety and effectiveness of Tai Chi: a critical overview of 210 systematic reviews of controlled clinical trials. *Systematic Reviews* 2022.

Zhang 2020 [102]	Y	N	PY	PY	Y	Y	N	Y	Y	N	Y	N	Y	Y	Y	N	Low (*Critically low*)
Zheng 2015 [103]	Y	N	N	PY	Y	Y	Y	Y	Y	N	Y	Y	Y	Y	Y	Y	Low (*Critically low*)
Zheng 2016 [104]	Y	Y	PY	PY	Y	Y	PY	Y	Y	N	Y	Y	Y	N	Y	Y	Moderate (*Low*)
Zhong 2020 [105]	Y	Y	PY	Y	Y	Y	Y	Y	Y	Y	Y	Y	Y	Y	Y	Y	High
Zhou 2019 [106]	Y	N	PY	PY	Y	Y	N	Y	PY	N	Y	Y	N	Y	Y	Y	Critically low

Y, yes; **N,** no; **PY,** partial yes; **n/a,** not applicable. ***Modified AMSTAR 2 RATING** (see criteria below) ****AMSTAR 2 RATING** if item 7 is deemed a critical item according to Shea et al. [36] and no concessions are given for SRs published before 2019.

[a] Dong 2020 [67], excluded studies from the meta-analyses to reduce statistical heterogeneity. This is not recommended except as a sensitivity analysis. Also, there was no information about the potential causes of the heterogeneity or how excluding these studies impacted the estimates of effect.
[b] Huang 2020 [73], meta-analysis of falls risk reported Risk Ratios rather than Rate Ratios. No concerns with meta-analyses of falls risk factor outcomes.
[c] Liu F 2020 [78], pooled the results of RCTs and NRSIs without a rationale or any subgroup/sensitivity analysis.
[d] Liu WJ 2020 [80] and Yu 2019 [101], probable data extraction/transformation errors.

Modified AMSTAR 2 rating criteria

Critical Items: 2, 4, 9, 11, 13 and 15. **Non-critical Items:** 1, 3, 5, 6, 7, 8, 10, 12, 14, 16.

- **High quality:** No applicable critical flaws and no or one non-critical weakness
- **Moderate quality:** No applicable critical flaws
- **Low quality:** One applicable critical flaw with or without non-critical weaknesses
- **Critically low quality:** More than one applicable critical flaw with or without non-critical weaknesses

Item 7 was deemed a non-critical item as this was only introduced to the revised PRISMA 2020 statement that was published in early 2021 [34]. For SRs published before 2019, item 7 was not downrated if they met the 2009 PRISMA reporting standards for excluded articles (Preferred Reporting Items for Systematic Reviews and Meta-analyses) statement reporting standards [38] as this item was introduced in late September 2017.

Item 3 was rated as PY when the SR only included RCTs and/or quasi-RCTs and as such it could be “inferred from what the authors actually wrote” that the rationale for the study design inclusion/exclusion criteria was to provide the most reliable and complete summary of the effects. Item 3 was rated N when other NRSI study designs were included, and no rationale was provided and could it not be “inferred from a careful reading of the complete study report”.
