# Supplementary material for: Determining the safety and effectiveness of Tai Chi: a critical overview of 210 systematic reviews of controlled clinical trials
Source: Syst Rev. 2022 Dec 3;11:260. doi: 10.1186/s13643-022-02100-5 (PMC9719113; doi:10.1186/s13643-022-02100-5)
Supplement: Supplementary file 6 — Additional file 6. GRADE appraisal of the certainty of the evidence. [file 13643_2022_2100_MOESM6_ESM.pdf]

Additional file 6: GRADE appraisal of the certainty of the evidence

Study ID	Outcome	Estimate of effect (95% CI)	Risk of bias	Inconsistency *(sensitivity analysis)*	Indirectness	Imprecision *(sensitivity analysis)*	Publication Bias	Evidence Certainty * *(sensitivity analysis)*
Cheng 2019 [61]	Fibromyalgia impact questionnaire 12-16 weeks (4 RCTs)	SMD -0.61 (-0.90 to -0.31)	serious	not serious	not serious	not serious *(serious)*	not serious	MODERATE *(LOW)*
	FIQ 24-32 weeks (2 RCTs)	SMD -0.49 (-1.56 to 0.58)	serious	serious	not serious	very serious	not serious	VERY LOW
	Pain PROMs (3 RCTs)	SMD -0.88 (-1.58 to -0.18)	serious	serious	not serious	serious	not serious	VERY LOW
Choo 2020 [62]	QoL - physical PROMs (6 RCTs)	SMD 0.46 (0.13 to 0.80)	serious	not serious	not serious	not serious	not serious	MODERATE
	QoL - mental health PROMs (6 RCTs)	SMD 0.21 (0.03 to 0.39)	serious	not serious	not serious	not serious	not serious	MODERATE
	Depressive symptoms (5 RCTs)	SMD -0.42 (-0.84 to -0.01)	serious	serious	not serious	not serious	not serious	LOW
	Time up and go (5 RCTs)	MD -0.2 (-0.66 to 0.26)	serious	not serious	not serious	not serious *(serious)*	not serious	MODERATE *(LOW)*
Cui 2019 [64]	Serious adverse events - AE (TC vs active interventions) (15 RCTs)	RD 0.0 (-0.02 to 0.02)	not serious	not serious	not serious	very serious	not serious	LOW
	Non-serious AE (TC vs active interventions) (15 RCTs)	RD 0.01 (-0.01 to 0.03)	not serious	not serious	not serious	serious	not serious	MODERATE
	TC related AE (TC vs active interventions) (15 RCTs)	RD 0.0 (-0.01 to 0.02)	not serious	not serious	not serious	serious	not serious	MODERATE
	Serious AE (TC vs inactive interventions) (9 RCTs)	RD -0.03 (-0.06 to 0.00)	not serious	not serious	not serious	serious	not serious	MODERATE
	Non-serious AE (TC vs inactive interventions) (9 RCTs)	RD 0.03 (-0.00 to 0.07)	not serious	not serious	not serious	serious	not serious	MODERATE
	TC related AE (TC vs inactive interventions) (9 RCTs)	RD 0.0 (-0.01 to 0.02)	not serious	not serious	not serious	serious	not serious	MODERATE
Gu 2017 [66]	6-minute walk test (10 RCTs)	MD 51 (30.49 to 71.5)	very serious	serious	not serious	not serious	not serious	VERY LOW
	Minnesota Living with Heart Failure Questionnaire - MLHFQ (8 RCTs)	MD -10.4 (-14.4 to -6.3)	very serious	serious	not serious	not serious	not serious	VERY LOW
	Left ventricular ejection fraction (7 RCTs)	MD 7.7 (3.6 to 11.9)	very serious	serious	not serious	not serious	not serious	VERY LOW
Guo 2020 [67]	Forced expiratory volume in 1 second - FEV1 (3 RCTs)	MD 0.13 (0.06 to 0.20)	serious	not serious	not serious	not serious *(serious)*	not serious	MODERATE *(LOW)*

	FEV1 (5 RCTs)	MD 0.06 (-0.01 to 0.14)	serious	not serious	not serious	serious	not serious	LOW
	6-minute walk time - 6-MWT (TC vs control) (6 RCTs)	MD 24.3 (6.3 to 42.3)	serious	serious	not serious	not serious *(serious)*	not serious	LOW *(VERY LOW)*
	6MWT (TC vs exercise) (6 RCTs)	MD 7.5 (2.1 to 12.3)	serious	not serious	not serious	not serious	not serious	MODERATE
	St George Respiratory Questionnaire - SGRQ (TC vs control) (3 RCTs)	MD -8.7 (-14.6 to -2.7)	serious	not serious	not serious	not serious *(serious)*	not serious	MODERATE *(LOW)*
	SGRQ (TC vs exercise) (4 RCTs)	MD -1.9 (-4.6 to 0.7)	serious	not serious	not serious	not serious	not serious	MODERATE
Hall 2017 [68]	Pain SF-36 15 weeks (1 RCTs)	SMD -1.85 (-2.73 to -0.97)	very serious	not serious	not serious	very serious *(serious)*	not serious	VERY LOW
Hu 2020 [70]	Western Ontario and McMaster Universities Arthritis Index - WOMAC pain (14 RCTs)	SMD -0.69 (-0.95 to -0.44)	serious	not serious	not serious	not serious	not serious	MODERATE
	WOMAC stiffness (12 RCTs)	SMD -0.65 (-0.98 to -0.33)	serious	serious	not serious	not serious	not serious	LOW
	WOMAC physical function (13 RCTs)	SMD -0.92 (-1.16 to -0.69)	serious	not serious	not serious	not serious	not serious	MODERATE
	QoL mental health, SF-36 (5 RCTs)	SMD 0.26 (0.06 to 0.45)	serious	not serious	not serious	not serious	not serious	MODERATE
	QoL physical, SF-36 (5 RCTs)	SMD 0.48 (0.28 to 0.68)	serious	not serious	not serious	not serious	not serious	MODERATE
	Arthritis self-efficacy scale (4 RCTs)	SMD 0.27 (0.06 to 0.48)	serious	not serious	not serious	not serious *(serious)*	not serious	MODERATE *(LOW)*
Huang 2017 [73]	Rate of people who fell (no. of fallers) (16 RCTs)	RR 0.80 (0.72 to 0.88)	not serious	not serious	not serious	not serious	serious	MODERATE
	Incidence of falls (no. falls) (15 RCTs)	RR 0.69 (0.60 to 0.80)	not serious	not serious	not serious	not serious	serious	MODERATE
Huang 2020 [71]	Single Leg Stance (8 RCTs)	MD 5.8 (0.62 to 10.90)	serious	very serious	not serious	not serious	not serious	VERY LOW
	Berg balance scale (4 RCTs)	MD 1.0 (0.2 to 1.9)	serious	not serious	not serious	not serious	not serious	MODERATE
	Time up and go (6 RCTs)	MD -0.71 (-0.88 to -0.54)	serious	not serious	not serious	not serious *(serious)*	not serious	MODERATE *(LOW)*
Jiang 2018 [74]	VO2max (4 RCTs)	SMD 2.2 (0.81 to 3.63)	very serious	serious	not serious	not serious *(serious)*	not serious	VERY LOW
Kruisbrink 2020 [75]	Fear of falling (6 RCTs)	SMD.B -1.05 (-1.60 to -0.50)	very serious	serious	not serious	not serious	serious	VERY LOW
Luo 2020 [79]	Pain, 3 weeks (2 RCTs)	SMD 0.25 (-0.02 to 0.51)	serious	not serious	not serious	serious	not serious	LOW

	Pain, 12 weeks (4 RCTs)	SMD 0.3 (0.08 to 0.51)	serious	not serious	not serious	not serious *(serious)*	not serious	MODERATE *(LOW)*
Liu LZ 2020 [77]	Fatigue (TC vs control) 3 months (2 RCTs)	MD -0.46 (-1.09 to 0.17)	serious	not serious	not serious	serious	not serious	LOW
	Fatigue (TC vs control) 6 months (2 RCTs)	MD -0.16 (-0.98 to 0.67)	serious	not serious	not serious	serious	not serious	LOW
	Fatigue (TC+Ucare/Rehab vs Ucare/Rehab) 3 months (2 RCTs)	SMD -0.91 (-1.30 to -0.53)	serious	not serious	not serious	serious	not serious	LOW
Lyu 2018 [81]	Activities of daily living - Barther Index (2 RCTs)	MD 9.9 (6.8 to 13.0)	serious	not serious	not serious	serious	not serious	LOW
	Fugl-Meyer Assessment FMA - lower extremity (3 RCTs)	MD 2.8 (0.95 to 4.56)	serious	serious	not serious	serious	not serious	VERY LOW
	FMA - upper extremity (2 RCTs)	MD 8.3 (4.7 to 11.8)	serious	not serious	not serious	serious	not serious	LOW
	FMA - all four limb (2 RCTs)	MD 4.5 (1.9 to 7.1)	serious	not serious	not serious	serious	not serious	LOW
	Berg Balance Scale (2 RCTs)	MD 5.2 (3.4 to 7.1)	serious	not serious	not serious	serious	not serious	LOW
	Time up and go (4 RCTs)	MD 2.6 (1.8 to 3.4)	serious	not serious	not serious	serious	not serious	LOW
Lyu 2020 [80]	Depression (6 RCTs)	SMD 0.36 (0.10 to 0.61)	very serious	not serious	not serious	not serious	not serious	LOW
Mudano 2019 [82]	Pain, Visual Analog Scale, 12 weeks (2 RCTs)	SMD -0.95 (-1.41 to -0.49)	very serious	not serious	not serious	very serious *(serious)*	not serious	VERY LOW
	Disease activity, DAS-28-ESR, 12 weeks (1 RCTs)	MD -0.40 (-1.10 to 0.30)	very serious	not serious	not serious	very serious *(serious)*	not serious	VERY LOW
	Function, Health Assessment Questionnaire - HAQ, 12 weeks (2 RCTs)	MD -0.33 (-0.79 to 0.12)	very serious	serious	not serious	very serious *(serious)*	not serious	VERY LOW
Ni 2019 [83]	QoL physical domain (9 RCTs)	SMD 0.34 (0.09 to 0.59)	very serious	not serious	not serious	not serious	not serious	LOW
	QoL psychological Domain (9 RCTs)	SMD 0.60 (0.12 to 1.08)	very serious	serious	not serious	not serious	not serious	VERY LOW
	QoL social relationship domain (8 RCTs)	SMD 0.26 (0.25 to 0.77)	very serious	serious	not serious	not serious	not serious	VERY LOW
	Sleep quality (3 RCTs)	SMD 0.26 (-0.02 to 0.53)	very serious	serious	not serious	serious	not serious	VERY LOW
Pan 2016 [84]	Total Cholesterol (6 RCTs)	MD -7.7 (-17.3 to 1.4)	serious	serious	not serious	serious	not serious	VERY LOW
	Triglycerides (6 RCTs)	MD -16.8 (-31.3 to -2.4)	serious	not serious	not serious	not serious	not serious	MODERATE

	High-density lipoprotein cholesterol (5 RCTs)	MD 0.46 (-0.71 to 1.64)	serious	not serious	not serious	not serious *(serious)*	not serious	MODERATE *(LOW)*
	Low density lipoprotein cholesterol (4 RCTs)	MD -1.61 (-16.25 to 13.02)	serious	very serious	not serious	Serious *(very serious)*	not serious	VERY LOW
Qin 2019 [85]	Pain VAS 1-10 scale (TC vs control) (3 RCTs)	MD -1.2 (-2.3 to -1.1)	serious	serious	not serious	not serious *(serious)*	not serious	LOW *(VERY LOW)*
	Pain VAS 1-10 scale (TC + usual care vs usual care) (5 RCTs)	MD -1.1 (-1.3 to -0.9)	serious	not serious	not serious	not serious	not serious	MODERATE
Si 2020 [86]	Pittsburgh Sleep Quality Index - PSQI, healthy (10 RCTs)	SMD -0.68 (-1.06 to -0.31)	serious	serious	not serious	not serious	not serious	LOW
	PSQI, chronic disease (15 RCTs)	SMD -0.39 (-0.74 to -0.05)	serious	serious	not serious	not serious	not serious	LOW
Song 2018 [87]	Cancer related fatigue, lung cancer <8 weeks (2 RCTs)	SMD -0.5 (-0.83 to -0.18)	very serious	not serious	not serious	serious	not serious	VERY LOW
	Cancer related fatigue, prostate cancer <8 weeks (1 RCTs)	SMD 0.01 (-0.51 to 0.52)	very serious	not serious	not serious	very serious	not serious	VERY LOW
Su 2020 [88]	knee extensor muscle strength (60°/sec) (2 RCTs)	MD 17.5 (-12.0 to 47.0)	serious	serious	not serious	very serious *(serious)*	not serious	VERY LOW
	Knee flexor muscle strength (60°/sec) (2 RCTs)	MD 22.1 (1.1 to 43.2)	serious	not serious	not serious	very serious *(serious)*	not serious	VERY LOW *(LOW)*
	Knee flexor muscle strength 1-RM (2 RCTs)	MD 3.3 (2.1 to 4.4)	serious	not serious	not serious	serious	not serious	LOW
	Knee extensor muscle strength 1-RM (4 RCTs)	MD 0.90 (0.34 to 1.45)	serious	not serious	not serious	not serious *(serious)*	not serious	MODERATE *(LOW)*
Taylor-Piliae 2020 [90]	QoL mental health PROMs, hypertension (3 RCTs)	SMD 0.13 (NI)	not serious	not serious	not serious	serious	not serious	MODERATE
	QoL physical PROMs, hypertensive (3 RCTs)	SMD 0.47 (NI)	not serious	not serious	not serious	not serious	not serious	HIGH
	Psychological distress, chronic heart failure (2 RCTs)	SMD -0.58 (-0.95 to -0.22)	not serious	not serious	not serious	serious	not serious	MODERATE
Wang 2020 [91]	QoL general, PROMs (6 RCTs)	SMD 1.23 (0.56 to 1.89)	serious	serious	not serious	not serious	not serious	LOW
	QoL physical, SF-36 (3 RCTs)	MD 5.9 (1.1 to 10.8)	serious	serious	not serious	not serious *(serious)*	not serious	LOW *(VERY LOW)*
	QoL psychological, SF-36 (3 RCTs)	MD 2.2 (-1.2 to 5.6)	serious	not serious	not serious	serious	not serious	LOW
Wang 2010 [93]	Stress PROMs (4 RCTs)	SMD 0.97 (0.06 to 1.87)	very serious	very serious	not serious	not serious *(serious)*	not serious	VERY LOW
	Mood / affect PROMs (2 RCTs)	SMD 0.25 (-0.04 to 0.53)	very serious	not serious	not serious	serious	not serious	VERY LOW

Wayne 2014 [94]	Executive function (Tai Chi vs inactive control) (4 RCTs)	SMD 0.90 (0.03 to 1.78)	not serious	serious	not serious	not serious	not serious	MODERATE
	Executive function (TC vs exercise) (2 RCTs)	SMD 0.51 (0.17 to 0.85)	not serious	not serious	not serious	serious	not serious	MODERATE
Wang 2017 [92]	Physical function SF-36 (4 RCTs)	MD -1.8 (-5.2 to 1.6)	serious	not serious	not serious	serious	not serious	LOW
	Bodily pain SF-36 (3 RCTs)	MD -3.6 (-6.6 to -0.6)	serious	not serious	not serious	not serious *(serious)*	not serious	MODERATE *(LOW)*
	General health SF-36 (3 RCTs)	MD -5.1 (-7.6 to -2.6)	serious	not serious	not serious	not serious *(serious)*	not serious	MODERATE *(LOW)*
	Vitality SF-36 (3 RCTs)	MD -5.7 (-8.5 to -2.8)	serious	not serious	not serious	not serious *(serious)*	not serious	MODERATE *(LOW)*
	Mental health SF-36 (4 RCTs)	MD -2.5 (-4.8 to -0.2)	serious	not serious	not serious	not serious *(serious)*	not serious	MODERATE *(LOW)*
	Social function SF-36 (3 RCTs)	MD -2.2 (-5.0 to 0.6)	serious	not serious	not serious	serious	not serious	LOW
Wu 2020 [96]	6-minute walk time (5 RCTs)	SMD 1.3 (0.50 to 2.11)	serious	serious	not serious	not serious	not serious	LOW
	Left ventricular ejection fraction (5 RCTs)	SMD 1.0 (0.43 to 1.57)	serious	serious	not serious	not serious	not serious	LOW
Xiang 2017 [97]	Fatigue PROMs (10 RCTs)	SMD -0.45 (-0.70 to -0.20)	serious	not serious	not serious	not serious	not serious	MODERATE
	Vitality PROMs (4 RCTs)	SMD 0.63 (0.20 to 1.07)	very serious	not serious	not serious	not serious	not serious	LOW
	Sleep PROMs (3 RCTs)	SMD -0.32 (-0.61 to -0.04)	very serious	not serious	not serious	not serious *(serious)*	not serious	LOW *(VERY LOW)*
	Depression PROMs (7 RCTs)	SMD -0.58 (-1.04 to -0.11)	very serious	serious	not serious	not serious	not serious	VERY LOW
Yin 2014 [98]	Depression scales (25 RCTs)	SMD 0.36 (0.19 to 0.53)	not serious	not serious	not serious	not serious	not serious	HIGH
	Anxiety scales (11 RCTs)	SMD 0.34 (0.02 to 0.66)	not serious	serious	not serious	not serious	not serious	MODERATE
Yu 2018 [100]	Unified Parkinson's Disease Rating III: Motor (8 RCTs)	MD -3.7 (-5.7 to -1.7)	not serious	serious	not serious	not serious	not serious	MODERATE
	Time up and go (7 RCTs)	SMD -0.5 (-0.88 to -0.11)	not serious	not serious	not serious	not serious	not serious	HIGH
	Berg balance scale (6 RCTs)	SMD 0.85 (0.44 to 1.27)	not serious	not serious	not serious	not serious *(serious)*	not serious	HIGH *(MODERATE)*
	Parkinson's Disease Questionnaire (3 RCTs)	SMD -0.75 (-1.45 to -0.04)	not serious	not serious	not serious	not serious *(serious)*	not serious	HIGH *(MODERATE)*

Zhang 2019 [101]	Spine bone mineral density - BMD (6 RCTs)	MD 0.04 g/cm2 (0.02 to 0.06)	serious	not serious	not serious	not serious *(serious)*	not serious	MODERATE *(LOW)*
	Femur BMD (3 RCTs)	MD 0.04 g/cm2 (0.01 to 0.06)	serious	not serious	not serious	serious	not serious	LOW
	Spine BMD (2 RCTs)	MD 0.16 g/cm2 (0.09 to 0.23)	serious	not serious	not serious	serious	not serious	LOW
	Femur BMD (2 RCTs)	MD 0.16 g/cm2 (0.04 to 0.29)	serious	serious	not serious	serious	not serious	VERY LOW
Zhang 2020 [102]	Global cognitive function (5 RCTs)	MD 0.29 (-0.61 to 0.74)	not serious	not serious	not serious	not serious	not serious	HIGH
	Memory - Delayed Recall Test (4 RCTs)	MD 0.37 (0.13 to 0.61)	not serious	not serious	not serious	not serious	not serious	HIGH
	Performance - Digit Span Test (4 RCTs)	MD 0.03 (-0.16 to 0.22)	not serious	not serious	not serious	not serious	not serious	HIGH
Zheng 2015 [103]	Incidence of nonfatal stroke over 1 - 2 years (2 RCTs)	RR 0.11 (0.01 to 0.85)	serious	not serious	not serious	serious	not serious	LOW
	Incidence of fatal stroke over 1 - 2 years (2 RCTs)	RR 0.33 (0.05 to 2.05)	serious	not serious	not serious	Serious *(very serious)*	not serious	LOW *(VERY LOW)*
Zheng 2016 [104]	Negative symptoms - Positive and Negative Syndrome Scale and Scale - PANSS (6 RCTs)	SMD -0.87 (-1.51 to -0.24)	serious	serious	not serious	not serious	not serious	LOW
	Positive symptoms - PANSS (5 RCTs)	SMD -0.09 (-0.44 to 0.26)	serious	not serious	not serious	not serious *(serious)*	not serious	MODERATE *(LOW)*
	Discontinuation rate (4 RCTs)	RR 0.06 (0.23 to 1.40)	serious	not serious	not serious	very serious	not serious	VERY LOW
Zhou 2019 [106]	Glycosylated haemoglobin - HbA1c % (14 RCTs)	MD -0.88 (-1.45 to -0.31)	serious	serious	not serious	not serious	not serious	LOW
	Systolic blood pressure (5 RCTs)	MD -10.0 mmHg (-15.8 to -4.3)	serious	not serious	not serious	not serious *(serious)*	not serious	MODERATE *(LOW)*
	Diastolic blood pressure (5 RCTs)	MD -4.9 mmHg (-8.2 to -1.5)	serious	not serious	not serious	not serious *(serious)*	not serious	MODERATE *(LOW)*
	QoL physical function (5 RCTs)	MD 7.1 (0.79 to 13.4)	serious	serious	not serious	not serious *(serious)*	not serious	LOW *(VERY LOW)*
	QoL bodily pain (5 RCTs)	MD 4.3 (0.8 to 7.8)	serious	not serious	not serious	not serious *(serious)*	not serious	MODERATE *(LOW)*
Zhong 2020 [105]	Systolic blood pressure - SBP (TC vs inactive control) (9 RCTs)	MD -14.8 (-19.6 to -10.0)	serious	serious	not serious	not serious	not serious	LOW
	Diastolic blood pressure - DBP (TC vs inactive control) (9 RCTs)	MD -7.0 (-9.1 to -5.0)	serious	not serious	not serious	not serious	not serious	MODERATE
	SBP (TC vs exercise) (5 RCTs)	MD -7.9 (-14.2 to -1.7)	serious	serious	not serious	not serious *(serious)*	not serious	LOW *(VERY LOW)*

Yang GY, Hunter J, Bu FL, Hao WL, Zhang H, Wayne PM, Liu JP. Determining the safety and effectiveness of Tai Chi: a critical overview of 210 systematic reviews of controlled clinical trials. *Systematic Reviews* 2022.

DBP (TC vs exercise) (5 RCTs)	MD -3.9 (-6.5 to -1.2)	serious	not serious	not serious	not serious *(serious)*	not serious	MODERATE *(LOW)*
SBP (medication) 15 RCTs)	MD -9.1 (-14.0 to -4.1)	serious	serious	not serious	not serious	not serious	LOW
DBP (medication) (15 RCTs)	MD -5.6 (-14.0 to -4.1)	serious	serious	not serious	not serious	not serious	LOW

CI: confidence interval, **RD:** risk difference, **MD:** mean difference, **MID:** minimally important difference, **SMD:** standardised mean difference, **SMD.B:** regression co-efficient for standardised mean difference, **AE:** adverse effects, **6MWT**: 6-minute walk distance/test, **BMD:** bone mineral density, **BP:** blood pressure, **PANSS:** positive and negative syndrome scale, **Rehab:** rehabilitation programs, **Ucare:** usual care, conventional treatment, standard medical care, **TC:** Tai Chi; **RCTs:** Randomized controlled trials, **QoL:** Quality of Life

*** GRADE RUBRIC** summary of specific thresholds, ranges and criteria used for the assessments

1. **Risk of bias (RoB)**

   Overall assessment of RoB: Low RoB: ≥75% RCTs in the SR were assessed as low RoB in all three categories - randomisation/selection bias, assessor blinding, and missing data; OR if no information (NI) about the categories, ≥75% RCTs were rated overall as low RoB; **Moderate RoB:** ≥75% RCTs were assessed as low RoB in one or two categories - randomisation, assessor blinding, and missing data; OR if NI about categories, ≥75% RCTs were rated overall as moderate RoB; **High RoB:** <75% RCTs were assessed overall as low RoB in all three categories - randomisation, assessor blinding, and missing data; OR if NI about categories <75% RCTs were rated overall as low or moderate RoB.

   i. Very serious: high RoB & no sensitivity analysis or the effect estimate is unstable with sensitivity analysis.
   ii. Serious: high RoB, however, the effect estimate stable with sensitivity analysis when high RoB of RCTs excluded or only low RoB RCTs included; OR moderate RoB & no sensitivity analysis or the effect estimate is unstable with sensitivity analysis.
   iii. Not serious: low RoB; OR moderate RoB, however, the effect estimate is stable with sensitivity analysis when only low RoB of RCTs included.

   NOTE: cut-off of 75% and the most important RoB categories - randomisation, assessor blinding - were informed by algorithm developed by Pollock et al. [40].

   Risk of bias sensitivity analysis (not performed)

   Alternate options for a sensitivity analysis such as incorporating more RoB domains or assessing the RoB for each estimate were not possible due to pragmatic constraints.

2. **Inconsistency**

   i. Very serious: very high heterogeneity ($I^2 \geq 90\%$) & mixed direction of results, +/- appreciable non-overlap in CIs (confirm with visual inspection of Forest plot) or NI.
   ii. Serious: considerable heterogeneity I^2 between 76% to 89%; OR $I^2 \geqslant 90\%$, however, all RCTs favour one direction & CIs mostly overlap or if subgroup/sensitivity analysis is indicated then reduces ($I^2 \leq 75\%$) and estimate of effect is stable.
   iii. Not serious: no heterogeneity; OR acceptable heterogeneity ($I^2 \leq 75\%$).

   NOTE: heterogeneity cut off set at $I^2 \leq 75\%$ as per algorithm developed by Pollock et al. [38]; subgroup analysis deemed unreliable or not indicated if <10 RCTs, and 'one-out' study sensitivity analysis unreliable if <6 RCTs.

   Inconsistency sensitivity analysis

   i. Very serious: heterogeneity I^2 >75% & mixed direction of results +/- appreciable non-overlap in CIs; OR $I^2 \geq 90\%$ and NI.
   ii. Serious: heterogeneity $I^2 > 75\%$, however, all RCTs favour one direction & CIs mostly overlap, or if subgroup/sensitivity analysis is indicated then reduces $I^2 \leq 75\%$ with stable estimates of effects; OR I^2 between 31-75% & mixed direction of results +/- appreciable non-overlap in CIs; OR NI.
   iii. Not serious: no heterogeneity; OR heterogeneity $I^2 \leq 30\%$ and τ^2 test P>0.1; OR heterogeneity I^2 between 31-75% and all RCTs favour Tai Chi, or CIs are overlapping, or subgroup/sensitivity analysis is indicated and $I^2 \leq 40\%$ with stable estimates of effect.

   NOTE: subgroup analysis deemed unreliable or not indicated if <10 RCTs, and 'one-out' study sensitivity analysis unreliable if <6 RCTs.

3. **Indirectness**

   NOTE: all estimates of effect were assessed as 'not serious' as all participants, interventions and outcomes were directly relevant to the research question.

4. **Imprecision**

Optimum information size (OIS): is met if trial sequential analysis conducted and information size is reached; otherwise for continuous data OIS is met if no. participants in meta-analysis ≥ 200; for relative/absolute risk OIS is met if >4,000 participants & no. events >100, alternatively calculate OIS (α = 0.05; β = 0.02; 25% RRR) or use Fig.4/5 in GRADE guidelines 5.2.4. to estimate OIS [33].

Important benefit and harm included: SMD ±0.5; MD ±minimal clinically important difference (MCID); OR/RR/HR <0.75 and >1.25; ARR treatment outcomes and non-serious AEs ±0.1; ARR serious AEs ±0.01.

i. Very serious: no. participants in meta-analysis of continuous data <100; OR OIS is not met & 95%CI includes no effect and both important benefit and harm included.
ii. Serious: OIS is not met & 95% CI excludes no effect; OR OIS is met, however, 95% CI includes no effect & important benefit or harm included.
iii. Not serious: OIS is met & 95% CI excludes no effect; OR OIS is met, however, 95% CI includes no effect & important benefit and harm excluded.

NOTE: OIS cut offs of 100 and 200 for continuous data as per algorithm developed by Pollock et al. [40].

Imprecision sensitivity analysis

Optimum information size (OIS): is met as per above, except for continuous data OIS is met if no. participants in meta-analysis ≥ 400.

Important benefit and harm included: is unchanged.

i. Very serious: OIS is not met & 95% CI includes no effect and both important benefit and harm included.
ii. Serious: OIS is not met & 95% CI excludes no effect; OR OIS is met, however, 95% CI includes no effect & important benefit or harm included.
iii. Not serious: OIS is met & 95% CI excludes no effect; OR OIS is met, however, 95% CI includes no effect & important benefit and harm excluded.

NOTE: cut off for OIS met ≥400 for continuous data informed by GRADE [118].

5. **Publication bias**
   i. Serious: assessed as ‘strongly suspected’ based on funnel plot and/or statistical test; OR not assessed, however, assessed as ‘strongly suspected’ for another meta-analysis in the same systematic review; OR not assessed despite >10 studies in the meta-analysis and at least half of the studies have a sample size <100.
   ii. Not serious: not assessable <10 studies in the meta-analysis; OR assessed as not present or probably not present.

Publication bias sensitivity analysis (not performed)

Language bias was not assessed as studies published in languages other than English are more likely to report findings that favour traditional and complementary medicine interventions and may increase, rather than decrease funnel plot asymmetry [119] since they typically have small sample sizes [7].

REFERENCES

118. Guyatt, G.H., et al., *GRADE guidelines 6. Rating the quality of evidence--imprecision.* J Clin Epidemiol, 2011. **64**(12): p. 1283-93.
119. Dobrescu, A.I., et al., *Restricting evidence syntheses of interventions to English-language publications is a viable methodological shortcut for most medical topics: a systematic review.* J Clin Epidemiol, 2021. **137**: p. 209-217.
